# Promyelocytic leukemia (PML) nuclear bodies (NBs) induce latent/quiescent HSV-1 genomes chromatinization through a PML NB/Histone H3.3/H3.3 Chaperone Axis

**DOI:** 10.1371/journal.ppat.1007313

**Published:** 2018-09-20

**Authors:** Camille Cohen, Armelle Corpet, Simon Roubille, Mohamed Ali Maroui, Nolwenn Poccardi, Antoine Rousseau, Constance Kleijwegt, Olivier Binda, Pascale Texier, Nancy Sawtell, Marc Labetoulle, Patrick Lomonte

**Affiliations:** 1 Univ Lyon, Université Claude Bernard Lyon 1, CNRS UMR 5310, INSERM U 1217, LabEx DEVweCAN, Institut NeuroMyoGène (INMG), team Chromatin Assembly, Nuclear Domains, Virus, Lyon, France; 2 Institut de Biologie Intégrative de la Cellule (I2BC), Département de Virologie, Gif-sur-Yvette, France; 3 Université Paris Sud, Centre Hospitalier Universitaire de Bicêtre, Service d'Ophthalmologie, Le Kremlin-Bicêtre, France; 4 Division of Infectious Diseases, Cincinnati Children’s Hospital Medical Center, Cincinnati, Ohio, United States of America; University of Wisconsin-Madison, UNITED STATES

## Abstract

Herpes simplex virus 1 (HSV-1) latency establishment is tightly controlled by promyelocytic leukemia (PML) nuclear bodies (NBs) (or ND10), although their exact contribution is still elusive. A hallmark of HSV-1 latency is the interaction between latent viral genomes and PML NBs, leading to the formation of viral DNA-containing PML NBs (vDCP NBs), and the complete silencing of HSV-1. Using a replication-defective HSV-1-infected human primary fibroblast model reproducing the formation of vDCP NBs, combined with an immuno-FISH approach developed to detect latent/quiescent HSV-1, we show that vDCP NBs contain both histone H3.3 and its chaperone complexes, i.e., DAXX/ATRX and HIRA complex (HIRA, UBN1, CABIN1, and ASF1a). HIRA also co-localizes with vDCP NBs present in trigeminal ganglia (TG) neurons from HSV-1-infected wild type mice. ChIP and Re-ChIP show that vDCP NBs-associated latent/quiescent viral genomes are chromatinized almost exclusively with H3.3 modified on its lysine (K) 9 by trimethylation, consistent with an interaction of the H3.3 chaperones with multiple viral loci and with the transcriptional silencing of HSV-1. Only simultaneous inactivation of both H3.3 chaperone complexes has a significant impact on the deposition of H3.3 on viral genomes, suggesting a compensation mechanism. In contrast, the sole depletion of PML significantly impacts the chromatinization of the latent/quiescent viral genomes with H3.3 without any overall replacement with H3.1. vDCP NBs-associated HSV-1 genomes are not definitively silenced since the destabilization of vDCP NBs by ICP0, which is essential for HSV-1 reactivation in vivo, allows the recovery of a transcriptional lytic program and the replication of viral genomes. Consequently, the present study demonstrates a specific chromatin regulation of vDCP NBs-associated latent/quiescent HSV-1 through an H3.3-dependent HSV-1 chromatinization involving the two H3.3 chaperones DAXX/ATRX and HIRA complexes. Additionally, the study reveals that PML NBs are major actors in latent/quiescent HSV-1 H3.3 chromatinization through a PML NB/histone H3.3/H3.3 chaperone axis.

## Author summary

An understanding of the molecular mechanisms contributing to the persistence of a virus in its host is essential to be able to control viral reactivation and its associated diseases. Herpes simplex virus 1 (HSV-1) is a human pathogen that remains latent in the PNS and CNS of the infected host. The latency is unstable, and frequent reactivations of the virus are responsible for PNS and CNS pathologies. It is thus crucial to understand the physiological, immunological and molecular levels of interplay between latent HSV-1 and the host. Promyelocytic leukemia (PML) nuclear bodies (NBs) control viral infections by preventing the onset of lytic infection. In previous studies, we showed a major role of PML NBs in favoring the establishment of a latent state for HSV-1. A hallmark of HSV-1 latency establishment is the formation of PML NBs containing the viral genome, which we called “viral DNA-containing PML NBs” (vDCP NBs). The genome entrapped in the vDCP NBs is transcriptionally silenced. This naturally occurring latent/quiescent state could, however, be transcriptionally reactivated. Therefore, understanding the role of PML NBs in controlling the establishment of HSV-1 latency and its reactivation is essential to design new therapeutic approaches based on the prevention of viral reactivation.

## Introduction

Herpes simplex virus 1 (HSV-1) is a human pathogen with neurotropic tropism and the causal agent of cold sores and more severe CNS pathologies such as encephalitis [1]. After the initial infection, HSV-1 remains latent in neuronal ganglia with the main site of latency being the trigeminal (or Gasserian) ganglion (TG). Two transcriptional programs are associated with HSV-1 infection, the lytic cycle and latency, which differ by the number and degree of viral gene transcription. The lytic cycle results from the sequential transcription of all viral genes (approximately 80) and leads to the production of viral progeny. The latency phase, occurring exclusively in neurons, is limited to the abundant expression of the so-called Latency Associated Transcripts (LATs), although physiologically a transitory expression of a limited number of lytic genes is not excluded, making latency a dynamic process[2–4].

Following lytic infection of epithelial cells at the periphery, the viral particle enters the axon termini of the innervating neurons by fusion of its envelope with the plasma membrane. The nucleocapsid is then carried into the neuron body by retrograde transport, most likely through the interaction of viral capsid components [5] with microtubule-associated proteins such as dynein and dynactin [6–10]. Once the nucleocapsid reaches the cell body, the virus phenotype changes from the one at the axon termini because most of the outer tegument proteins, including VP16, a viral transactivator that is essential for the onset of lytic infection, remain at the axonal tip [11–13]. Hence, when the viral DNA is injected into the neuron nucleus, it does not automatically benefit from the presence of VP16 to initiate transcription of lytic genes. Rather, the balance between lytic and latent transcriptional programs most likely depends on stochastic events and on undescribed neuron-associated factor(s) able to initiate the transcription of VP16 through the activation of neuro-specific sequences present in the VP16 promoter [14]. Without VP16 synthesis, transcription of the viral genes encoding ICP4 (the major transactivator protein) and ICP0 (a positive regulator of viral and cellular gene transcription) is hampered. Hence, ICP4 and ICP0 gene transcription is unlikely to reach the required level to produce these two proteins above a threshold that would favor onset of the lytic cycle. Therefore, in neurons, commitment of the infectious process towards the lytic cycle or latency depends on a race between opposing infection-prone viral components and cellular features with antiviral activities.

Promyelocytic leukemia (PML) nuclear bodies (NBs) (also called ND10) are proteinaceous entities involved in the control of viral infection as part of the cell and nucleus-associated intrinsic antiviral response but also through innate immunity associated with the interferon (IFN) response [15]. Our recent studies have shown that PML NBs tightly associate with incoming HSV-1 genomes in the nucleus of infected TG neurons in mouse models and in primary TG neuron cultures [16,17]. Hence, PML NBs reorganize in structures called viral DNA-containing PML NBs (vDCP NBs), which are formed at early times during the process of HSV-1 latency establishment and persist during latency *per se* in a large subset of latently infected neurons in a mouse model of infection [16]. The entrapment of incoming wild type HSV-1 genomes by PML NBs is not a unique feature of latency, because it has recently been shown to occur prior to the onset of lytic infection, as part of the intrinsic antiviral response. HSV-1 genomes trapped in the vDCP NBs are transcriptionally repressed for LATs production [16]. It is known that HSV-1 latency, at least in the mouse model and possibly in humans, is heterogeneous at the single neuron level for the expression of LATs [16,18–25]. Therefore, although at the entire TG level HSV-1 latency could be a dynamic process from a transcriptional perspective, at the single neuron level, a strict, transcriptionally silent, quiescence can be observed, and vDCP NB-containing neurons are major contributors of this latent/quiescent HSV-1 state. In humans, vDCP NB-like structures have also been observed in latently infected TG neurons [17], suggesting that vDCP NBs are probably molecular hallmarks of the HSV-1 latency process, including in the natural host.

Another essential feature of HSV-1 latency is the chromatinization of its 150-kb genome, which enters the nucleus of the infected cells as a naked/non-nucleosomal dsDNA [26–28]. Once the viral genome is injected into the nucleus of the infected neuron, it circularizes, associates with nucleosomes to become chromatinized, and remains as an episome that is unintegrated into the host cell genome [29]. Although latent viral genomes sustain chromatin regulation, essentially through post-translational modifications of associated histones [30–34] not much is known about the mechanisms that induce their chromatinization and which specific histone variants are associated with these latent genomes. In mammals, specific H3 histone variants that differ by a few amino acid residues can influence chromatin compaction and transcriptional activity of the genome. The histone variant H3.3, a specific variant of the histone H3 that is expressed throughout the cell cycle, is deposited in a replication-independent manner, in contrast to H3.1 ([35] and for review [36]). Interestingly, death domain associated protein 6 (DAXX) and α-thalassemia mental retardation X-linked protein (ATRX), initially identified as a transcriptional repressor and a chromatin remodeler, respectively, are constitutively present in PML NBs, and have now been identified as H3.3-specific histone chaperones [37–39]. The other histone H3.3 specific chaperone complex is called the HIRA complex, which is composed of Histone cell cycle regulator (HIRA), Ubinuclein 1 (UBN1), Calcineurin-binding protein 1 (CABIN1), and Anti-silencing function protein 1 homolog A (ASF1a) [35]. The HIRA complex does not normally accumulate in PML NBs except upon entry of the cell into senescence [40,41]. The histone variant H3.3 itself localizes in PML NBs in proliferating and senescent cells, linking PML NBs with the chromatin assembly pathway independently of replication [42–44]. Because vDCP NBs contain DAXX and ATRX [16,17,45], their involvement in the chromatinization of incoming HSV-1 genomes and/or long-term maintenance of chromatinized HSV-1 genomes is thus plausible.

Human primary fibroblasts or adult mouse primary TG neuron cultures infected through their cell body with a replication-defective HSV-1 virus, *in*1374, which is unable to synthesize functional ICP4 and ICP0 under specific temperature conditions, enable the establishment of a latent/quiescent state for HSV-1 [17,45–47]. The latent/quiescent state of HSV-1 in human primary fibroblasts has also been reproduced using engineered HSV-1 unable to express major immediate early genes [48,49]. We have shown that this latent/quiescent state is linked to the formation of vDCP NBs, mimicking, at least concerning this particular structural aspect, the latency observed in a subset of neurons in mouse models and in humans [16,17]. Here, using the *in*1374-based *in cellula* model of infection, we showed that vDCP NBs contained not only the DAXX and ATRX proteins but also all the components of the HIRA complex and H3.3 itself. HIRA was also found to co-localize with vDCP NBs in neurons of TG harvested from HSV-1 wild type infected mice. Both DAXX/ATRX and HIRA complex components were found to interact with multiple viral loci by chromatin immunoprecipitation (ChIP). Using the same approaches, we showed that latent/quiescent viral genomes were almost exclusively chromatinized with H3.3, itself modified on its lysine (K) 9 by trimethylation (H3.3K9me3). Most interestingly, we found that H3.3 chromatinization of the viral genomes was dependent on intact PML NBs, demonstrating that PML NBs contribute to an essential part of the chromatinization of the latent/quiescent HSV-1 genomes. Overall, this study shows that the chromatinization of latent HSV-1 involves a PML NB/histone H3.3/histone H3.3 chaperone axis that confers and probably maintains chromatin marks on viral genomes.

## Results

### The HIRA complex components accumulate in the vDCP NBs

**Fig 1:**
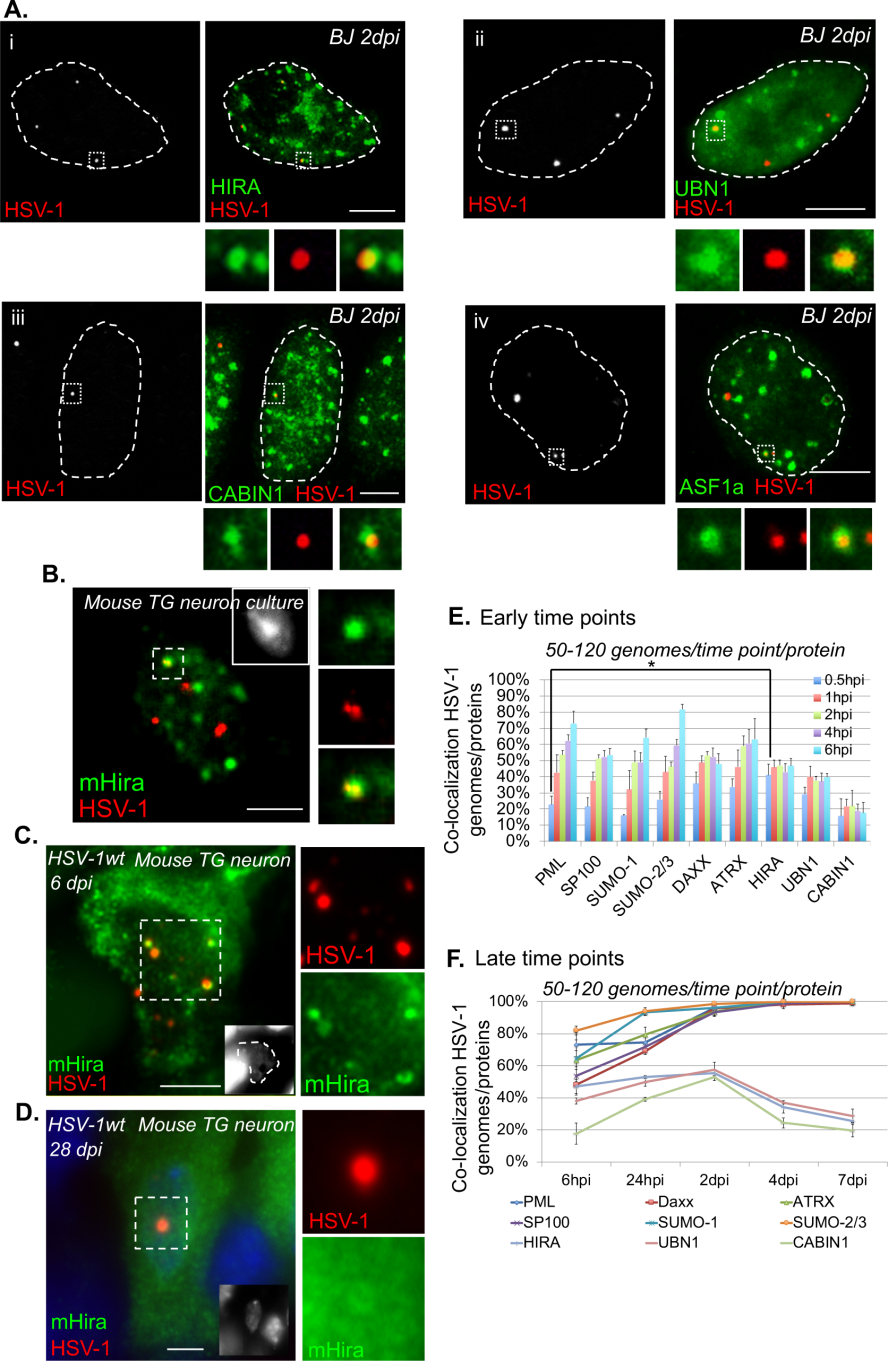
Latent/quiescent HSV-1 genomes co-localize with the HIRA complex. (A) Immuno-FISH performed in human primary fibroblasts (BJ cells) infected for 2 days with the replication-defective HSV-1 virus *in*1374. HIRA (i), UBN1 (ii), CABIN1 (iii), ASF1a (iv) (green), and HSV-1 genomes (red) were detected. Scale bars = 5 μm. (B) Immuno-FISH performed in adult mouse primary TG neuron cultures infected for 2 days with *in*1374. Mouse Hira (mHira, green), HSV-1 genomes (red), and nucleus (inset, gray) are detected. Scale bar = 5 μm. (C) Same as (B) but in TG neurons from 6-day HSV-1wt-infected mice. Scale bars = 10 μm. (D) Same as (B) in TG neurons of 28-day HSV-1wt-infected mice. Scale bars = 10 μm. (E) Quantifications of immuno-FISH performed in BJ cells infected with *in*1374 at early times pi. Data represent the percentage of co-localization between incoming HSV-1 genomes and representative proteins of the PML NBs (PML, Sp100, SUMO-1, SUMO 2/3) or H3.3 chaperone complex proteins (DAXX, ATRX, HIRA, UBN1, CABIN1). Means from three independent experiments ± SD. The Student’s *t*-test was applied to assess the significance of the results. * = p< 0.05 (see S2 Table for data). (F) Same as (E) but at late times pi. Means from three independent experiments ± SD (see S3 Table for data).

The formation of vDCP NBs is a molecular hallmark of HSV-1 latency, and vDCP NBs are present in infected neurons from the initial stages of latency establishment to latency *per se* in mouse models [16,17]. Using a previously established *in vitro* latency system [46] consisting of human primary fibroblast cultures infected with a replication-deficient virus (hereafter called *in*1374) unable to express functional VP16, ICP4 and ICP0, we and others were able to reproduce the formation of vDCP NBs [17,45]. We first verified that vDCP NBs induced in BJ and other human primary cells infected with *in*1374 at a non-permissive temperature of 38.5°C, contained, in addition to PML, the proteins constitutively found in the PML NBs, i.e., Sp100, DAXX, ATRX, SUMO-1 and SUMO-2/3 (S1Ai to S1vi Fig, and S1 Table). The DAXX/ATRX complex is one of the two chaperones of the histone variant H3.3 involved in the replication-independent chromatinization of specific, mostly heterochromatic, genome loci [39]. Interestingly, HSV-1 enters the nucleus of the infected cell as a naked/non-nucleosomal dsDNA and remains during latency as a circular chromatinized episome unintegrated in the host genome [29,50]. It is thus tempting to speculate that the presence of DAXX/ATRX in the vDCP NBs could be linked to a process of initiation and/or maintenance of chromatinization of the latent/quiescent viral genome. The other H3.3 chaperone is known as the HIRA complex and was initially described as specific for the replication-independent chromatinization of euchromatin regions [35,51]. Remarkably, proteins of the HIRA complex are able to bind in a sequence-independent manner to a naked/non-nucleosomal DNA [52], suggesting that the HIRA complex could also participate in the recognition and chromatinization of the incoming naked HSV-1 genome. We thus investigated the localization of all members of the HIRA complex and found that they co-localized with the latent/quiescent HSV-1 genomes at 2 days post-infection (dpi) in BJ and other human primary cells (Fig 1Ai to 1iv, S1 Table). To confirm that the co-localization of members of the HIRA complex with the latent/quiescent HSV-1 could be reproduced in neuronal cells, adult mouse TG neuron cultures were infected with *in*1374 for 2 days before performing immuno-FISH. Mouse Hira, which was the only protein of the HIRA complex detectable in mouse cells, showed a clear co-localization with a subset of viral genomes (Fig 1B). To analyze whether this co-localization was also reproducible *in vivo*, immuno-FISH was performed on TG samples from HSV-1-infected mice. Hira was found to co-localize with HSV-1 genomes with the “multiple acute”/vDCP NB pattern (see [17,53,54]) in TG neurons from infected mice at 6 dpi (Fig 1C) but not with the “single”/vDCP NB pattern (see [16,53,54]) at 28 dpi (Fig 1D), suggesting a dynamic association of this protein with the vDCP NBs.

To analyze this dynamic association, co-localization between incoming HSV-1 genomes and proteins of the PML NBs or of the HIRA complex was quantified at early times from 30 min pi to 6 hpi using a synchronized infection procedure (Fig 1E and S2 Table). Except for the proteins of the HIRA complex, the percentages of co-localization increased with time. Interestingly, at 30 min pi, the percentage of co-localization of HSV-1 genomes with HIRA was significantly higher than with PML (41±7% vs 23±5%, p value = 0.03, Student’s *t-test*, S2 Table). Although DAXX and ATRX also showed, on average, a greater percentage of co-localization with HSV-1 genomes (36±7% and 34±5% at 30 min, respectively) compared with PML, the data were not significant (S2 Table). Moreover, a recent study showed the interaction of at least PML, SUMO-2, and Sp100 with incoming HSV-1 genomes as soon as 1 hpi, which supports our data [55]. The absence of co-localization of mouse Hira with viral genomes with the “single”/vDCP NB pattern in mouse TG neurons at 28 dpi suggested that longer infection times could lead to loss of proteins of the HIRA complex from the vDCP NBs. Infection of BJ cells were reiterated as above, but this time quantifications were performed from 24 hpi to 7 dpi. Strikingly, whereas all the proteins permanently present in the PML NBs remained co-localized with a maximum of 100% of the latent/quiescent HSV-1 genome from 2 dpi until 7 dpi, proteins of the HIRA complex peaked at 2 dpi, and then their co-localization decreased at longer times pi, confirming the temporary association of the HIRA complex with the vDCP NBs (Fig 1F, and S3 Table).

**Fig 2:**
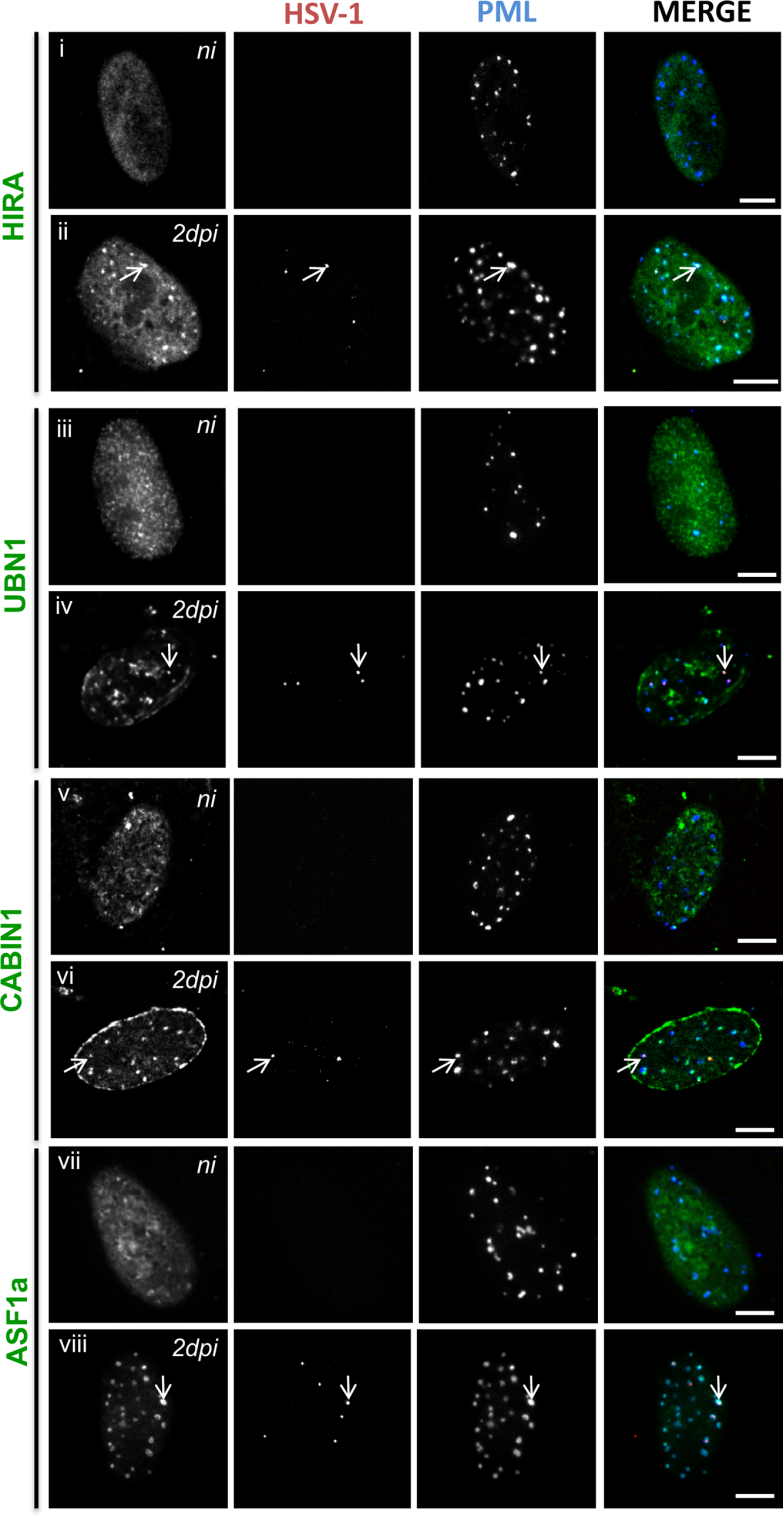
HSV-1 infection induces the accumulation of HIRA complex proteins in PML NBs and co-localization with latent/quiescent HSV-1 genomes in vDCP NBs. Immuno-FISH performed in BJ cells not infected (ni) (i, iii, v, vii) or infected for 2 days (ii, iv, vi, viii) with *in*1374. HIRA (i and ii), UBN1 (iii and vi), CABIN1 (v and vi), ASF1a (vii and viii) (gray, green), HSV-1 genomes (gray, red), and PML (gray, blue) were detected. Arrows indicate examples of the detection of HIRA complex proteins in vDCP NBs. Scale bars = 5 μm.

To definitively show that proteins of the HIRA complex were present in vDCP NBs, immuno-FISH were performed on BJ cells infected for 2 days with *in*1374 to detect a member of the HIRA complex, HSV-1 genomes, and PML. Strikingly, while proteins of the HIRA complex showed predominant nucleoplasmic staining in non-infected cells (Fig 2i, 2iii, 2v and 2vii), in infected cells all the proteins clearly and systematically accumulated in PML NBs (Fig 2ii, 2iv, 2vi and 2viii). The accumulation of HIRA in PML NBs following infection by HSV-1 has recently been suggested to be part of an interferon-induced antiviral mechanism [56]. Consequently, HIRA, UBN1, CABIN1 and ASF1a co-localized with the latent/quiescent HSV-1 genomes in vDCP NBs (arrows in Fig 2ii, 2iv, 2vi and 2viii). Altogether, these data show that both DAXX/ATRX and HIRA complexes are present within vDCP NBs in neuronal and non-neuronal cells, suggesting a role for these two complexes in latent/quiescent HSV-1 chromatinization.

### Histone H3.3 chaperones interact with incoming viral genome

**Fig 3:**
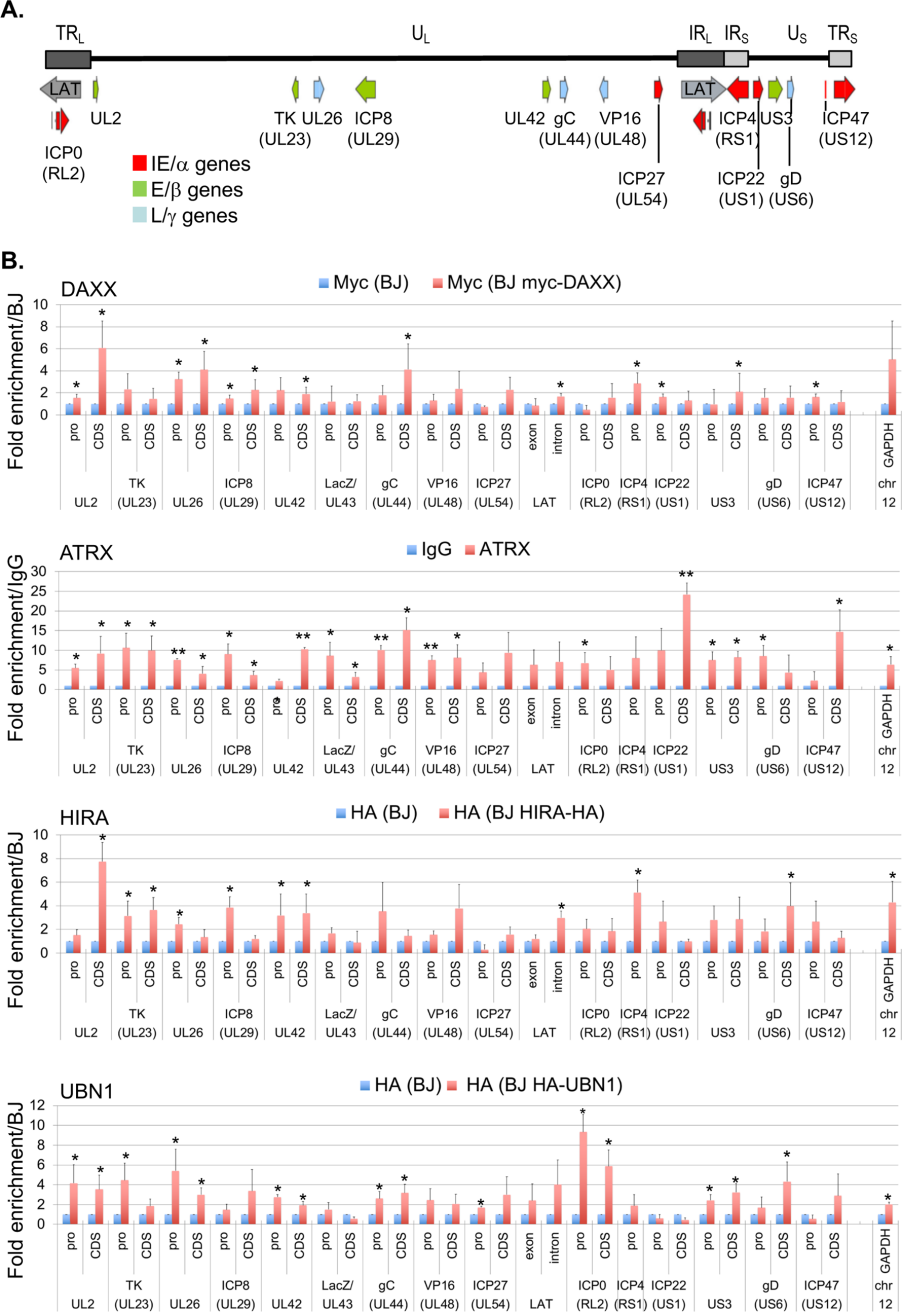
Components of the DAXX/ATRX and HIRA complexes associate with latent/quiescent HSV-1 genomes. (A) Schematic localization of the HSV-1 genome and of the loci analyzed by quantitative PCR (qPCR). UL: Unit Long, US: Unit Short, TRL: Terminal Repeat Long, TRS: Terminal Repeat Short, IRL: Inverted Repeat Long, IRS: Inverted Repeat Short. Immediate early (IE/α) genes (red), early (E/β) genes (green), late (L/γ) genes (blue). (B) Chromatin immunoprecipitation (ChIP) associated with qPCR performed in *in*1374-infected normal BJ cells or *in*1374-infected BJ cells expressing tagged versions of DAXX, HIRA, or UBN1. Infections were performed for 24 h. Anti-myc (DAXX) or anti-HA (HIRA and UBN1) antibodies were used. For ATRX, a native antibody was used, and the results were compared to ChIP with IgG as control. Means from three independent experiments ± SD. Student’s *t*-test was applied to assess the significance of the results. * = p< 0.05.

The co-localization of proteins of the DAXX/ATRX and HIRA complexes with the incoming HSV-1 genomes and their presence in the vDCP NBs suggested an interaction of these proteins with the viral genome, as shown recently for HIRA on a small subset of viral loci [56]. Since DAXX, HIRA, and UBN1 antibodies were not efficient in the ChIP experiments, we constructed cell lines stably expressing myc-DAXX, HIRA-HA, or HA-UBN1 by transduction of BJ cells with lentiviral- vectors (S2 Fig). Cells were infected with *in*1374 at 38.5°C and harvested 24 hpi to perform ChIP-qPCR on multiple loci spread over the entire HSV-1 genome, representing promoter or core regions (CDS) of genes of all kinetics (IE/α, E/β, L/γ) (Fig 3A). Cellular glyceraldehyde 3-phosphate dehydrogenase (GAPDH) locus was used as a positive control for enrichment. Significant enrichments compared to controls were detected for all proteins on several viral loci independently of their promoter or CDS status, and with no obvious discrepancy regarding the gene kinetic, confirming the potential interaction of these proteins all along the latent/quiescent HSV-1 genomes. Our immuno-FISH data anticipated a gradual interaction of the four proteins with the incoming viral genomes at early times post infection (see Fig 1E). To verify if this could be measured, ChIP-qPCR were performed at 30 min pi, 2 hpi and 6 hpi, using the same experimental conditions as for the immuno-FISH at early times pi (with synchronization of the infection, see Materials and Methods). The data showed a tendency for a weak interaction with the viral genomes at 30 min pi then an increase at 2 hpi and 6 hpi, although with a lot of variability, probably highlighting the dynamic of the biological events occurring during the initial stages of the infection process (S3B Fig). ATRX showed the more regular increase in its interaction with viral genomes from 30 min to 24 hpi. Overall, the ChIP data correlate with the immuno-FISH, and suggest a dynamic process for the interaction between HSV-1 genomes and proteins of the DAXX/ATRX and HIRA complexes, initiating early after the viral DNA enters the nucleus, and remaining at later times when vDCP NBs are structured.

### H3.3 is present in the vDCP NBs and interacts with latent/quiescent HSV-1 genomes

**Fig 4:**
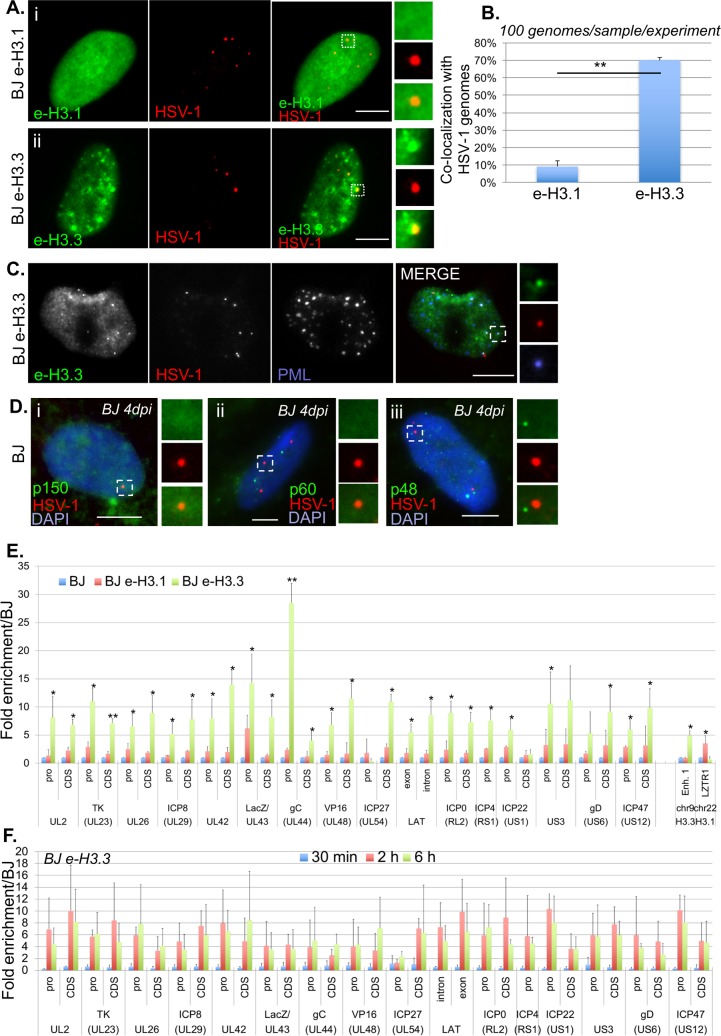
The histone variant H3.3 co-localizes and interacts with latent/quiescent HSV-1 genomes. (A) Immuno-FISH performed in e-H3.1 (i) or e-H3.3 (ii)-expressing BJ cells infected for 2 days with *in*1374. E-H3.1 or e-H3.3 (green), and HSV-1 genomes (red) were detected. Scale bars = 5 μm. (B) Quantification of the immuno-FISH performed in (A). Means from three independent experiments ± SD. The Student’s *t*-test was applied to assess the significance of the results. ** = p< 0.01. (C) Immuno-FISH performed in e-H3.3-expressing BJ cells infected for 2 days with *in*1374. E-H3.3 (gray, green), HSV-1 (gray, red), and PML (gray, blue) were detected. Scale bar = 5 μm. (D) Immuno-FISH performed in normal BJ cells infected for 2 days with *in*1374. H3.1 CAF chaperone complex proteins p150 (i), p60 (ii), and p48 (iii) (green), and HSV-1 genomes (red) were detected. Nuclei were detected with DAPI (blue). Scale bars = 5 μm. (E) ChIP performed in *in*1374-infected normal BJ cells (blue), *in*1374-infected e-H3.1 (red) or e-H3.3 (green) expressing BJ cells. Infections were performed for 24 h. Anti-HA antibody was used for ChIP experiments. Analyzed viral loci were described previously. Cellular loci Enhancer 1 (Enh.1), and leucine-zipper-like transcriptional regulator 1 (LZTR1) are positive controls for deposition of H3.3 and H3.1, respectively. Means from three independent experiments ± SD. The Student’s t-test was applied to assess the significance of the results. * = p< 0.05, ** = p< 0.01. (F) ChIP performed in *in*1374-infected e-H3.3-expressing BJ cells at early times pi, 30 min (blue), 2 h (red), 6 h (green). Anti-HA antibody was used for ChIP experiments. Analyzed viral loci were described previously. Means from three independent experiments ± SD.

The co-localization of the two histone H3.3 chaperone complexes with viral genomes suggested the chromatinization of HSV-1 latent/quiescent genomes with the histone variant H3.3. Histones H3.1 and H3.3 differ by only 5 amino acids, and, in our hands, no suitable antibody is available that can distinguish both histones by IF or IF-FISH. We thus constructed lentivirus-transduced BJ cell lines expressing a tagged version of either histone (e-H3.1 and e-H3.3) (see Materials and Methods, and [43], S4A and S4B Fig). We confirmed that ectopic expression of e-H3.3 led to its accumulation in PML NBs unlike e-H3.1 (S4C Fig) [42,43]. *In*1374 infection of BJ e-H3.1/3-expressing cells led to the co-localization of viral genomes almost exclusively with e-H3.3 (Fig 4Ai, 4ii and 4B). Importantly, e-H3.3 co-localized with HSV-1 genomes together with PML in vDCP NBs (Fig 4C). The lack of co-localization of viral genomes with e-H3.1 was in agreement with the absence of any of the H3.1 CAF-1 chaperone subunits (p150, p60, p48) in the vDCP NBs (Fig 4D, S1 Table). To confirm that e-H3.3, unlike e-H3.1, interacted with HSV-1 genomes, ChIP-qPCR were conducted on the same loci as those analyzed above. As expected, e-H3.3, but not e-H3.1, was highly enriched on the viral genome independently of the examined locus (Fig 4E). Several cellular loci were analyzed as controls for specific enrichments with H3.3 (Enhancer 1 (Enh.1) on chromosome 9, [57]), or H3.1 (leucine-zipper-like transcriptional regulator 1 (LZTR1) on chromosome 22, GEO accession number GSM1135044). Similar data were obtained for all other canonical histones (S5 Fig), confirming that H3.3 association with latent/quiescent HSV-1 genomes is in a nucleosomal context. To confirm that the discrepancy between the binding of e-H3.3 and e-H3.1 to viral genomes was not due to the ectopic expression of histones, we performed similar experiments using antibodies against native proteins. One specific antibody for H3.3, and suitable for ChIP experiments has previously been described [58]. We performed ChIP using antibodies against native H3.1/2 or H3.3 in normal BJ cells infected for 24 h by *in*1374. The results were similar to those obtained in infected BJ e-H3.3 using the anti-HA antibody (S6 Fig). These data confirmed that no bias was introduced in the ChIP experiments due to the use of tagged histones, and that latent/quiescent HSV-1 genomes are chromatinized with H3.3. The gradual interaction of the four proteins of the H3.3 chaperone complexes with the incoming viral genomes anticipated similar changes in the interaction of H3.3. ChIP-qPCR were performed at 30 min pi, 2 hpi and 6 hpi, using the same experimental conditions as above. The data showed an overall weak or lack of, H3.3 association with the viral genomes at 30 min pi, followed by an increased interaction at 2 hpi and 6 hpi. These data show that the H3.3 chromatinization of the incoming HSV-1 genomes is progressive and follows a kinetic that matches that observed with the proteins of the H3.3 chaperone complexes. The data also fit with recently published data showing the interaction of incoming viral genomes with canonical histones by 2 hpi [55].

### The H3.3K9me3 chromatin mark is predominantly found on vDCP NBs-associated latent/quiescent HSV-1 genomes

**Fig 5:**
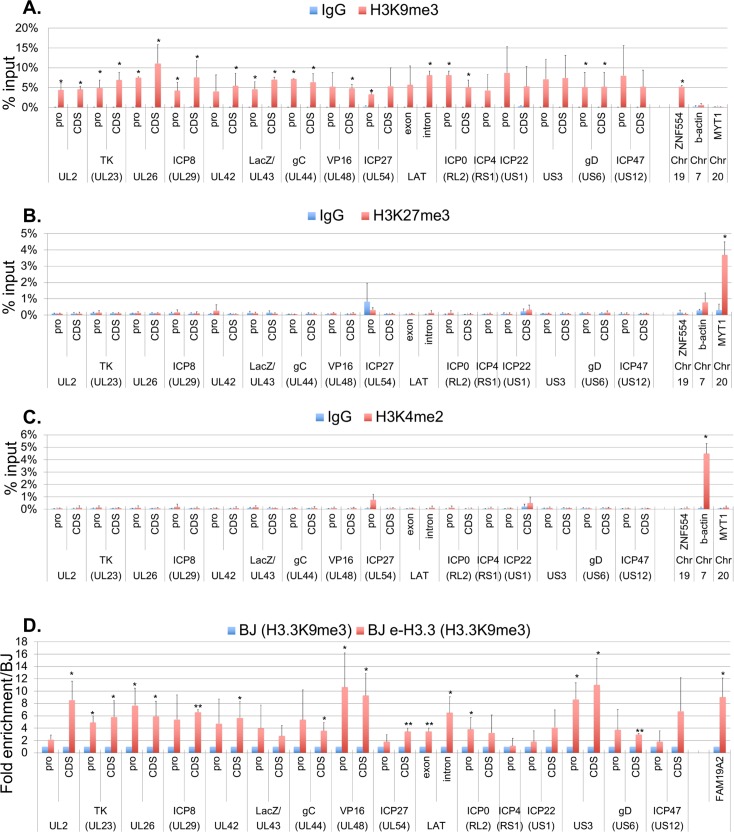
The H3.3K9me3 chromatin mark is present on vDCP NBs-associated latent/quiescent HSV-1 genomes. (A-C) ChIP against H3K9me3 (A), H3K27me3 (B), and H3K4me2 (C) performed in *in*1374-infected e-H3.3-expressing BJ cells at 24 hpi. Cellular loci zinc-finger protein 554 (ZNF554), ß-actin, and myelin transcription factor 1(MYT1) are positive controls for association with H3K9me3, H3K4me2 and H3K27me3, respectively. Means from three independent experiments ± SD. The Student’s t-test was applied to assess the significance of the results. * = p< 0.05, ** = p< 0.01. (D) Re-ChIP performed in in1374-infected normal BJ cells (blue), and e-H3.3-expressing BJ cells (red) at 24 hpi. First antibody: anti-H3K9me3, and second antibody: anti-HA against e-H3.3. Cellular locus, family with sequence similarity 19 member A2 (FAM19A2) is a positive control for association with H3.3K9me3 (GEO accession numbers: GSM1358809 (H3.3), and GSM1289412 (H3K9me3)). Means from three independent experiments ± SD. The Student’s t-test was applied to assess the significance of the results. * = p< 0.05, ** = p< 0.01.

Both constitutive (H3K9me2, H3K9me3) and facultative (H3K27me3) heterochromatin marks have been found on various loci on latent HSV-1 genomes *in vivo* [31,33,34]. To analyze the association of these marks with vDCP NBs-associated latent/quiescent HSV-1 genomes, ChIP were performed targeting H3K9me3, H3K27me3 and one euchromatic mark H3K4me2 as a control (Fig 5). HSV-1 genomes were exclusively associated with H3K9me3 (Fig 5A), matching previous results obtained using quiescent viruses [59,60]. In contrast H3K27me3 (Fig 5B) or H3K4me2 (Fig 5C) marks were not detected. Cellular genes previously described for their association with either marks were analyzed for the specificity of the antibodies used (Zinc-finger protein 554 (ZNF554)/H3K9me3 [61]); myelin transcription factor 1 (MYT1)/H3K27me3 ([62]; Actin/H3K4me2). To confirm that the K9me3 modification is present on H3.3 associated with the HSV-1 genomes, Re-ChIP was performed targeting first H3K9me3 then e-H3.3 in infected BJ and BJ e-H3.3 (Fig 5D). An overall enrichment for H3.3 from samples initially ChIPed with the H3K9me3 antibody was detected only in BJ e-H3.3 and not BJ cells, with 17 viral loci over 31 (55%) showing significant enrichment. The cellular locus, family with sequence similarity 19 member A2 (FAM19A2) specifically enriched with H3.3K9me3 (GEO accession numbers: GSM1358809 (H3.3), and GSM1289412 (H3K9me3)) was used as positive control. These data show that (i) the Re-ChIP experiment is specific of e-H3.3 and (ii) H3.3K9me3 is indeed associated with the vDCP NB-associated HSV-1 latent/quiescent genomes.

### Simultaneous inactivation of DAXX/ATRX and HIRA complexes affects HSV-1 genomes chromatinization with H3.3

**Fig 6:**
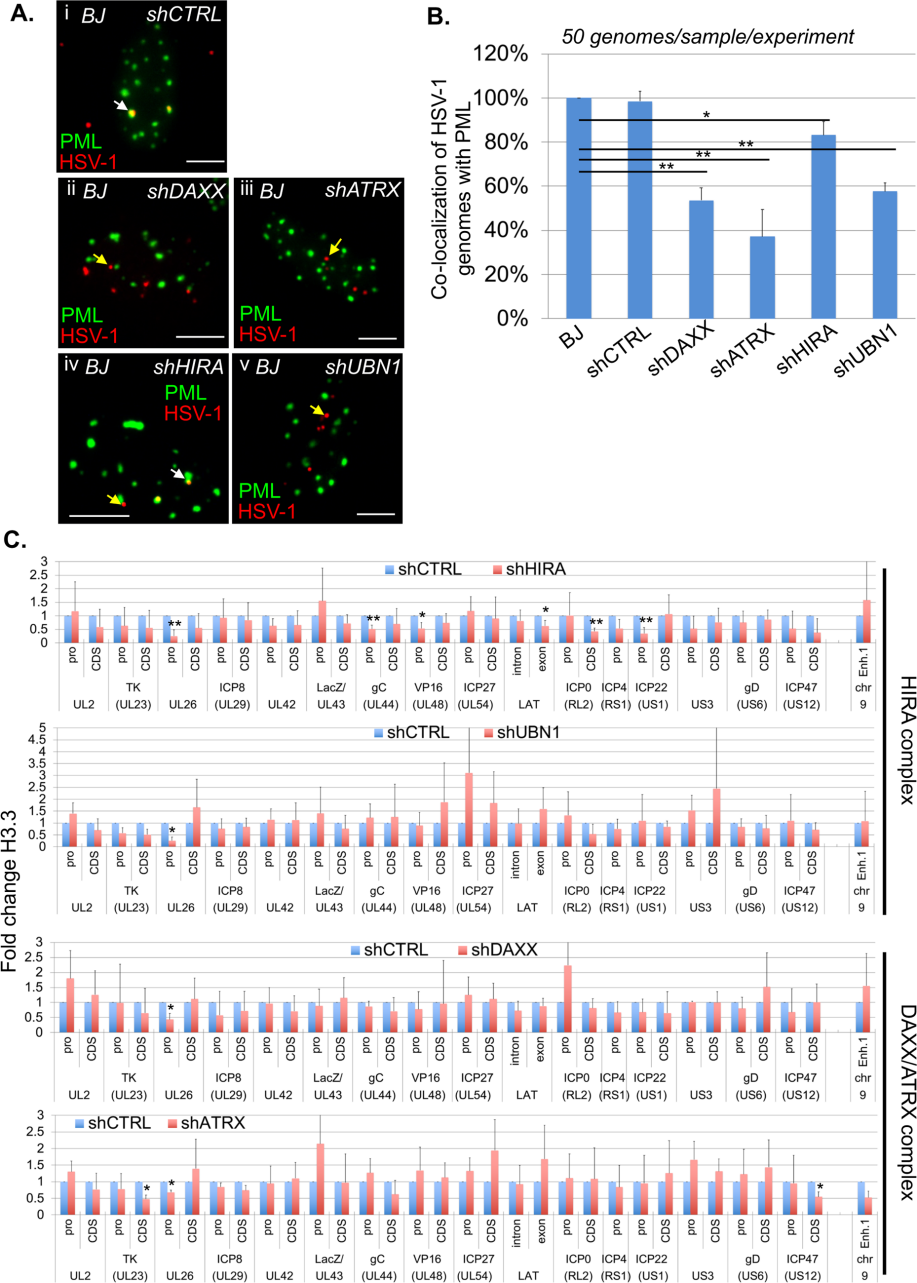
Individual inactivation of DAXX, ATRX, HIRA, or UBN1 significantly affects the formation of vDCP NBs, but only mildly affects the association of H3.3 with the latent/quiescent HSV-1 genome. Normal or e-H3.3-expressing BJ cells were first transduced with shRNA-expressing lentiviruses before *in*1374 infection. (A) Immuno-FISH performed in BJ cells infected with *in*1374 for 24 h. PML (green) and HSV-1 genomes (red) were detected in lentivirus-transduced BJ cells expressing control (shCTRL) or targeted shRNAs. Scale bars = 5 μm. (B) Quantifications of the immuno-FISH performed in (A). Means from three independent experiments ± SD. The Student’s *t*-test was applied to assess the significance of the results. * = p< 0.05, ** = p< 0.01 (see S4 Table for data). (C) ChIP for the detection of e-H3.3 associated with HSV-1 genomes and performed in e-H3.3-expressing BJ cells infected with *in*1374 for 24 h and previously transduced with a lentivirus expressing a control shRNA (shCTRL, blue) or a targeted shRNA (red). Anti-HA antibody was used for the ChIP experiments. The analyzed viral loci were described previously. Means from three independent experiments ± SD. The Student’s *t*-test was applied to assess the significance of the results. * = p< 0.05.

To analyze the requirement of the histone H3.3 chaperones for the formation of the vDCP NBs and HSV-1 chromatinization, DAXX, ATRX, HIRA or UBN1 were depleted by shRNAs in normal BJ cells or cells constitutively expressing e-H3.3 prior to infection with *in*1374 and completion of the experiments. The two tested shRNAs for each protein significantly diminished mRNA and protein quantities in BJ cells (S7A and S7B Fig). None of the shRNA impacted the detection of PML NBs, suggesting that PML NBs were potentially functional when the proteins were individually inactivated (S8 Fig). We first measured the impact of the depletion of each protein on the co-localization of HSV-1 genomes with PML. Both shRNAs for each protein gave similar results, i.e., a significant decrease in the co-localization between HSV-1 genomes and PML and thus a decrease in the formation and/or stability of the vDCP NBs (Fig 6A and 6B, S4 Table). These data show that the inactivation of any of the H3.3 chaperone complex affects to a certain extent the fate of vDCP NBs suggesting a connection between the activity of each H3.3 chaperone complex and the formation and/or maintenance of the vDCP NBs.

**Fig 7:**
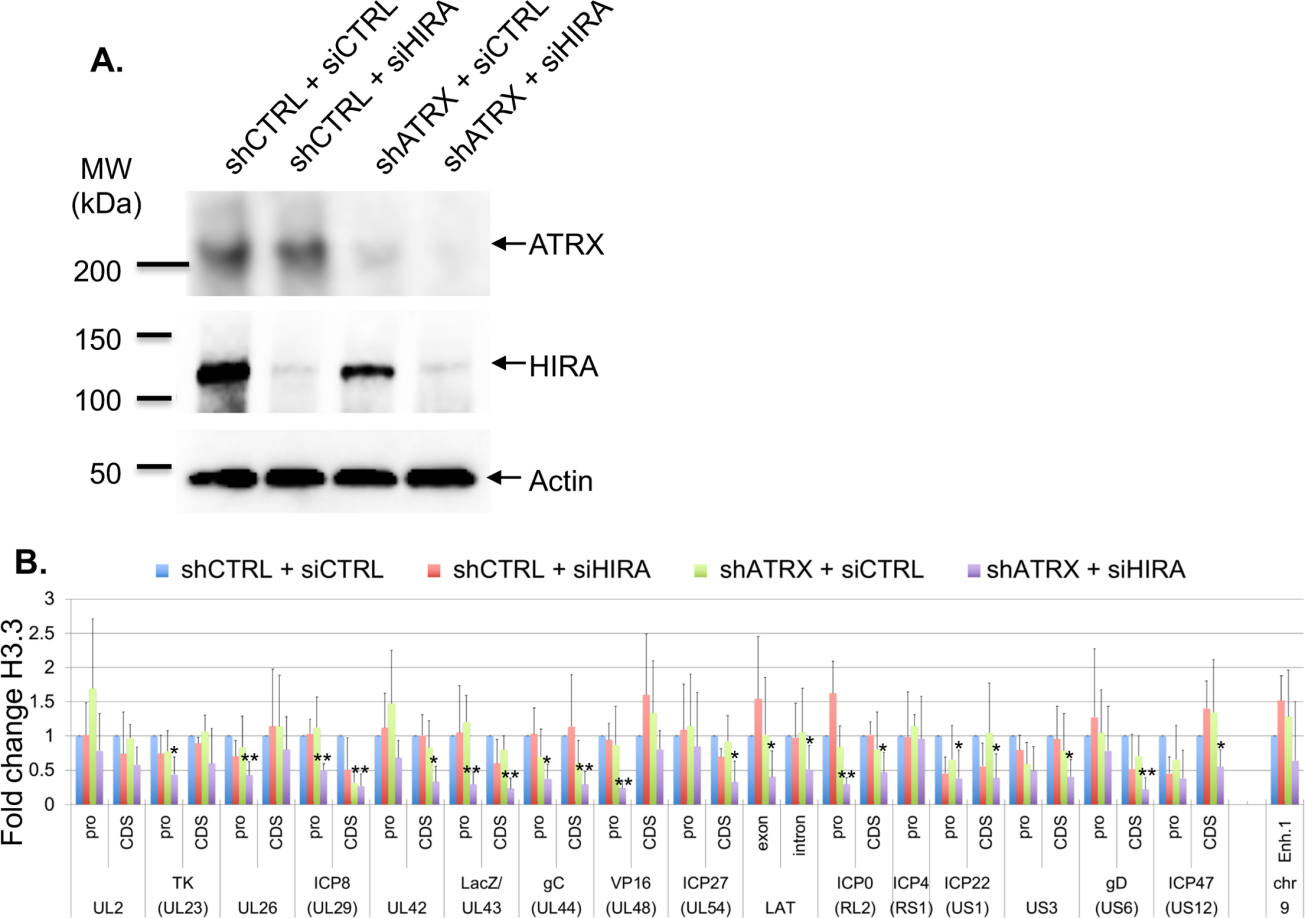
Functional inactivation of both DAXX/ATRX and HIRA complexes significantly affects the association of H3.3 with multiple loci of the latent/quiescent HSV-1 genome. (A) WB for the detection of ATRX and HIRA proteins in e-H3.3-expressing BJ cells. Cells were first transduced with a lentivirus expressing a control shRNA (shCTRL) or a shRNA against ATRX (shATRX) for 4 days. Then control siRNA (siCTRL) or HIRA siRNA (siHIRA) was transfected for 36 h. Actin was detected as a loading control. (B) ChIP for the detection of e-H3.3 associated with HSV-1 genomes and performed in e-H3.3-expressing BJ cells infected with *in*1374 for 24 h. Cells were processed with shRNAs-expressing lentiviruses then siRNAs similarly to (A), then infected with *in*1374 for 24 h. Anti-HA antibody was used for the ChIP experiments. The analyzed viral loci were described previously. Cellular loci Enhancer 1 (Enh.1) is a cellular control for deposition of H3.3. Means from three independent experiments ± SD. The Student’s *t*-test was applied to assess the significance of the results. * = p< 0.05, ** = p< 0.01.

We then analyzed the potential impact of the loss of vDCP NB stability on the H3.3-dependent HSV-1 chromatinization. We performed H3.3 ChIP in *in*1374-infected BJ e-H3.3 cells that had been previously depleted for HIRA, UBN1, DAXX or ATRX using one of the previously validated shRNAs (S9A and S9B Fig). The data showed that overall the inactivation of UBN1, DAXX or ATRX, had a weak impact on the association of H3.3 with the viral loci (1 to 3 loci significantly affected over 31, 3.2 to 9.6%) (Fig 6C). The depletion of HIRA had a relatively greater effect (6/31, 19.4%). To analyze if simultaneous inactivation of both complexes would significantly impact on HSV-1 chromatinization with H3.3, one protein of each complex was inactivated at the same time before performing HSV-1 infection (Fig 7A). Individual inactivation of HIRA and ATRX is known to lead to the functional inactivation of the HIRA and DAXX/ATRX complexes, respectively [35,52,63,64]. We noticed that the inactivation of HIRA by a siRNA was not as efficient as the shRNA on preventing the association of H3.3 with viral genomes (Fig 7B). This is likely due to differences in the efficiency of the siRNA compared to the shRNA (compare WBs of Figs S7B and 7A), and to the transitory effect of the siRNA compared to the stable effect of the shRNA at the time of the infection (see Materials and Methods). Nonetheless, a significant decrease of the association of H3.3 with a large number of viral loci (20/31, 64.5%) was measured by the simultaneous inactivation of HIRA and ATRX compared to their individual inactivation (Fig 7B). These results indicate that the DAXX/ATRX complex may compensate for the loss of the HIRA complex on the chromatinization of latent/quiescent HSV-1 genomes with H3.3, and conversely.

### PML NBs are essential for H3.3 chromatinization of latent/quiescent HSV-1 genomes

**Fig 8:**
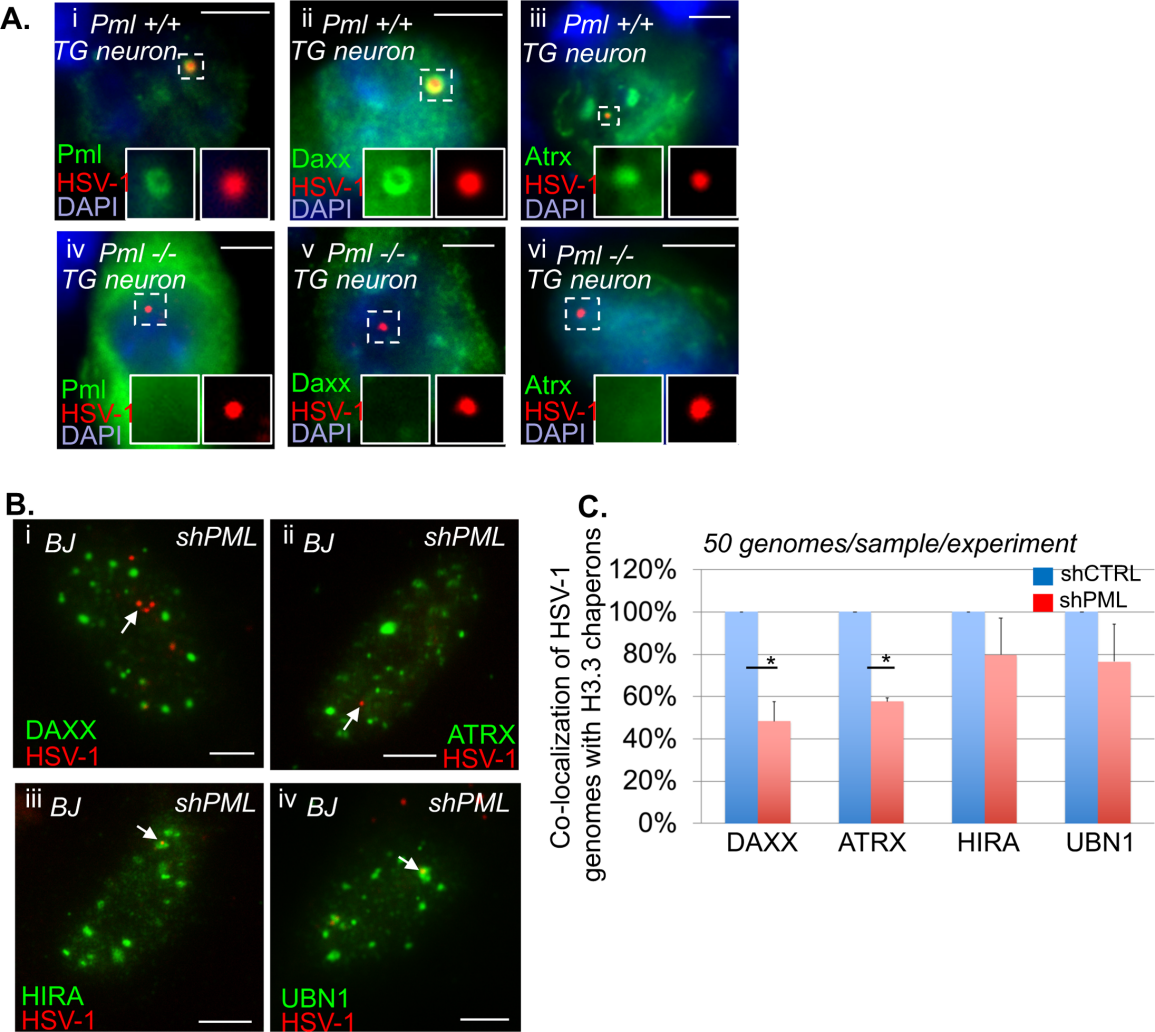
Absence of PML decreases the co-localization of DAXX and ATRX but not HIRA and UBN1 with latent/quiescent HSV-1 genomes. (A) Immuno-FISH performed in TG tissues from *pml*^+/+^ and *pml*^-/-^ infected mice at 28 dpi. Pml, Daxx, Atrx (green), and HSV-1 genomes (red) were detected. Nuclei were detected with DAPI (blue). Scale bars = 10 μm. (B) Immuno-FISH performed in BJ cells depleted of PML by transduction with a PML-targeted shRNA-expressing lentivirus, and subsequently infected with *in*1374 for 2 days. DAXX, ATRX, HIRA or UBN1 (green), and HSV-1 genomes (red) were detected. Scale bars = 5 μm. (C) Quantification of the immuno-FISH performed in (B). Means from three independent experiments ± SD. The Student’s *t*-test was applied to assess the significance of the results. * = p< 0.05 (See S5 Table for data).

The above experiments were conducted in a context where the cells, although deficient for the activity of one H3.3 chaperone complex at a time, still contained intact PML NBs accumulating e-H3.3 (S7 and S10 Fig). Therefore, we hypothesized that the accumulation of H3.3 within the PML NBs could be one of the key events acting upstream of the H3.3 chaperone complex activity for the induction of chromatinization of the latent/quiescent HSV-1 by H3.3. We analyzed the HSV-1 chromatinization in cells lacking PML NBs. In a previous study conducted in HSV-1 latently infected PML KO mice, we showed that the absence of PML significantly impacted the number of latently infected TG neurons showing the “single”/vDCP NB HSV-1 pattern and favored the detection of neurons containing the “multiple-latency” pattern prone to LAT expression [16,53]. We analyzed the very few neurons showing a “single”/vDCP NB-like pattern in the latently infected PML KO mice for the co-localization of DAXX and ATRX with the viral genomes. We could not detect any of the two proteins co-localizing with the latent HSV-1 genomes (Fig 8Ai to 8vi). Although informative, these *in vivo* studies did not allow the analysis of the real impact of the absence of PML on the co-localization of the other PML NB-associated proteins with latent HSV-1 genomes, because the neurons showing the “single”/vDCP NB-like pattern were too few to quantify the effect. We thus depleted PML in normal BJ cells using a PML shRNA-expressing lentiviral transduction approach. We verified the efficiency of the shRNAs against PML in normal BJ cells by IF, RT-qPCR and WB (S11A–S11C Fig). PML-depleted BJ cells were superinfected with *in*1374, and immuno-FISH was performed at 2 dpi to analyze the co-localization of HSV-1 genomes with DAXX, ATRX, HIRA, and UBN1 (Fig 8B). Notably, both PML shRNAs gave similar results. The quantification of the data showed that, similarly to the *in vivo* situation, the depletion of PML significantly decreased the co-localization of DAXX and ATRX with latent/quiescent HSV-1 genomes, leaving HIRA and UBN1 unaffected for their co-localization (Fig 8C, and S5 Table). Thus, we analyzed whether the failure of DAXX/ATRX to co-localize with the latent/quiescent HSV-1 genomes in the absence of PML NBs, could impact the chromatinization of HSV-1 with H3.3.

**Fig 9:**
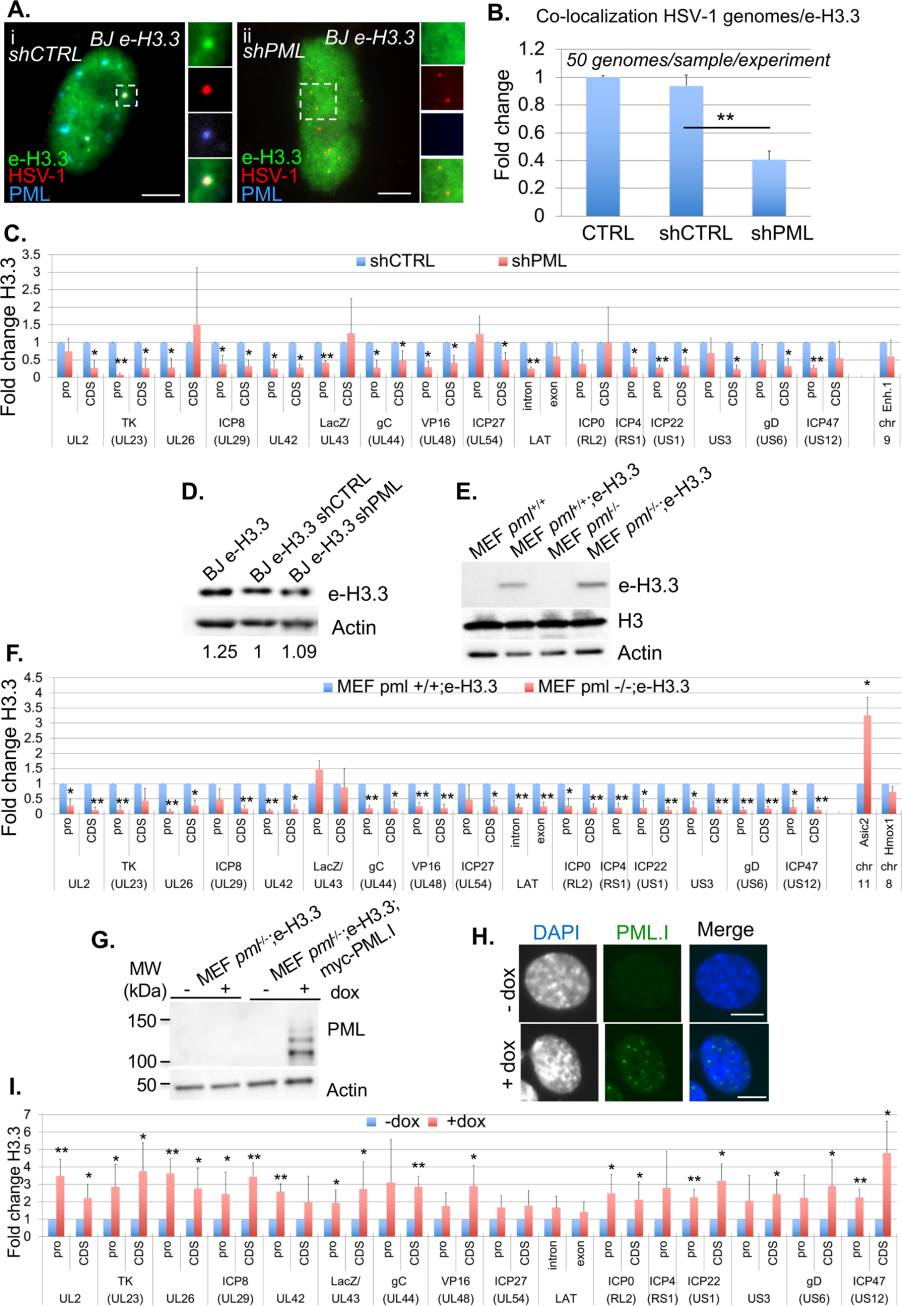
Depletion of PML significantly impacts the association of H3.3 with latent/quiescent HSV-1 genomes. (A) Immuno-FISH performed in e-H3.3-expressing BJ cells transduced with a control (shCTRL) or PML (shPML) shRNA-expressing lentivirus and subsequently infected with *in*1374 for 2 days. E-H3.3 (green), HSV-1 genomes (red), and PML (blue) were detected. Scale bars = 5 μm. (B) Quantification of the immuno-FISH performed in (A). Means from three independent experiments ± SD. The Student’s *t*-test was applied to assess the significance of the results. ** = p< 0.01. (C) ChIP for the detection of e-H3.3 associated with HSV-1 genomes and performed in e-H3.3-expressing BJ cells infected with *in*1374 for 24 h and previously transduced with a lentivirus expressing a control shRNA (shCTRL, blue) or a PML shRNA (shPML, red). Anti-HA antibody was used for the ChIP experiments. The analyzed viral loci were described previously. Means from three independent experiments ± SD. The Student’s *t*-test was applied to assess the significance of the results. * = p< 0.05 (D) WB for the detection of e-H3.3 in control e-H3.3-expressing BJ cells (CTRL) or e-H3.3-expressing BJ cells transduced with a lentivirus expressing a control shRNA (shCTRL) or a PML shRNA (shPML). Actin was detected as a loading control. (E) WB for the detection of e-H3.3 in control and e-H3.3-expressing MEF *pml*^+/+^ or *pml*^-/-^ cells. Actin and total histone H3 were detected as loading controls. (F) ChIP for the detection of e-H3.3 associated with HSV-1 genomes, and performed in e-H3.3-expressing *pml*^+/+^ (blue) or *pml*^-/-^ (red) MEF cells infected with *in*1374 for 24 h. Anti-HA antibody was used for the ChIP experiments. Analyzed viral loci were described previously. Cellular loci acid Sensing Ion Channel Subunit 2 (Asic2) and Heme Oxygenase 1 (Hmox1) are positive and negative controls for deposition of H3.3 in the absence of Pml, respectively (see *[44]*). Means from three independent experiments ± SD. The Student’s *t*-test was applied to assess the significance of the results. * = p< 0.05, ** = p< 0.01. (G) WB for the detection of ectopically-expressed human PML in MEF *pml*^-/-^;e-H3.3 or MEF *pml*^-/-^;e-H3.3;myc-PML.I cells not treated (-) or treated (+) with doxycycline for 24 h. Anti-human PML was used for the detection of PML. Actin was detected as a loading control. (H) IF for the detection of PML NBs (green) and nuclei (gray/blue) in MEF *pml*^-/-^;e-H3.3;myc-PML.I cells not treated (- dox) or treated (+ dox) with doxycycline for 24 h. PML was detected using the anti myc antibody. Scale bars = 5 μm. (I) ChIP for the detection of e-H3.3 associated with HSV-1 genomes and performed in MEF pml^-/-^;e-H3.3;myc-PML.I cells not treated (- dox) or treated (+ dox) with doxycycline for 24 h then infected with in1374 for 24 h in the presence of dox. Anti-HA antibody was used for the ChIP experiments. Analyzed viral loci were described previously. Means from three independent experiments ± SD. Student’s t-test was applied to assess the significance of the results. * = p< 0.05, ** = p < 0.01.

We first generated BJ e-H3.3 cells depleted for PML by shRNA-expressing lentiviral transduction similarly to the BJ cells (S11D and S11E Fig). BJ e-H3.3 control or PML-depleted cells were superinfected with *in*1374 to perform immuno-FISH and analyze the co-localization of HSV-1 genomes with H3.3 (Fig 9A). Quantification of the data showed a significant decrease in the co-localization of latent/quiescent HSV-1 genomes with H3.3 compared with controls (Fig 9B), suggesting an impact of the absence of PML NBs on the latent/quiescent HSV-1 association with H3.3. To complement these results at a more quantitative level, we performed ChIP on e-H3.3. The data showed a major impact of the absence of PML NBs on the H3.3 association with viral genomes, with a significant depletion of H3.3 on multiple loci (21/31, 68%) (Fig 9C). This could not be due to an indirect effect of PML depletion on H3.3 stability because e-H3.3 protein levels were similar in control cells and cells depleted for PML (Fig 9D). Both PML shRNAs gave similar results. To confirm that the absence of PML had an impact on the H3.3 association with latent/quiescent viral genomes, we performed ChIP on *in*1374-infected control MEF *pml*^+/+^ or MEF *pml*^-/-^ cells previously engineered by lentiviral transduction to express e-H3.3 (Fig 9E). The data confirmed the impaired association of e-H3.3 with latent/quiescent HSV-1 genomes in the absence of PML, with 26/31 (84%) viral loci significantly impacted (Fig 9F). Cellular loci acid Sensing Ion Channel Subunit 2 (Asic2), and Heme Oxygenase 1 (Hmox1) were used respectively as positive and negative controls for deposition of H3.3 in the absence of Pml as described in [44]. To definitively attribute the lack of deposition of H3.3 on viral loci to the absence of PML, MEF *pml*^-/-^;e-H3.3 cells were engineered to allow re-expression, under doxycycline induction, of the isoform I of human PML (PML.I) (Fig9G), which was shown to participate to the HSV-1 antiviral restriction mechanism [65]. The formation of PML NBs after induction of PML.I was visualized by IF (Fig 9H). ChIPs were then performed on *in*1374-infected MEF *pml*^-/-^;e-H3.3;myc-PML.I cells previously treated or not with doxycycline (Fig 9I). The data showed that the re-expression of PML.I allowed the re-loading of H3.3 on all the analyzed loci of the latent/quiescent viral genomes with significant results obtained for 21 loci over 31 (68%), demonstrating the essential role of PML/PML NBs in the association of H3.3 with incoming viral genomes. Finally, we wanted to analyze whether the deficit of the H3.3 association with the viral genome in the absence of PML could be compensated by an increase of H3.1 on viral loci. The data from BJ e-H3.1 cells depleted for PML or MEF *pml*^-/-^;e-H3.1 cells, and infected with *in*1374 showed that H3.1 did not replace H3.3 on the viral loci (S12A and S12B Fig). Altogether, these data demonstrate the essential role of PML NBs, probably through the DAXX/ATRX complex activity, in the exclusive H3.3 chromatinization of incoming viral genomes forced to adopt a vDCP NB-associated latent/quiescent pattern due to a deficit in the onset of lytic cycle.

### The destabilization of vDCP NBs induces the recovery of HSV-1 transcriptional activity and the formation of replication compartments

**Fig 10:**
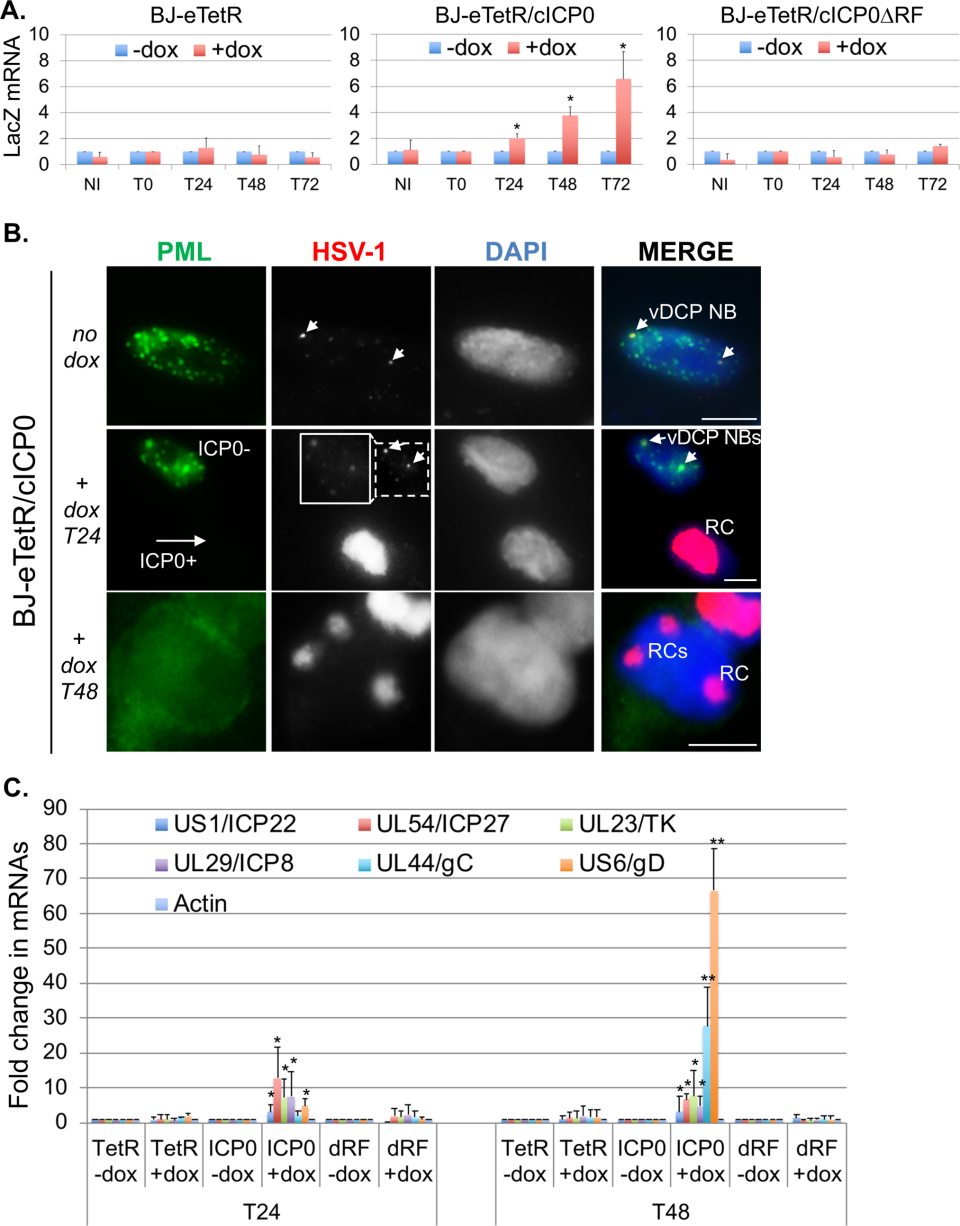
The destabilization of vDCP NBs induces the transcriptional recovery, and replication of vDCP NB-associated latent/quiescent HSV-1 genomes. (A) RT-qPCR performed in BJ-eTetR, BJ-eTetR/cICP0 or BJ-eTetR/cICP0*Δ*RF cells infected with *in*1374 for 4 d at 38.5°C, then treated (red) or not (blue) with doxycycline for 0 h (T0), 24 h (T24), 48 h (T48), or 72h (T72) at the permissive temperature (32°C) for *in*1374 replication. Transcription of the LacZ reporter gene was analyzed. Means from three independent experiments ± SD. Student’s *t*-test was applied to assess the significance of the results. * = p< 0.05. (B) Immuno-FISH performed in BJ-eTetR/cICP0 cells infected with *in*1374 for 4 d at 38.5°C, then treated or not with doxycycline for 24 h (T24), or 48 h (T48) at 32°C. PML (green), HSV-1 genomes (gray/red), and nuclei (DAPI, gray/blue) are detected. ICP0- and ICP0+ are detectable by the presence or not of PML NBs, respectively. The HSV-1 signal of the ICP0- cell in the + dox T24 is shown with two different set up to highlight the vDCP NBs-associated HSV-1 genomes compared to the cell showing the replication compartment (RC). Scale bars = 5 μm. (C) RT-qPCR performed in BJ-eTetR (TetR), BJ-eTetR/cICP0 (ICP0) or BJ-eTetR/cICP0*Δ*RF (dRF) cells infected with *in*1374 for 4 d at 38.5°C, then treated (+dox) or not (-dox) with doxycycline for 24 h (T24), or 48 h (T48), at the permissive temperature (32°C) for *in*1374 replication. Transcription of viral genes of different kinetics US1/ICP22, UL54/ICP27 (IE/α); UL23/TK, UL29/ICP8 (E/β); UL44/gC, US6/gD (L/γ) was analyzed. Actin gene was analyzed as a control. Means from three independent experiments ± SD. Student’s *t*-test was applied to assess the significance of the results. * = p< 0.05, ** = p < 0.01.

vDCP NB-associated latent genomes have been shown to be transcriptionally silent for the LAT expression *in vivo* [16], and for the expression of a reporter gene *in vitro* in mouse TG neuron cultures [17], and in human primary fibroblasts [45]. Moreover, it is known that the viral protein ICP0 induces the destabilization of PML NBs [66] and is essential for HSV-1 reactivation *in vivo* [67], and for the transcriptional de-repression of a silenced viral genome *in vitro* [45,59,60]. However, it is not known if the transcriptional recovery is correlated to the destabilization of the vDCP NBs. We analyzed if latent HSV-1 genomes trapped in vDCP NBs were definitively silenced or could resume a transcriptional program leading to replication of viral genomes provided that vDCP NBs were destabilized. ICP0 or its non-functional RING finger mutant (ICP0ΔRF) were expressed from BJ-eTetR/cICP0 or BJ-eTetR/cICP0ΔRF cells harboring vDCP NBs for 4 days (Fig 10). HSV-1 *in*1374 infected BJ-eTetR cells were used as controls. Expression of ICP0 or ICP0ΔRF was induced for 24 h, 48 h or 72h at the permissive temperature for *in*1374 replication (32°C) (S13 Fig). Transcription of the reporter (LacZ) gene was measured by RT-qPCR to analyze the transcriptional recovery of the vDCP NB-associated latent/quiescent viral genomes (Fig 10A). The addition of doxycycline in infected BJ-eTetR or BJ-eTetR/c ICP0ΔRF cells did not lead to any significant transcription of the LacZ gene. Only infected BJ-eTetR/cICP0 showed the recovery of LacZ mRNA transcription from 24 h post addition of doxycycline. To analyze if the virus could sustain replication, as suggested by the observation of the BJ-eTetR/cICP0 cell monolayer (S14 Fig), following vDCP NB destabilization, immuno-FISH were performed at 24 h and 48 h post-addition of doxycycline. BJ-eTetR/cICP0 cells (Fig 10B) but not BJ-eTetR/cICP0ΔRF (S15 Fig) showed a clear disappearance of the vDCP NBs. Concomitantly, only BJ-eTetR/cICP0 cells showed the formation of replication compartments (RCs) indicating that the virus is in the process of lytic phase following vDCP NBs destruction by ICP0. To confirm that the lytic transcriptional program was indeed occurring, viral transcripts of all kinetics were analyzed (Fig 10C). Twenty four and 48 h post ICP0 induction, lytic genes were expressed with a clear switch towards the γ genes (UL44/gC and US6/gD) at 48 h confirming the onset of the lytic transcriptional program. Expression of ICP0ΔRF did not enable the re-expression of viral genes. These data show that vDCP NBs-associated latent/quiescent HSV-1 genomes can resume transcription and a lytic program provided that the vDCP NBs are destabilized, suggesting that these genomes are not definitively silenced, and could participate to the reactivation process of HSV-1.

## Discussion

The HSV-1 genome enters the nucleus of infected neurons, which support HSV-1 latency as a naked/non-nucleosomal DNA. Many studies have described the acquisition of chromatin marks on the viral genome concomitantly to the establishment, and during the whole process, of latency. Paradoxically, although it is undisputable that these chromatin marks will predominantly be associated with latency and reactivation, few data are available for the initiation of the chromatinization of the incoming viral genome. Here, we demonstrate the essential contribution of PML NBs in the process of chromatinization of incoming HSV-1 genomes meant to remain in a latent/quiescent state. We showed that PML NBs are essential for the association of the histone variant H3.3 with the latent/quiescent HSV-1.

Two members of the HIRA complex, HIRA and ASF1a, were previously shown to be involved in H3.3-dependent chromatinization of HSV-1 genomes at early times after infection in non-neuronal and non-primary cells favoring the onset of the lytic cycle [68,69]. Moreover a recent study highlighted the interaction of HIRA with quiescent HSV-1 and plasmid DNA in primary human fibroblasts [56]. Our *in vivo* data in TG neurons and *in vitro* data in infected human primary fibroblasts or adult mice TG neuron cultures, show that all the proteins of the HIRA complex accumulate within specific nucleoprotein structures called the viral DNA-containing PML NBs or vDCP NBs. vDCP NBs contain transcriptionally silent HSV-1 genome that we previously demonstrated *in vivo* to be associated with the establishment of latency from the early steps of neuron infection [17]. Additionally, our data show that: (i) the mouse Hira protein, *in vivo*, and all the components of the HIRA complex, in cultured cells, temporarily accumulate in vDCP NBs, and (ii) significantly greater amount of incoming HSV-1 genomes co-localize with HIRA compared with PML at very early times pi (30 min). These data suggest that the HIRA complex could also be involved to some extent in the establishment of HSV-1 latency by the initial recognition of the incoming naked/non-nucleosomal viral DNA and the chromatinization of non-replicative HSV-1 genomes intended to become latent. In this respect, a recent study suggested an anti-viral activity associated with HIRA against HSV-1 and murine cytomegalovirus lytic cycles [56]. To that extent, although they are both functionally essential for the activity of the HIRA complex [35,51,64,70], our data show that the depletion of HIRA has a greater effect compared to the UBN1 depletion, on the H3.3 association with the viral genomes. This could be simply explained by a better efficiency of the HIRA, compared to the UBN1, shRNAs. Alternatively, HIRA was shown to be recruited to UV-induced DNA damage independently of UBN1 (see figure S2D in [71]), and to participate to the loading of newly synthesized H3.3 on chromatin [72]. Therefore, the depletion of HIRA could indirectly and/or directly impact on two initial events occurring concomitantly to the entry of the viral genomes in the nucleus; first a signaling pathway associated to the detection of DNA breaks present in incoming viral DNA as suggested in [55,73]; and second the chromatinization process *per se*. If these two events are linked it could explain the differences observed between the HIRA and UBN1 depletion on the loading of H3.3 on the viral genomes. Experiments are in progress to investigate this.

Interestingly, proteins of the HIRA complex have been previously shown to be able to directly bind to naked DNA in a sequence-independent manner, in contrast to DAXX and ATRX [52]. Nevertheless, our ChIP data highlight the interaction of viral genomes with DAXX and ATRX, but we cannot assert that the two proteins directly interact with naked DNA. The gamma-interferon-inducible protein 16 (IFI16), a member of the PYHIN protein family, has been described as a nuclear sensor of incoming herpesviruses genomes, and suggested to promote the addition of specific chromatin marks that contribute to viral genome silencing [74–81]. A proteomic study determining the functional interactome of human PYHIN proteins revealed the possible interaction between ATRX and IFI16 [82]. Thus, it will be interesting to determine in future studies if IFI16 and H3.3 chaperone complexes physically and functionally cooperate in the process of chromatinization of the latent/quiescent HSV-1 genome.

One of the main finding of our study is the demonstration of the essential contribution of PML NBs in the H3.3-dependent chromatinization of the latent/quiescent HSV-1 genomes. A close link between PML NBs and H3.3 in chromatin dynamics has been demonstrated during oncogene-induced senescence (OIS). In OIS, expression of the oncogene H-RasV12 induces DAXX-dependent relocalization of neo-synthesized H3.3 in the PML NBs before a drastic reorganization of the chromatin to form senescence-associated heterochromatin foci [42,43]. Hence, the contribution of the PML NBs in the deposition of H3.3 on specific cellular chromatin loci has also been reported [43,44]. The present study shows that the absence of Pml in HSV-1wt latently infected Pml KO mice, or the depletion of PML by shRNA in BJ cells infected with *in*1374, significantly affects the co-localization of DAXX and ATRX, but not HIRA and UBN1, with latent/quiescent HSV-1 genomes, confirming previous studies for DAXX and ATRX [45]. Taken together with the impaired association of H3.3 with the viral genomes in the absence of PML NBs, these data suggest that a significant part of the latent/quiescent HSV-1 genome chromatinization by H3.3 could occur through the activity of the DAXX/ATRX complex in association with the PML NBs.

Given the particular structure formed by the latent/quiescent HSV-1 genome with the PML NBs, our study raises the question of the possible acquisition of a chromatin structure within the vDCP NBs. The individual inactivation of DAXX, ATRX, HIRA, or UBN1 significantly impacts the co-localization of the latent/quiescent HSV-1 genomes with PML, and hence the formation of vDCP NBs. However, it only mildly affects the association of H3.3 with viral genomes, suggesting an absence of correlation between the formation of vDCP NBs and H3.3 chromatinization. However, our data show that the depletion of DAXX, ATRX, HIRA, or UBN1 does not modify the accumulation of e-H3.3 at PML NBs, leaving intact the upstream requirement of H3.3 accumulation in PML NBs for H3.3-dependent viral chromatin assembly. We have recently shown that vDCP NBs are dynamic structures that can fuse during the course of a latent infection [17]. It is thus possible that incoming viral genomes can be dynamically associated with vDCP NBs to be chromatinized, and in the absence of any of the H3.3 chaperone complex subunit, this dynamic can be perturbed, resulting in some viral genomes that do not show a co-localization with PML. Given that depletion of none of the four proteins affects the structure of the PML NBs, and considering the essential role of PML NBs in the H3.3 chromatinization of the viral genomes, this possibility cannot be ruled out. The depletion of H3.3, which almost exclusively participates in latent/quiescent HSV-1 genome chromatinization compared to H3.1/2, does not prevent the formation of vDCP NBs (S16 Fig), and is rather in favor of a chromatinization of the viral genome in the vDCP NBs. It is unlikely that canonical H3.1/2 could replace H3.3 for the chromatinization of the incoming HSV-1 genomes prior to the formation of the vDCP NBs. Indeed, our multiple immuno-FISH and ChIP assays failed to detect H3.1/2 and/or H3.1/2 chaperones that associate or co-localize with viral genomes. Nonetheless, we cannot rule out a possible replacement of H3.3 with another H3 variant for the chromatinization of viral genomes before their entrapment by the PML NBs to form vDCP NBs.

Our data show that the vDCP NBs-associated HSV-1 genomes are chromatinized with H3K9me3, and the Re-ChIP assays confirm an association with H3.3K9me3, but not H3K27me3. *In vivo*, it has been shown that both H3 modifications could be found on latent HSV-1 genomes [31,33,34]. One simple explanation could reside in the heterogeneity of latent genomes distribution within the nuclei of the infected neurons in the *in vivo* mouse and/or rabbit models of latency [16,17,54], however this would need to be formally demonstrated. Though, vDCP NBs-associated HSV-1 genomes remain compatible with the transcription of lytic genes provided that the vDCP NBs are destabilized by ICP0, a viral protein known to be required for full *in vivo* reactivation [67], and to erase chromatin marks associated with latent/quiescent viral genomes *in vitro* [59]. Therefore, vDCP NBs are not a dead end for the virus life cycle, and HSV-1 latently infected neurons containing vDCP NBs are likely to contribute to the process of reactivation.

Altogether, our study demonstrates the essential role of a PML NB/H3.3/H3.3 chaperone axis in the process of chromatinization of viral genomes adopting a vDCP NB pattern, which represents an essential structural and functional aspect of HSV-1 latency establishment. Given the involvement of H3.3 in the chromatinization of other latent herpesviruses belonging to different sub-families than HSV-1, such as EBV [83] and HCMV [58], as well as adenovirus type 5 [84], this pathway of chromatinization is likely to play a major role in the biology of the whole *Herpesviridae* family, and possibly of other DNA viruses such as adenoviruses, papillomaviruses, hepatitis B virus, and retroviruses.

## Materials and methods

### Ethics statement

All procedures involving experimental animals conformed to the ethical standards of the Association for Research in Vision and Ophthalmology (ARVO) statement for the use of animals in research and were approved by the local Ethics Committee of the Institute for Integrative Biology of the Cell (I2BC) and the Ethics Committee for Animal Experimentation (CEEA) 59 (Paris I) under number 2012–0047 and in accordance with European Community Council Directive 2010/63/EU. For animal experiments performed in the USA: animals were housed in American Association for Laboratory Animal Care-approved housing with unlimited access to food and water. All procedures involving animals were approved by the Children’s Hospital Animal Care and Use Committee and were in compliance with the Guide for the Care and Use of Laboratory Animals (protocol number: IAUC2013-0162 of 2/28/2107).

### Virus strains, mice and virus inoculation, primary mouse TG neuron cultures, cells

The HSV-1 SC16 strain was used for mouse infections and has been characterized previously [85]. The HSV-1 mutant *in*1374 is derived from the 17 *syn* + strain and expresses a temperature-sensitive variant of the major viral transcriptional activator ICP4 [86] and is derived from *in*1312, a virus derived from the VP16 insertion mutant *in*1814 [87], which also carries a deletion/frameshift mutation in the ICP0 open reading frame [88] and contains an HCMV-*lacZ* reporter cassette inserted into the UL43 gene of *in*1312 [89]. This virus has been used and described previously [17,45]. All HSV-1 strains were grown in baby hamster kidney cell (BHK-21, ATCC, CCL-10) and titrated in human bone osteosarcoma epithelial cells (U2OS, ATCC, HTB-96). *In*1374 was grown and titrated at 32°C in the presence of 3 mM hexamethylene bisacetamide [90].

PML wild-type, and knockout mice were obtained from the NCI Mouse Repository (NIH, http://mouse.ncifcrf.gov; strain, 129/Sv-*Pml*^*tm1Ppp*^) [91]. Genotypes were confirmed by PCR, according to the NCI Mouse Repository guidelines with primers described in [16].

Mice were inoculated and TG processed as described previously [16]. Briefly, for the lip model: 6-week-old inbred female BALB/c mice (Janvier Labs, France) were inoculated with 10^6^ PFU of SC16 virus into the upper-left lip. Mice were sacrificed at 6 or 28 dpi. Frozen sections of mouse TG were prepared as described previously [16,92]. For the eye model: inoculation was performed as described previously [93]. Briefly, prior to inoculation, mice were anesthetized by intra-peritoneal injection of sodium pentobarbital (50 mg/kg of body weight). A 10-μL drop of inoculum containing 10^5^ PFU of 17syn+ was placed onto each scarified corneal surface. This procedure results in ~80% mouse survival and 100% infected TG.

Primary mouse TG neuron cultures were established from OF1 male mice (Janvier lab), following a previously described procedure [17]. Briefly, 6–8-week-old mice were sacrificed before TG removal. TG were incubated at 37°C for 20 min in papain (25 mg) (Worthington) reconstituted with 5 mL Neurobasal A medium (Invitrogen) and for 20 min in Hank’s balanced salt solution (HBSS) containing dispase (4.67 mg/mL) and collagenase (4 mg/mL) (Sigma) on a rotator, and mechanically dissociated. The cell suspension was layered twice on a five-step OptiPrep (Sigma) gradient, followed by centrifugation for 20 min at 800 *g*. The lower ends of the centrifuged gradient were transferred to a new tube and washed twice with Neurobasal A medium supplemented with 2% B27 supplement (Invitrogen) and 1% penicillin–streptomycin (PS). Cells were counted and plated on poly-D-lysine (Sigma)- and laminin (Sigma)-coated, eight-well chamber slides (Millipore) at a density of 8,000 cells per well. Neuronal cultures were maintained in complete neuronal medium consisting of Neurobasal A medium supplemented with 2% B27 supplement, 1% PS, L-glutamine (500 μM), nerve growth factor (NGF; 50 ng/mL, Invitrogen), glial cell-derived neurotrophic factor (GDNF; 50 ng/mL, PeproTech), and the mitotic inhibitors fluorodeoxyuridine (40 μM, Sigma) and aphidicolin (16.6 μg/mL, Sigma) for the first 3 days. The medium was then replaced with fresh medium without fluorodeoxyuridine and aphidicolin.

Primary human foreskin (BJ, ATCC, CRL-2522), lung (IMR-90, Sigma, 85020204), fetal foreskin (HFFF-2, European Collection of Authenticated Cell Cultures, ECACC 86031405, kind gift from Roger Everett, CVR-University of Glasgow) fibroblast cells, primary human hepatocyte (HepaRG, HPR101, kind gift from Olivier Hantz & Isabelle Chemin, CRCL, Lyon, France) cells, human embryonic kidney (HEK 293T, ATCC CRL-3216, kind gift from M. Stucki, University Hospital Zürich) cells, U2OS, mouse embryonic fibroblast (MEF) *pml*^+/+^, MEF *pml*^-/-^ cells (kind gift from Valérie Lallemand, Hopital St Louis, Paris), and BHK-21 cells were grown in Dulbecco’s Modified Eagle’s Medium (DMEM) supplemented with 10% fetal bovine serum (Sigma, F7524), L-glutamine (1% v/v), 10 U/mL penicillin, and 100 mg/mL streptomycin. BJ cell division is stopped by contact inhibition. Therefore, to limit their division, cells were seeded at confluence before being infected at a multiplicity of infection (m.o.i.) of 3, and then maintained in 2% serum throughout the experiment. Infections of BJ cells for short times (from 30 min to 6 h) were performed by synchronizing the infection process with a pre-step of virus attachment to the cells at 4°C for one hour. The infection medium was then removed, and the temperature was shifted to 37°C to allow a maximum of viruses to simultaneously penetrate into the cells.

### Frozen sections

Frozen sections of mouse TG were generated as previously described [92]. Mice were anesthetized at 6 or 28 d.p.i., and before tissue dissection, mice were perfused intracardially with a solution of 4% formaldehyde, 20% sucrose in 1X PBS. Individual TG were prepared as previously described [92], and 10-μm frontal sections were collected in three parallel series and stored at -80°C.

### DNA-FISH and immuno-DNA FISH

HSV-1 DNA FISH probes consisting of cosmids 14, 28 and 56 [94] comprising a total of ~90 kb of the HSV-1 genome were labeled by nick-translation (Invitrogen) with dCTP-Cy3 (GE Healthcare) and stored in 100% formamide (Sigma). The DNA-FISH and immuno-DNA FISH procedures have been described previously [16,92]. Briefly, infected cells or frozen sections were thawed, rehydrated in 1x PBS and permeabilized in 0.5% Triton X-100. Heat-based unmasking was performed in 100 mM citrate buffer, and sections were post-fixed using a standard methanol/acetic acid procedure and dried for 10 min at RT. DNA denaturation of the section and probe was performed for 5 min at 80°C, and hybridization was carried out overnight at 37°C. Sections were washed 3 x 10 min in 2 x SSC and for 3 x 10 min in 0.2 x SSC at 37°C, and nuclei were stained with Hoechst 33258 (Invitrogen). All sections were mounted under coverslips using Vectashield mounting medium (Vector Laboratories) and stored at 4°C until observation.

For immuno-DNA FISH, cells or frozen sections were treated as described for DNA-FISH up to the antigen-unmasking step. Tissues were then incubated for 24 h with the primary antibody. After three washes, secondary antibody was applied for 1 h. Following immunostaining, the cells were post-fixed in 1% PFA, and DNA FISH was carried out from the methanol/acetic acid step onward.

The same procedures were used for infected neuronal cultures except that the cells were fixed in 2% PFA before permeabilization.

### Western blotting

Cells were collected in lysis buffer (10 mM Tris-EDTA, pH 8.0) containing a protease inhibitor cocktail (Complete EDTA-free; Roche) and briefly sonicated. Protein extracts were homogenized using QiaShredders (Qiagen). The protein concentration was estimated by the Bradford method. Extracted proteins were analyzed by Western blotting using appropriate antibodies (see below).

### Microscopy, imaging, and quantification

Observations and most image collections were performed using an inverted Cell Observer microscope (Zeiss) with a Plan-Apochromat ×100 N.A. 1.4 objective and a CoolSnap HQ2 camera from Molecular Dynamics (Ropper Scientific) or a Zeiss LSM 800 confocal microscope. Raw images were processed using ImageJ software (NIH).

### Lentivirus and retrovirus production and establishment of cell lines

BJ or MEF cell lines expressing H3.1-SNAP-HAx3 (e-H3.1), H3.3-SNAP-HAx3 (e-H3.3), or Myc-hDAXX were established by retroviral transduction [95]. Briefly, pBABE plasmids encoding H3.1-SNAP-HAx3 or H3.3-SNAP-HAx3 (gift from Dr L. Jansen), pLNCX2 encoding Myc-hDAXX [43], were co-transfected with pCL-ampho (for subsequent transduction of BJ cells, kind gift from M. Stucki, University Hospital Zürich) or pCL-eco (for subsequent transduction of MEF cells, kind gift from M. Stucki, University Hospital Zürich) plasmids [96] by the calcium phosphate method into HEK 293T cells to package retroviral particles [97]. BJ cells stably expressing HIRA-HA and HA-UBN1 or transiently expressing the shRNAs were established by lentiviral transduction. Briefly, pLenti encoding HIRA-HA or HA-UBN1, pLKOneo.CMV.EGFPnlsTetR, pLKO.DCMV.TetO.cICP0, pLKO.DCMV.TetO.cICP0ΔRF (gift from Dr. R. D. Everett, [98]), pLVX-TetOne-Myc-PML.I (issued from pLNGY-PML.I, gift from Dr. R. D. Everett [65]), pLKO empty, pLKO shPML_01, 02, shDAXX_01, 02, shATRX_01, 02, shHIRA_01, 02, shUBN1_01, 02, were co-transfected with psPAX.2 (Addgene #12260) and pMD2.G (Addgene #12259) plasmids by the calcium phosphate method into HEK 293T cells to package lentiviral particles. After 48 h, supernatant containing replication-incompetent retroviruses or lentiviruses was filtered and applied for 24 h on the target BJ or MEF cells in a medium containing polybrene 8 μg/mL (Sigma) [95]. Stable transfectants were selected with Blasticidin S (5 μg/mL, Invivogen), puromycin (1 μg/mL, Invivogen), or neomycin (G418, 1 mg/mL, Millipore) for 3 days, and a polyclonal population of cells was used for all experiments. Target sequences of the shRNA-expressing plasmids are provided in Table 1.

**Table 1:** Characteristics of the shRNA-expressing plasmids.

Plasmids	Origin	Target sequences
pLKO1-puro shCTRL	Sigma SHC002 lot 01181209MN	CCGGCAACAAGATGAAGAGCACCAA
pLKO1-puro shPML_01	Sigma TRCN 0000003867 NM_002675 x-1497s1c1	GCCAGTGTACGCCTTCTCCAT
pLKO1-puro shPML_02	Sigma TRCN 0000003869 NM_002675 x-1501s1c1	GTGTACGCCTTCTCCATCAAA
pLKO1-puro shATRX_01	Sigma TRCN 0000013590 NM_00489 2-2215s1c1	CGACAGAAACTAACCCTGTAA
pLKO1-puro shATRX_02	Sigma TRCN 0000342811 NM_00489 3-2357s21c1	GATAATCCTAAGCCTAATAAA
pSuper.retro.puro-shDAXX_01	[99]	GGAGUUGGAUCUCUCAGAA
pLKO1-puro shDAXX_02	Sigma TRCN 0000003802 NM-001350 x-2285s1c1	GCCACACAATGCGATCCAGAA
pLKO1-puro shHIRA_01	Sigma TRCN 0000232156 NM_003325 3-592s21c1	CTCTATCCTCCGGAATCATTC
pLKO1-puro shHIRA_02	Sigma TRCN 0000232159 NM_003325 3-3073s21c1	TGAATACCGACTTCGAGAAAT
pLKO1-puro shUBN1_01	Sigma TRCN 0000235872 NM_016936 3-1764s21c1	ATGGACTCGCTGACGGATTTG
pLKO1-puro shUBN1_02	Sigma TRCN 0000235871 NM_016936 3-1244s21c1	ATCCGACTCCTTCATCGATAA

### ChIP and quantitative PCR

Cells were fixed with methanol-free formaldehyde (#28908, Thermo Fisher Scientific) 1% for 5 min at RT, and then glycine 125 mM was added to arrest fixation for 5 min. After two washes with ice-cold PBS, the cells were scraped and resuspended in “Lysis Buffer” (10% glycerol, 50mM HEPES pH7,5; 140mM NaCl; 0,8% NP40;0,25% Triton; 1mM EDTA, Protease Inhibitor Cocktail 1X (PIC) (Complete EDTA-free; Roche) and incubated for 10 min at 4°C under shaking. The cells were subsequently washed in “Wash buffer”(200mM NaCl; 20mM Tris pH8; 0,5mM EGTA; 1mM EDTA, PIC 1X) for 10 min at 4°C under shaking then were resuspended and centrifuged twice during 5 min 1700g at 4°C in “Shearing Buffer” (10mM Tris pH7,6; 1mM EDTA; 0,1%SDS; PIC 1X). Finally, nuclei were resuspended in 1mL of “Shearing Buffer” and were sonicated with a S220 Focused-ultrasonicator (Covaris) (Power 140W; Duty Off 10%; Burst Cycle 200). Eighty-five μL of the sonication product were kept for the input, 50 μL for analyzes of the sonication efficiency, and 850 μL diluted twice in IP buffer 2X (300mM NaCl, 10mM Tris pH8; 1mM EDTA; 0,1% SDS; 2% Triton) for ChIP. Two micrograms of Ab were added and incubated overnight at 4°C. Fifty microliters of agarose beads coupled to protein A (Millipore 16–157) or G (Millipore 16–201) were added for 2 h at 4°C under constant shaking. Beads were then successively washed for 5 min at 4°C under constant shaking once in “low salt” (0.1% SDS, 1% Triton X-100, 2 mM EDTA, 20 mM Tris HCl pH 8.0, 150 mM NaCl) buffer, once in “high salt” (0.1% SDS, 1% Triton X-100, 2 mM EDTA, 20 mM Tris HCl pH 8.0, 500 mM NaCl) buffer, once in “LiCl” (0.25 mM LiCL, 1% NP40, 1% NaDOC, 1 mM EDTA, 10 mM Tris HCl pH 8.0) buffer, and twice in TE (10 mM Tris pH 8.0, 1 mM EDTA) buffer. Chromatin-antibody complexes are then eluted at 65°C for 30 min under constant shaking with 200 μL of elution buffer (1% SDS, 0.1 M NaHCO3). Input and IP products were de-crosslinked overnight at 65°C with 20 mg/mL of proteinase K (Sigma) and 10 mg/mL of RNAse A (Sigma). DNA was then purified by phenol-chloroform/ethanol precipitation, resuspended in water, and kept at -20°C until use for qPCR.

Quantitative PCR was performed using Quantifast SYBR Green mix (Qiagen) and the MX3005P apparatus (Agilent/Stratagene). Primers were used at a final concentration of 1 μM. Their sequences and target genes are provided in Tables 2 and 3.

**Table 2:** Primer sequences and target HSV-1 genes.

Genes	Proteins		Forward primers (5’-> 3’)	Reverse primers (5’-> 3’)
UL2	Uracil DNA glycosylase	promoter	TAAACCAACGAAAAGCGCGG	5’ GGCACCCACAGAAACCTACA 3’
		CDS	CCCTCTCCAAGGTTCCGTTC	CGACCAGTCGATGGGTGAAA
UL23	TK	promoter	CAGCTGCTTCATCCCCGTGG	AGATCTGCGGCACGCTGTTG
		CDS	ATGCTGCCCATAAGGTATCG	GTAATGACAAGCGCCCAGAT
UL26	VP21	promoter	AGAACAAGAGCCTCCGTTGG	AGGCGAGAGCGAATGCTAAA
		CDS	CCCATTTACGTGGCTGGGTT	TCCGGATCCAATGCCAACTC
UL29	ICP8	promoter	CCTTTTGTCAATCGGTCCGC	CGGGAGACATACCTTGTCGG
		CDS	TGTTCACCACGAGTACCTGC	ACCTATGCACCTTCGACACG
UL42	DNA polymerase	promoter	CGTAGTTTCTGGCTCGGTGA	GAACACCCCACAGTGACGAG
		CDS	TGTTCACCACGAGTACCTGC	TTTCCCCGTACACCGTCTTG
LacZ/UL43 (HCMV-lacZ reporter cassette)	β-Galactosidase	Promoter (CMV)	TTCCTACTTGGCAGTACATCTACG	GTCAATGGGGTGGAGACTTGG
		CDS (LacZ)	GCAGCAACGAGACGTCA	GAAAGCTGGCTACAGGAAG
UL44	gC	promoter	CGCCGGTGTGTGATGATTT	TTTATACCCGGGCCCCAT
		CDS	GGGTCCGTCCCCCCCAAT	CGTTAGGTTGGGGGCGCT
UL48	VP16	promoter	GCGTTCATGTCGGCAAACAG	CCCGTATCAACCCCACCCAAT
		CDS	TGCGGGAGCTAAACCACATT	TCCAACTTCGCCCGAATCAA
UL54	ICP27	promoter	CCACGGGTATAAGGACATCCA	GGATATGGCCTCTGGTGGTG
		CDS	GGCGACTGACATTGA	CTGCTGTCCGATTCCAGGTC
LAT	Latency Associated transcript	exon	GGCTCCATCGCCTTTCCT	AAGGGAGGGAGGAGGGTACTG
		intron	CCCACGTACTCCAAGAAGGC	AGACCCAAGCATAGAGAGCCAG
RL2	ICP0	promoter	CCGCCGACGCAACAG	CCGCCGACGCAACAG
		CDS	CGTGTGCACGGATGAGATCG	GCGCAATTGCATCCAGGTTT
RS1	ICP4	promoter	CGTGGTGGTGCTGTACTCG	GCTCGGCGGACCACTC
US1	ICP22	promoter	GATCGCATCGGAAAGGGACA	GGTGCTTACCCGTGCAAAAA
		CDS	GTTACGCTGGAAACCCCAGA	CCAGACACTTGCGGTCTTCT
US3	Protein kinase US3	promoter	GCGGGGGCTGCTCTAAAAAT	GGGTTTTAAGGAGCGGCAGT
		CDS	ACTGGCATGGGCTTTACGAT	TTCACGATTACCCGTTGGGG
US6	gD	promoter	GGGGTTAGGGAGTTGTTCGG	CGCACCACACAAAAGAGACC
		CDS	ACGGTTTACTACGCCGTGTT	TGTAGGGTTGTTTCCGGACG
US12	ICP47	promoter	GATCGCATCGGAAAGGGACA	GGTGCTTACCCGTGCAAAAA
		CDS	TACCGGATTACGGGGACTGT	ATAAAAGGGGGCGTGAGGAC

**Table 3:** Primer sequences and target cellular genes.

Genes	Forward primers (5’-> 3’)		Reverse primers (5’-> 3’)
Glyceraldehyde 3-phosphate dehydrogenase (GAPDH)	GAGTCAACGGATTTGGTCGT	TTGATTTTGGAGGGATCTCG	
Enhancer 1 (Enh.1)	GCATGGTAGTCTCCCACTGATTT	CTGCAAATTCCTGCTGACTCAC	
leucine-zipper-like transcriptional regulator 1 (LTZR1)	GTGGGAAATGGGGGACCTTC	GCAGGAGGCCATCTTTCTTTG	
Myelin transcription factor 1 (MYT1)	CAGGAAGACACCTCTCACAC	ACAGTGTCCAGGGGCTTTGC	
Zinc-finger protein 554 (ZNF554)	CGGGGAAAAGCCCTATAAA	TCCACATTCACTGCATTCGT	
Family with sequence similarity 19 member A2 (FAM19A2)	TGCAATATACAGTGTGAGCAGTC	GCCCCTTCCCAGCTTATGAA	
Actin	CGGGAAATCGTGCGTGACATTAAG	GAACCGCTCATTGCCAATGGTGAT	

### Re-ChIP

Cells were processed similarly to ChIP until addition of the antibody. Two μg of the first antibody were pre-incubating with agarose beads coupled to protein A (Millipore 16–157) in PBS-0.5% BSA overnight at 4°C under shaking. The beads were washed twice with IP Buffer 1X (150mM NaCl, 5mM Tris pH8; 0.5mM EDTA; 0.1% SDS; 1% Triton), then the chromatin was incubated with the antibody/beads overnight at 4°C. Beads were then successively washed for 5 min at 4°C under constant shaking once in “low salt” buffer, once in “high salt” buffer, once in “LiCl” buffer and twice in TE buffer. Chromatin-antibody complexes were eluted at 37°C for 30 min with 100 μL of Re-ChIP elution buffer (1% SDS, 0.1M NaHCO3, 10mM DTT). Fifty μL were kept for analysis of the first capture efficiency, then the other 50 μL were diluted 20 times with IP buffer 1X and incubated overnight at 4°C with the second antibody pre-incubated with agarose beads coupled to protein A. The beads were washed twice with IP Buffer 1X, then successively washed for 5 min at 4°C under constant shaking, once in “low salt” buffer, once in “high salt” buffer, once in “LiCl” buffer and twice in TE buffer. Chromatin-antibody complexes were then eluted at 65°C for 30 min under constant shaking with 200 μL of elution buffer (1% SDS, 0.1M NaHCO3). Input and IP products were de-crosslinked overnight at 65°C a with 20 mg/mL of proteinase K (Sigma) and 10 mg/mL of RNAse A (Sigma). DNA was then purified by phenol-chloroform/ethanol precipitation, resuspended in water, and kept at -20°C until use for qPCR.

### siRNA transfections

Transfections of BJ cells with siRNAs was performed using Lipofectamine RNAiMAX and following the supplier’s procedure (Thermo Fisher Scientific). The following siRNAs were used at a final concentration of 40 nM for 48 h: siRNA_negative control (EUROGENTEC, FR-CL000-005), siHIRA 5’–GGAUAACACUGUCGUCAUC (Dharmacon: J-013610-07) [52]; siH3F3A: 5′-CUACAAAAGCCGCUCGCAA [100]; siH3F3B: 5′-GCUAAGAGAGUCACCAUCA [100].

### Antibodies

The antibodies used for immunofluorescence, ChIP and WB are provided in Tables 4–6.

**Table 4:** Antibodies used in immunofluorescence ^(^^1^^)^.

Proteins	Origin	References	Species	Dilution
Asf1a	Geneviève Almouzni (Institut Curie, Paris)	#28134	Rabbit polyclonal	1/1000
ATRX (h-300)	Santa Cruz	sc-15408	Rabbit polyclonal	1/100
c-Myc (9E10)	Santa cruz	sc-40	Mouse monoclonal	1/200
Cabin1	Sigma	HPA043296	Rabbit polyclonal	1/100
DAXX (M-112)	Santa Cruz	sc-7152	Rabbit polyclonal	1/100
HA	Abcam	ab9110	Rabbit polyclonal	1/500-1000
HA (3F10)	Roche	1867423	Rat monoclonal	(1/1000)
HIRA	Abcam	ab20655	Rabbit polyclonal	1/100
HIRA (WC119)	Millipore	04–1488	Mouse monoclonal	1/100
p48 (CAF-1)	Abcam	ab1765	Rabbit polyclonal	1/100
p60 (CAF-1)	Novus	500–207	Mouse monoclonal	1/100
p150 (CAF-1)	Novus	500–212	Mouse monoclonal	1/100
human PML (H-238)	Santa Cruz	sc-5621	Rabbit polyclonal	1/500
human PML(PG-M3)	Santa Cruz	sc-966	Mouse monoclonal	1/500
human PML 5E10	Roel Van Driel (University of Amsterdam)		Mouse monoclonal	1/500
mouse PML	Millipore	MAB3738	Mouse monoclonal	1/100
Sp100	Thomas Sternsdorf (University of Hamburg)	SpGH	Rabbit polyclonal	1/500
SUMO-1	Cell Signaling	4930	Rabbit polyclonal	1/500
SUMO-1 (5B12)	MBL	M113-3	Mouse monoclonal	1/500
SUMO-2/3 (18H8)	Cell Signaling	4971	Rabbit monoclonal	1/500
SUMO-2/3 (1E7)	MBL	M114-3	Mouse monoclonal	1/500
UBN1 Zap1	Henri Gruffat (CIRI, ENS, Lyon)		Mouse monoclonal	1/100

(1) All secondary antibodies were Alexa Fluor-conjugated and were raised in goats (Invitrogen).

**Table 5:** Antibodies used in ChIP.

Proteins	Origin	References	Species
ATRX (h-300)	Santa Cruz	sc-15408	Rabbit polyclonal
c-Myc (9E10)	Millipore	MABE282	Mouse monoclonal
H3.1/2	Millipore	ABE154	Rabbit polyclonal
H3.3	Millipore	09–838	Rabbit polyclonal
HA	Abcam	ab9110	Rabbit polyclonal
H3	Abcam	ab1791	Rabbit polyclonal
H4	Millipore	05–858	Rabbit monoclonal
H2A	Millipore	07–146	Rabbit polyclonal
H2B	Millipore	07–371	Rabbit polyclonal
H3K9me3	Abcam	ab8898	Rabbit polyclonal
H3K27me3	Abcam	ab6002	Mouse monoclonal
H3K4me2	Abcam	ab7766	Mouse monoclonal
IgG Mouse	Diagenode	Kch-819-015	Mouse monoclonal
IgG Rabbit	Diagenode	Kch-504-250	Rabbit polyclonal

**Table 6:** Antibodies used in WB ^(^^1^^)^.

Proteins	Origin	References	Species	Dilution
human PML (H-238)	Santa Cruz	sc-5621	Rabbit polyclonal	1/1000
ATRX (h-300)	Santa Cruz	sc-15408	Rabbit polyclonal	1/1000
DAXX (25C12)	Cell Signaling	#4533	Rabbit monoclonal	1/1000
HIRA (WC119)	Millipore	04–1488	Mouse monoclonal	1/1000
UBN1 Zap1	Henri Gruffat (CIRI, ENS, Lyon)		Mouse monoclonal	1/250
c-Myc (9E10)	Millipore	MABE282	Mouse monoclonal	1/1000
HA	Abcam	ab9110	Rabbit polyclonal	1/1000
Actin	Sigma	A2066	Rabbit polyclonal	1/1000

(1) All secondary antibodies were HRP-conjugated and were raised in goats (Sigma).

### Transcriptional reactivation procedure

BJ cells were transduced first with pLKOneo.CMV.EGFPnlsTetR to produce BJ-eTetR cell lines stably and constitutively expressing the EGFPnlsTetR protein (selection G418 1 mg/mL). BJ-eTetR cells were then transduced with pLKO.DCMV.TetO.cICP0 or pLKO.DCMV.TetO.cICP0ΔRF to produce BJ-eTetR/cICP0 or BJ-eTetR/cICP0ΔRF expressing ICP0 or its RING finger mutant FXE, respectively (selection puromycin 1 μg/mL). The expression of ICP0 or ICP0ΔRF was induced by the addition of doxycycline (100ng/μL) in the medium. BJ-eTetR, BJ-eTetR/cICP0 or BJ-eTetR/cICP0ΔRF were infected with HSV-1 *in*1374 for 4 days at 38.5°C to stabilize the formation of vDCP NBs. Then doxycycline was added or not in the medium to induce the expression of ICP0 or ICP0ΔRF. Cells were incubated at 32°C the permissive temperature for *in*1374 (see section virus). Twenty four hours, 48h or 72h after addition of doxycycline, the cells were fixed to proceed to IF or IF-FISH analyses or treated with FastLane cell SYBR Green RT-PCR (Qiagen 204243) to analyze the LacZ and viral transcripts by RT-qPCR.

## Supporting information

S1 Fig Latent/quiescent HSV-1 genomes co-localize with PML and PML NB-associated proteins in vDCP NBs. figImmuno-FISH performed in human primary fibroblasts (BJ cells) infected for 2 days with the replication-defective HSV-1 virus *in*1374. PML (i), Sp100 (ii), SUMO-1 (iii), SUMO 2/3 (iv), ATRX (v), DAXX (vi) (green), and HSV-1 genomes (red) were detected. Scale bars = 5 *μ*m.
(TIF)

S2 Fig Expression of tagged versions of DAXX (Myc), HIRA and UBN1 (HA) in BJ cells. Normal BJ cells were transduced with lentiviruses expressing Myc-DAXX, HIRA-HA, or HA-UBN1, and stable cell lines expressing the tagged proteins were selected by puromycin selection. Expression of the tagged proteins was detected by immunofluorescence (A) and Western blotting (B). For WB, actin was used as a loading control. Scale bars = 5 *μ*m.
(TIF)

S3 Fig Components of the DAXX/ATRX and HIRA complexes associate with latent/quiescent HSV-1 genomes at early times pi. (A) Schematic localization of the HSV-1 genome and of the loci analyzed by quantitative PCR (qPCR). UL: Unit Long, US: Unit Short, TRL: Terminal Repeat Long, TRS: Terminal Repeat Short, IRL: Inverted Repeat Long, IRS: Inverted Repeat Short. Immediate early (IE/α) genes (red), early (E/β) genes (green), late (L/γ) genes (blue).
(B) ChIP performed in *in*1374-infected normal BJ cells or *in*1374-infected BJ cells expressing tagged versions of DAXX, HIRA, or UBN1. Anti-myc (DAXX) or anti-HA (HIRA and UBN1) antibodies were used. Infections were performed for 30 min (blue), 2 h (red), 6 h (green). For ATRX, a native antibody was used, and the results were compared to ChIP with IgG as control. Analyzed viral loci were described previously. Means from three independent experiments ± SD.
(TIF)

S4 Fig Expression of the tagged H3.3 (e-H3.3) and H3.1 (e-H3.1) in BJ cells. (A) Detection of the protein expression by WB using the anti-HA antibody. Endogenous histone H3 was detected as a control. Actin was detected as a loading control.
(B) Detection of e-H3.1 (i) and e-H3.3 (ii) by immunofluorescence.
(C) Co-detection of e-H3.1 (i) and e-H3.3 (ii) (green) with PML (red). Nuclei are detected with DAPI (insets, gray) E-H3.3, unlike e-H3.1, co-localizes with PML NBs. Scale bars = 5 *μ*m.
(D) Quantification of the immunofluorescence experiments performed in (C). Means from two independent experiments.
(TIF)

S5 Fig Canonical histones associate with latent/quiescent HSV-1 genomes. ChIP performed in *in*1374-infected BJ cells at 24 hpi. Anti-H3, H4, H2A and H2B antibodies were used for ChIP experiments. Analyzed viral loci were described previously. Cellular locus glyceraldehyde 3-phosphate dehydrogenase (GAPDH) was analyzed as control.
(TIF)

S6 Fig The endogenous histone variant H3.3, but not the canonical H3.1/2, associates with latent/quiescent HSV-1 genomes. ChIP performed in *in*1374-infected BJ cells using control IgG (blue), anti-H3.1/2 (red), or anti-H3.3 (green) antibodies. Infections were performed for 24 h. Analyzed viral loci were described previously. Cellular loci Enhancer 1 (Enh.1), and leucine-zipper-like transcriptional regulator 1 (LZTR1) are positive controls for deposition of H3.3 and H3.1, respectively.
(TIF)

S7 Fig Validation of the shRNAs against DAXX, ATRX, HIRA, and UBN1. (A) BJ cells were transduced with shRNA-expressing lentiviruses before analysis 48 h post-transduction. RT-qPCR to detect DAXX, ATRX, HIRA, and UBN1 mRNA was performed, and the results were compared to a control shRNA (shCTRL). Means from three independent experiments ± SD. The Student’s *t*-test was applied to assess the significance of the results. * = p< 0.05, ** = p< 0.01.
(B) WB for detection of decreases in DAXX, ATRX, HIRA, and UBN1 proteins in normal BJ cells or BJ cells transduced with shRNA-expressing lentiviruses (48 h post-transduction). Actin was detected as a loading control. Two shRNAs were tested for each protein.
(TIF)

S8 Fig Effects of the depletion of DAXX, ATRX, HIRA or UBN1 on PML NB detection. BJ cells were transduced with shRNA-expressing lentiviruses before analysis 48 h post-transduction. Immunofluorescences were performed to detect DAXX, ATRX, HIRA, and UBN1 (green) and PML (gray, red). Two shRNAs were tested for each protein. Scale bars = 5 *μ*m.
(TIF)

S9 Fig Validation of the shRNAs against DAXX, ATRX, HIRA, and UBN1 in e-H3.3-expressing BJ cells. H3.3-expressing BJ cells were transduced with shRNA-expressing lentiviruses before analysis 48 h post-transduction.
(A) RT-qPCR to quantify DAXX, ATRX, HIRA, and UBN1 mRNA was performed, and the results were compared to a control shRNA (shCTRL). Data represent means from two independent experiments.
(B) WB for detection of decreases in ATRX, HIRA, and UBN1 proteins (48 h post-transduction) in normal e-H3.3-expressing BJ cells or e-H3.3-expressing BJ cells transduced with shRNA-expressing lentiviruses. Actin was detected as a loading control.
(TIF)

S10 Fig Inactivation of DAXX, ATRX, HIRA, or UBN1 does not affect the accumulation of e-H3.3 in PML NBs. Immunofluorescence experiments performed in e-H3.3-expressing BJ cells transduced with a lentivirus expressing a control shRNA (shCTRL, i, iii, v, vii) or a shRNA targeting DAXX (ii), ATRX (iv), HIRA (vi), or UBN1 (viii). E-H3.3 (gray, green); DAXX, ATRX, HIRA, UBN1 (gray, red); and PML (gray, blue) were detected. For the staining, a rat anti-HA mAb was used to detect e-H3.3, a rabbit polyclonal for the detection of DAXX, ATRX (i-iv), or PML (v-viii), and a mouse mAb for the detection of HIRA, UBN1 (v-viii) or PML (i-iv). Arrowheads point out examples of e-H3.3 co-localization with PML NBs in each sample. Scale bars = 5 *μ*m.
(TIF)

S11 Fig Validation of the shRNAs against PML in normal and e-H3.3-expressing BJ cells. Normal (A-C) or e-H3.3-expressing (D and E) BJ cells were transduced with a lentivirus expressing a control shRNA (shCTRL) or PML shRNAs (shPML) before analysis. Two different shRNAs were validated in normal BJ cells.
(A) Immunofluorescence to detect the PML NB signal. Scale bars = 5 *μ*m.
(B) RT-qPCR to quantify PML mRNA. Means from three independent experiments ± SD. The Student’s *t*-test was applied to assess the significance of the results. * = p< 0.05, ** = p< 0.01.
(C) WB to detect PML protein.
(D) RT-qPCR to quantify PML mRNA. Means from two independent experiments.
(E) WB to detect PML protein.
(TIF)

S12 Fig The decrease in the H3.3 association with latent/quiescent HSV-1 genomes is not compensated by H3.1. ChIP for the detection of e-H3.1 associated with HSV-1 in e-H3.1-expressing BJ cells previously transduced with a lentivirus expressing a shRNA control (shCTRL, blue) or a PML shRNA (shPML, red) (A) or e-H3.1-expressing MEF pml^-/-^ cells (B). Cells were infected with *in*1374 for 24 h. Anti-HA antibody was used for the ChIP experiments. The analyzed viral loci were described previously. Data represent means from two independent experiments ± SD.
(TIF)

S13 Fig IF for the detection of ICP0 or ICP0ΔRF (green) and PML (red) in BJ-eTetR, BJ-eTetR/cICP0 or BJ-eTetR/cICP0*μ*RF cells treated (+ dox) or not (no dox) with doxycycline for 24 h. ICP0 or ICP0*μ*RF (green), PML (red), and nuclei (DAPI, gray/blue) are detected. Expression of ICP0 but not ICP0*μ*RF induces the disappearance of PML-NBs. Scale bars = 5 *μ*m.
(TIF)

S14 Fig Phase contrast images showing the state of the cell monolayers in BJ-eTetR, BJ-eTetR/cICP0 or BJ-eTetR/cICP0*μ*RF cells not infected (NI) or infected with *in*1374 for 4 d at 38.5°C, then treated (+ dox) or not (no dox) with doxycycline for 0 h (T0), 24 h (T24), or 48 h (T48) at the permissive temperature (32°C) for in1374 replication. Expression of ICP0, but not ICP0*μ*RF or doxycycline alone, induces a cytopathic effect from T24. Scale bars = 50 *μ*m.
(TIF)

S15 Fig Immuno-FISH performed in BJ-eTetR or BJ-eTetR/cICP0ΔRF cells infected with *in*1374 for 4 d at 38.5°C, then treated (+ dox) or not (no dox) with doxycycline for 24 h (T24), or 48 h (T48), at 32°C. PML (green), HSV-1 genomes (gray/red), and nuclei (DAPI, gray/blue) are detected. Scale bars = 5 *μ*m.
(TIF)

S16 Fig The specific depletion of H3.3 does not affect the formation of vDCP NBs. (A) WB to visualize the depletion of H3.3 in e-H3.3-expressing BJ cells. A combination of two siRNAs targeting H3.3 transcripts from both H3.3-encoding genes (H3F3A and H3F3B) were used for the depletion of H3.3. Actin was detected as a loading control.
(B) Immunofluorescence performed in e-H3.3-expressing BJ cells transfected with control (siCTRL) or H3.3 (siH3F3A+3B) siRNAs. E-H3.3 (green), and PML (red) were detected. Nuclei were detected with DAPI (gray). Scale bars = 5 *μ*m.
(C) Quantifications of co-localizations of HSV-1 genomes with PML issued from immuno-FISH experiments performed in *in*1374-infected BJ cells (2 dpi) previously transfected with control (siCTRL) or H3.3 (siH3F3A+3B) siRNAs. Means from three independent experiments ± SD. The data suggest that vDCP-NBs are independent of H3.3 chromatinization of the latent/quiescent HSV-1 genomes for their formation.
(TIF)

S1 Table Co-localization between HSV-1 genomes and proteins in different cell types at 2 dpi. (TIF)

S2 Table Co-localization between HSV-1 genomes and PML-NBs-associated proteins/H3.3 chaperones. Short times pi (Means % ± SD).
(TIF)

S3 Table Co-localization between HSV-1 genomes and PML-NBs-associated proteins/H3.3 chaperones. Long times pi (Means % ± SD).
(TIF)

S4 Table Co-localization between HSV-1 genomes and PML in cells depleted for PML-NBs-associated proteins/ H3.3 chaperones (Means % ± SD). (TIF)

S5 Table Co-localization of latent/quiescent HSV-1 with DAXX, ATRX, HIRA, UBN1 in BJ cells depleted for PML (Means % ± SD). (TIF)

## References

[1] Whitley RJ, Roizman B. Herpes simplex virus infections. Lancet. 2001;357: 1513–1518. 10.1016/S0140-6736(00)04638-9 11377626

[2] Efstathiou S, Preston CM. Towards an understanding of the molecular basis of herpes simplex virus latency. Virus Res. 2005;111: 108–119. 10.1016/j.virusres.2005.04.017 15951043

[3] St Leger AJ, Peters B, Sidney J, Sette A, Hendricks RL. Defining the herpes simplex virus-specific CD8+ T cell repertoire in C57BL/6 mice. J Immunol. 2011;186: 3927–3933. 10.4049/jimmunol.1003735 21357536 PMC3308013

[4] van Velzen M, Jing L, Osterhaus ADME, Sette A, Koelle DM, Verjans GMGM. Local CD4 and CD8 T-cell reactivity to HSV-1 antigens documents broad viral protein expression and immune competence in latently infected human trigeminal ganglia. PLoS Pathog. 2013;9. 10.1371/journal.ppat.1003547 PMC3744444 23966859

[5] Douglas MW, Diefenbach RJ, Homa FL, Miranda-Saksena M, Rixon FJ, Vittone V, et al Herpes simplex virus type 1 capsid protein VP26 interacts with dynein light chains RP3 and Tctex1 and plays a role in retrograde cellular transport. The Journal of biological chemistry. American Society for Biochemistry and Molecular Biology; 2004;279: 28522–28530. 10.1074/jbc.M311671200 15117959

[6] Sodeik B, Ebersold MW, Helenius A. Microtubule-mediated transport of incoming herpes simplex virus 1 capsids to the nucleus. J Cell Biol. 1997;136: 1007–1021. 9060466 10.1083/jcb.136.5.1007 PMC2132479

[7] Döhner K, Radtke K, Schmidt S, Sodeik B. Eclipse phase of herpes simplex virus type 1 infection: Efficient dynein-mediated capsid transport without the small capsid protein VP26. J Virol. 2006;80: 8211–8224. 10.1128/JVI.02528-05 16873277 PMC1563788

[8] Koyuncu OO, Hogue IB, Enquist LW. Virus infections in the nervous system. Cell Host Microbe. Elsevier; 2013;13: 379–393. 10.1016/j.chom.2013.03.010 PMC3647473 23601101

[9] Kramer T, Enquist LW. Directional spread of alphaherpesviruses in the nervous system. Viruses. 2013;5: 678–707. 10.3390/v5020678 23435239 PMC3640521

[10] Taylor MP, Enquist LW. Axonal spread of neuroinvasive viral infections. Trends Microbiol. 2015;23: 288. 10.1016/j.tim.2015.01.002 PMC4417403 25639651

[11] Sears AE, Hukkanen V, Labow MA, Levine AJ, Roizman B. Expression of the herpes simplex virus 1 alpha transinducing factor (VP16) does not induce reactivation of latent virus or prevent the establishment of latency in mice. J Virol. American Society for Microbiology (ASM); 1991;65: 2929–2935. 1851865 10.1128/jvi.65.6.2929-2935.1991 PMC240928

[12] Luxton GWG, Haverlock S, Coller KE, Antinone SE, Pincetic A, Smith GA. Targeting of herpesvirus capsid transport in axons is coupled to association with specific sets of tegument proteins. Proc Natl Acad Sci USA. 2005;102: 5832–5837. 10.1073/pnas.0500803102 15795370 PMC556296

[13] Aggarwal A, Miranda-Saksena M, Boadle RA, Kelly BJ, Diefenbach RJ, Alam W, et al Ultrastructural visualization of individual tegument protein dissociation during entry of herpes simplex virus 1 into human and rat dorsal root ganglion neurons. J Virol. 2012;86: 6123–6137. 10.1128/JVI.07016-11 22457528 PMC3372220

[14] Sawtell NM, Thompson RL. De Novo Herpes Simplex Virus VP16 Expression Gates a Dynamic Programmatic Transition and Sets the Latent/Lytic Balance during Acute Infection in Trigeminal Ganglia. PLoS Pathog. Public Library of Science; 2016;12: e1005877 10.1371/journal.ppat.1005877 27607440 PMC5015900

[15] Alandijany T, Roberts APE, Conn KL, Loney C, McFarlane S, Orr A, et al Distinct temporal roles for the promyelocytic leukaemia (PML) protein in the sequential regulation of intracellular host immunity to HSV-1 infection. Hutt-Fletcher L, editor. PLoS Pathog. Public Library of Science; 2018;14: e1006769 10.1371/journal.ppat.1006769 29309427 PMC5757968

[16] Catez F, Picard C, Held K, Gross S, Rousseau A, Theil D, et al HSV-1 Genome Subnuclear Positioning and Associations with Host-Cell PML-NBs and Centromeres Regulate LAT Locus Transcription during Latency in Neurons. PLoS Pathog. 2012;8: e1002852 10.1371/journal.ppat.1002852 22912575 PMC3415458

[17] Maroui M-A, Callé A, Cohen C, Streichenberger N, Texier P, Takissian J, et al Latency Entry of Herpes Simplex Virus 1 Is Determined by the Interaction of Its Genome with the Nuclear Environment. PLoS Pathog. Public Library of Science; 2016;12: e1005834 10.1371/journal.ppat.1005834 27618691 PMC5019400

[18] Mehta A, Maggioncalda J, Bagasra O, Thikkavarapu S, Saikumari P, Valyi-Nagy T, et al In situ DNA PCR and RNA hybridization detection of herpes simplex virus sequences in trigeminal ganglia of latently infected mice. Virology. 1995;206: 633–640. 7831818 10.1016/s0042-6822(95)80080-8

[19] Sawtell NM. Comprehensive quantification of herpes simplex virus latency at the single-cell level. J Virol. 1997 ed. 1997;71: 5423–5431. 9188614 10.1128/jvi.71.7.5423-5431.1997 PMC191782

[20] Sawtell NM, Poon DK, Tansky CS, Thompson RL. The latent herpes simplex virus type 1 genome copy number in individual neurons is virus strain specific and correlates with reactivation. J Virol. 1998 ed. 1998;72: 5343–5350. 9620987 10.1128/jvi.72.7.5343-5350.1998 PMC110155

[21] Chen X-P, Mata M, Kelley M, Glorioso JC, Fink DJ. The relationship of herpes simplex virus latency associated transcript expression to genome copy number: a quantitative study using laser capture microdissection. J Neurovirol. 2002;8: 204–210. 10.1080/13550280290049642 12053275

[22] Wang K, Lau TY, Morales M, Mont EK, Straus SE. Laser-capture microdissection: refining estimates of the quantity and distribution of latent herpes simplex virus 1 and varicella-zoster virus DNA in human trigeminal Ganglia at the single-cell level. J Virol. 2005 ed. 2005;79: 14079–14087. 10.1128/JVI.79.22.14079-14087.2005 16254342 PMC1280223

[23] Proenca JT, Coleman HM, Connor V, Winton DJ, Efstathiou S. A historical analysis of herpes simplex virus promoter activation in vivo reveals distinct populations of latently infected neurones. The Journal of general virology. 2008;89: 2965–2974. 10.1099/vir.0.2008/005066-0 19008381 PMC2885028

[24] Proenca JT, Coleman HM, Nicoll MP, Connor V, Preston CM, Arthur J, et al An investigation of HSV promoter activity compatible with latency establishment reveals VP16 independent activation of HSV immediate early promoters in sensory neurones. The Journal of general virology. 2011;92: 2575–2585. 10.1099/vir.0.034728-0 21752961 PMC3541806

[25] Held K, Junker A, Dornmair K, Meinl E, Sinicina I, Brandt T, et al Expression of herpes simplex virus 1-encoded microRNAs in human trigeminal ganglia and their relation to local T-cell infiltrates. J Virol. 2011;85: 9680–9685. 10.1128/JVI.00874-11 21795359 PMC3196425

[26] Bloom DC, Giordani NV, Kwiatkowski DL. Epigenetic regulation of latent HSV-1 gene expression. Biochimica et biophysica acta. 2010 ed. 2010;1799: 246–256. 10.1016/j.bbagrm.2009.12.001 20045093 PMC2838971

[27] Kristie TM, Liang Y, Vogel JL. Control of alpha-herpesvirus IE gene expression by HCF-1 coupled chromatin modification activities. Biochimica et biophysica acta. 2009 ed. 2010;1799: 257–265. 10.1016/j.bbagrm.2009.08.003 19682612 PMC2838944

[28] Knipe DM, Lieberman PM, Jung JU, McBride AA, Morris KV, Ott M, et al Snapshots: chromatin control of viral infection. Virology. 2013;435: 141–156. 10.1016/j.virol.2012.09.023 23217624 PMC3531885

[29] Deshmane SL, Fraser NW. During latency, herpes simplex virus type 1 DNA is associated with nucleosomes in a chromatin structure. J Virol. 1989;63: 943–947. 2536115 10.1128/jvi.63.2.943-947.1989 PMC247770

[30] Kubat NJ, Tran RK, McAnany P, Bloom DC. Specific histone tail modification and not DNA methylation is a determinant of herpes simplex virus type 1 latent gene expression. J Virol. 2004 ed. 2004;78: 1139–1149. 10.1128/JVI.78.3.1139-1149.2004 14722269 PMC321404

[31] Wang Q-Y, Zhou C, Johnson KE, Colgrove RC, Coen DM, Knipe DM. Herpesviral latency-associated transcript gene promotes assembly of heterochromatin on viral lytic-gene promoters in latent infection. Proc Natl Acad Sci USA. 2005;102: 16055–16059. 10.1073/pnas.0505850102 16247011 PMC1266038

[32] Knipe DM, Cliffe A. Chromatin control of herpes simplex virus lytic and latent infection. Nature reviews. 2008;6: 211–221. 10.1038/nrmicro1794 18264117

[33] Cliffe AR, Garber DA, Knipe DM. Transcription of the herpes simplex virus latency-associated transcript promotes the formation of facultative heterochromatin on lytic promoters. J Virol. 2009 ed. American Society for Microbiology; 2009;83: 8182–8190. 10.1128/JVI.00712-09 19515781 PMC2715743

[34] Kwiatkowski DL, Thompson HW, Bloom DC. The polycomb group protein Bmi1 binds to the herpes simplex virus 1 latent genome and maintains repressive histone marks during latency. J Virol. 2009 ed. American Society for Microbiology; 2009;83: 8173–8181. 10.1128/JVI.00686-09 19515780 PMC2715759

[35] Tagami H, Ray-Gallet D, Almouzni G, Nakatani Y. Histone H3.1 and H3.3 complexes mediate nucleosome assembly pathways dependent or independent of DNA synthesis. Cell. 2004;116: 51–61. 14718166 10.1016/s0092-8674(03)01064-x

[36] Szenker E, Ray-Gallet D, Almouzni G. The double face of the histone variant H3.3. Cell Res. 2011;21: 421–434. 10.1038/cr.2011.14 21263457 PMC3193428

[37] Wong LH, McGhie JD, Sim M, Anderson MA, Ahn S, Hannan RD, et al ATRX interacts with H3.3 in maintaining telomere structural integrity in pluripotent embryonic stem cells. Genome Res. 2010;20: 351–360. 10.1101/gr.101477.109 20110566 PMC2840985

[38] Goldberg AD, Banaszynski LA, Noh K-M, Lewis PW, Elsaesser SJ, Stadler S, et al Distinct factors control histone variant H3.3 localization at specific genomic regions. Cell. 2010;140: 678–691. 10.1016/j.cell.2010.01.003 20211137 PMC2885838

[39] Drané P, Ouararhni K, Depaux A, Shuaib M, Hamiche A. The death-associated protein DAXX is a novel histone chaperone involved in the replication-independent deposition of H3.3. Genes & development. 2010;24: 1253–1265. 20504901 10.1101/gad.566910 PMC2885661

[40] Banumathy G, Somaiah N, Zhang R, Tang Y, Hoffmann J, Andrake M, et al Human UBN1 is an ortholog of yeast Hpc2p and has an essential role in the HIRA/ASF1a chromatin-remodeling pathway in senescent cells. Molecular and cellular biology. 2009;29: 758–770. 10.1128/MCB.01047-08 19029251 PMC2630694

[41] Rai TS, Puri A, McBryan T, Hoffman J, Tang Y, Pchelintsev NA, et al Human CABIN1 is a functional member of the human HIRA/UBN1/ASF1a histone H3.3 chaperone complex. Molecular and cellular biology. 2011;31: 4107–4118. 10.1128/MCB.05546-11 21807893 PMC3187368

[42] Delbarre E, Ivanauskiene K, Küntziger T, Collas P. DAXX-dependent supply of soluble (H3.3-H4) dimers to PML bodies pending deposition into chromatin. Genome Res. 2013;23: 440–451. 10.1101/gr.142703.112 23222847 PMC3589533

[43] Corpet A, Olbrich T, Gwerder M, Fink D, Stucki M. Dynamics of histone H3.3 deposition in proliferating and senescent cells reveals a DAXX-dependent targeting to PML-NBs important for pericentromeric heterochromatin organization. Cell cycle (Georgetown), Tex. 2013;13: 249–267. 10.4161/cc.26988 PMC3906242 24200965

[44] Delbarre E, Ivanauskiene K, Spirkoski J, Shah A, Vekterud K, Moskaug JØ, et al PML protein organizes heterochromatin domains where it regulates histone H3.3 deposition by ATRX/DAXX. Genome Res. Cold Spring Harbor Lab; 2017;: gr.215830.116. 10.1101/gr.215830.116 PMC5453325 28341773

[45] Everett RD, Murray J, Orr A, Preston CM. Herpes simplex virus type 1 genomes are associated with ND10 nuclear substructures in quiescently infected human fibroblasts. J Virol. 2007 ed. American Society for Microbiology; 2007;81: 10991–11004. 10.1128/JVI.00705-07 17670833 PMC2045565

[46] Jamieson DR, Robinson LH, Daksis JI, Nicholl MJ, Preston CM. Quiescent viral genomes in human fibroblasts after infection with herpes simplex virus type 1 Vmw65 mutants. The Journal of general virology. 1995 ed. 1995;76 (Pt 6): 1417–1431. 7782770 10.1099/0022-1317-76-6-1417

[47] Preston CM, Nicholl MJ. Repression of gene expression upon infection of cells with herpes simplex virus type 1 mutants impaired for immediate-early protein synthesis. J Virol. 1997;71: 7807–13. 9311867 10.1128/jvi.71.10.7807-7813.1997 PMC192134

[48] Samaniego LA, Neiderhiser L, DeLuca NA. Persistence and expression of the herpes simplex virus genome in the absence of immediate-early proteins. J Virol. 1998;72: 3307–20. 9525658 10.1128/jvi.72.4.3307-3320.1998 PMC109808

[49] Ferenczy MW, Deluca NA. Epigenetic modulation of gene expression from quiescent herpes simplex virus genomes. J Virol. 2009 ed. American Society for Microbiology; 2009;83: 8514–8524. 10.1128/JVI.00785-09 19535445 PMC2738166

[50] Jackson SA, DeLuca NA. Relationship of herpes simplex virus genome configuration to productive and persistent infections. Proc Natl Acad Sci USA. 2003;100: 7871–7876. 10.1073/pnas.1230643100 12796511 PMC164680

[51] Ray-Gallet D, Quivy J-P, Scamps C, Martini EM-D, Lipinski M, Almouzni G. HIRA is critical for a nucleosome assembly pathway independent of DNA synthesis. Molecular cell. 2002;9: 1091–1100. 12049744 10.1016/s1097-2765(02)00526-9

[52] Ray-Gallet D, Woolfe A, Vassias I, Pellentz C, Lacoste N, Puri A, et al Dynamics of histone h3 deposition in vivo reveal a nucleosome gap-filling mechanism for h3.3 to maintain chromatin integrity. Molecular cell. 2011;44: 928–941. 10.1016/j.molcel.2011.12.006 22195966

[53] Lomonte P. The interaction between herpes simplex virus 1 genome and promyelocytic leukemia nuclear bodies (PML-NBs) as a hallmark of the entry in latency. Microb Cell. 2016;3: 569–572. doi: 10.15698/mic2016.11.541 28357326 10.15698/mic2016.11.541 PMC5349213

[54] Lomonte P. Herpesvirus Latency: On the Importance of Positioning Oneself. Adv Anat Embryol Cell Biol. Cham: Springer International Publishing; 2017;223: 95–117. 10.1007/978-3-319-53168-7_5 28528441

[55] Dembowski JA, Deluca NA. Temporal Viral Genome-Protein Interactions Define Distinct Stages of Productive Herpesviral Infection. Imperiale MJ, editor. MBio. 2018;9: 90. 10.1128/mBio.01182-18 PMC6050965 30018111

[56] Rai TS, Glass M, Cole JJ, Rather MI, Marsden M, Neilson M, et al Histone chaperone HIRA deposits histone H3.3 onto foreign viral DNA and contributes to anti-viral intrinsic immunity. Nucleic acids research. 2017. 10.1093/nar/gkx771 PMC5691367 28981850

[57] Zhang H, Gan H, Wang Z, Lee J-H, Zhou H, Ordog T, et al RPA Interacts with HIRA and Regulates H3.3 Deposition at Gene Regulatory Elements in Mammalian Cells. Molecular cell. Elsevier; 2017;65: 272–284. 10.1016/j.molcel.2016.11.030 28107649 PMC5460635

[58] Albright ER, Kalejta RF. Canonical and variant forms of histone H3 are deposited onto the human cytomegalovirus genome during lytic and latent infections. J Virol. 2016. 10.1128/JVI.01220-16 PMC5105665 27605676

[59] Ferenczy MW, Deluca NA. Reversal of Heterochromatic Silencing of Quiescent Herpes Simplex Virus Type 1 by ICP0. J Virol. 2011;85: 3424–3435. 10.1128/JVI.02263-10 21191021 PMC3067893

[60] Ferenczy MW, Ranayhossaini DJ, DeLuca NA. Activities of Icp0 Involved in the Reversal of Silencing of Quiescent Hsv-1. J Virol. 2011 ed. 2011. 10.1128/JVI.02265-10 PMC3126212 21411540

[61] Blahnik KR, Dou L, Echipare L, Iyengar S, O'Geen H, Sanchez E, et al Characterization of the contradictory chromatin signatures at the 3' exons of zinc finger genes. Wutz A, editor. PloS one. Public Library of Science; 2011;6: e17121 10.1371/journal.pone.0017121 21347206 PMC3039671

[62] Kirmizis A, Bartley SM, Kuzmichev A, Margueron R, Reinberg D, Green R, et al Silencing of human polycomb target genes is associated with methylation of histone H3 Lys 27. Genes & development. Cold Spring Harbor Lab; 2004;18: 1592–1605. 15231737 10.1101/gad.1200204 PMC443521

[63] Banumathy G, Somaiah N, Zhang R, Tang Y, Hoffmann J, Andrake M, et al Human UBN1 Is an Ortholog of Yeast Hpc2p and Has an Essential Role in the HIRA/ASF1a Chromatin-Remodeling Pathway in Senescent Cells. Molecular and cellular biology. 2009;29: 758–770. 10.1128/MCB.01047-08 19029251 PMC2630694

[64] Ricketts MD, Frederick B, Hoff H, Tang Y, Schultz DC, Singh Rai T, et al Ubinuclein-1 confers histone H3.3-specific-binding by the HIRA histone chaperone complex. Nat Commun. 2015;6: 7711–11. 10.1038/ncomms8711 26159857 PMC4510971

[65] Cuchet-Lourenço D, Vanni E, Glass M, Orr A, Everett RD. Herpes simplex virus 1 ubiquitin ligase ICP0 interacts with PML isoform I and induces its SUMO-independent degradation. J Virol. 2012;86: 11209–11222. 10.1128/JVI.01145-12 22875967 PMC3457127

[66] Everett RD, Maul GG. HSV-1 IE protein Vmw110 causes redistribution of PML. The EMBO journal. 1994;13: 5062–5069. 7957072 10.1002/j.1460-2075.1994.tb06835.x PMC395452

[67] Halford WP, Schaffer PA. ICP0 is required for efficient reactivation of herpes simplex virus type 1 from neuronal latency. J Virol. 2001;75: 3240–9. 10.1128/JVI.75.7.3240-3249.2001 11238850 PMC114117

[68] Placek BJ, Huang J, Kent JR, Dorsey J, Rice L, Fraser NW, et al The histone variant H3.3 regulates gene expression during lytic infection with herpes simplex virus type 1. J Virol. 2008 ed. 2009;83: 1416–1421. 10.1128/JVI.01276-08 19004946 PMC2620911

[69] Oh J, Ruskoski N, Fraser NW. Chromatin assembly on herpes simplex virus 1 DNA early during a lytic infection is Asf1a dependent. J Virol. 2012;86: 12313–12321. 10.1128/JVI.01570-12 22951827 PMC3486495

[70] Tang Y, Puri A, Ricketts MD, Rai TS, Hoffmann J, Hoi E, et al Identification of an ubinuclein 1 region required for stability and function of the human HIRA/UBN1/CABIN1/ASF1a histone H3.3 chaperone complex. Biochemistry. American Chemical Society; 2012;51: 2366–2377. 10.1021/bi300050b PMC3320765 22401310

[71] Adam S, Polo SE, Almouzni G. Transcription Recovery after DNA Damage Requires Chromatin Priming by the H3.3 Histone Chaperone HIRA. Cell. 2013;155: 94–106. 10.1016/j.cell.2013.08.029 24074863

[72] Adam S, Dabin J, Chevallier O, Leroy O, Baldeyron C, Corpet A, et al Real-Time Tracking of Parental Histones Reveals Their Contribution to Chromatin Integrity Following DNA Damage. Molecular cell. 2016;64: 65–78. 10.1016/j.molcel.2016.08.019 27642047 PMC5065526

[73] Wilkie NM. The synthesis and substructure of herpesvirus DNA: the distribution of alkali-labile single strand interruptions in HSV-1 DNA. The Journal of general virology. Microbiology Society; 1973;21: 453–467. 10.1099/0022-1317-21-3-453 4357936

[74] Orzalli MH, Conwell SE, Berrios C, DeCaprio JA, Knipe DM. Nuclear interferon-inducible protein 16 promotes silencing of herpesviral and transfected DNA. Proc Natl Acad Sci USA. 2013;110: 501 10.1073/pnas.1201390110 24198334 PMC3839728

[75] Unterholzner L, Keating SE, Baran M, Horan KA, Jensen SB, Sharma S, et al IFI16 is an innate immune sensor for intracellular DNA. Nat Immunol. 2010;11: 997–1004. 10.1038/ni.1932 20890285 PMC3142795

[76] Kerur N, Veettil MV, Sharma-Walia N, Bottero V, Sadagopan S, Otageri P, et al IFI16 acts as a nuclear pathogen sensor to induce the inflammasome in response to Kaposi Sarcoma-associated herpesvirus infection. Cell Host Microbe. 2011;9: 363–375. 10.1016/j.chom.2011.04.008 21575908 PMC3113467

[77] Gariano GR, Dell'Oste V, Bronzini M, Gatti D, Luganini A, De Andrea M, et al The intracellular DNA sensor IFI16 gene acts as restriction factor for human cytomegalovirus replication. PLoS Pathog. 2012;8: e1002498 10.1371/journal.ppat.1002498 22291595 PMC3266931

[78] Orzalli MH, Deluca NA, Knipe DM. Nuclear IFI16 induction of IRF-3 signaling during herpesviral infection and degradation of IFI16 by the viral ICP0 protein. Proc Natl Acad Sci USA. National Acad Sciences; 2012;109: E3008–17. 10.1073/pnas.1211302109 23027953 PMC3497734

[79] Johnson KE, Chikoti L, Chandran B. Herpes simplex virus 1 infection induces activation and subsequent inhibition of the IFI16 and NLRP3 inflammasomes. J Virol. 2013;87: 5005–5018. 10.1128/JVI.00082-13 23427152 PMC3624293

[80] Ansari MA, Singh VV, Dutta S, Veettil MV, Dutta D, Chikoti L, et al Constitutive interferon-inducible protein 16-inflammasome activation during Epstein-Barr virus latency I, II, and III in B and epithelial cells. J Virol. 2013;87: 8606–8623. 10.1128/JVI.00805-13 23720728 PMC3719826

[81] Dutta D, Dutta S, Veettil MV, Roy A, Ansari MA, Iqbal J, et al BRCA1 Regulates IFI16 Mediated Nuclear Innate Sensing of Herpes Viral DNA and Subsequent Induction of the Innate Inflammasome and Interferon-β Responses. Feng P, editor. PLoS Pathog. 2015;11: e1005030 10.1371/journal.ppat.1005030 26121674 PMC4487893

[82] Diner BA, Li T, Greco TM, Crow MS, Fuesler JA, Wang J, et al The functional interactome of PYHIN immune regulators reveals IFIX is a sensor of viral DNA. Mol Syst Biol. European Molecular Biology Organization; 2015;11: 787–787. doi: 10.15252/msb.20145808 25665578 10.15252/msb.20145808 PMC4358659

[83] Tsai K, Chan L, Gibeault R, Conn K, Dheekollu J, Domsic J, et al Viral Reprogramming of the Daxx-Histone H3.3 Chaperone During EBV Early Infection. J Virol. American Society for Microbiology; 2014;88: 14350–14363. 10.1128/JVI.01895-14 25275136 PMC4249116

[84] Schreiner S, Bürck C, Glass M, Groitl P, Wimmer P, Kinkley S, et al Control of human adenovirus type 5 gene expression by cellular Daxx/ATRX chromatin-associated complexes. Nucleic acids research. 2013. 10.1093/nar/gkt064 PMC3616723 23396441

[85] Labetoulle M, Maillet S, Efstathiou S, Dezelee S, Frau E, Lafay F. HSV1 latency sites after inoculation in the lip: assessment of their localization and connections to the eye. Invest Ophthalmol Vis Sci. 2003;44: 217–225. 12506078 10.1167/iovs.02-0464

[86] Preston CM. Abnormal properties of an immediate early polypeptide in cells infected with the herpes simplex virus type 1 mutant tsK. J Virol. 1979;32: 357–369. 228063 10.1128/jvi.32.2.357-369.1979 PMC353566

[87] Ace CI, McKee TA, Ryan JM, Cameron JM, Preston CM. Construction and characterization of a herpes simplex virus type 1 mutant unable to transinduce immediate-early gene expression. J Virol. 1989;63: 2260–2269. 2539517 10.1128/jvi.63.5.2260-2269.1989 PMC250644

[88] Preston CM, Rinaldi A, Nicholl MJ. Herpes simplex virus type 1 immediate early gene expression is stimulated by inhibition of protein synthesis. The Journal of general virology. 1998;79 (Pt 1): 117–124. 9460932 10.1099/0022-1317-79-1-117

[89] Preston CM, Nicholl MJ. Human Cytomegalovirus Tegument Protein pp71 Directs Long-Term Gene Expression from Quiescent Herpes Simplex Virus Genomes. J Virol. 2005;79: 525–535. 10.1128/JVI.79.1.525-535.2005 15596845 PMC538741

[90] McFarlane M, Daksis JI, Preston CM. Hexamethylene bisacetamide stimulates herpes simplex virus immediate early gene expression in the absence of trans-induction by Vmw65. The Journal of general virology. 1992;73 (Pt 2): 285–292. 1371540 10.1099/0022-1317-73-2-285

[91] Wang ZG, Ruggero D, Ronchetti S, Zhong S, Gaboli M, Rivi R, et al PML is essential for multiple apoptotic pathways. Nat Genet. 1998;20: 266–272. 10.1038/3073 9806545

[92] Catez F, Rousseau A, Labetoulle M, Lomonte P. Detection of the genome and transcripts of a persistent DNA virus in neuronal tissues by fluorescent in situ hybridization combined with immunostaining. J Vis Exp. 2014;: e51091 10.3791/51091 24514006 PMC4089569

[93] Sawtell NM, Thompson RL. Comparison of herpes simplex virus reactivation in ganglia in vivo and in explants demonstrates quantitative and qualitative differences. J Virol. 2004 ed. 2004;78: 7784–7794. 10.1128/JVI.78.14.7784-7794.2004 15220452 PMC434126

[94] Cunningham C, Davison AJ. A cosmid-based system for constructing mutants of herpes simplex virus type 1. Virology. 1993;197: 116–124. 10.1006/viro.1993.1572 8212547

[95] Pear W. Transient transfection methods for preparation of high-titer retroviral supernatants Curr Protoc Mol Biol. Hoboken, NJ, USA: John Wiley & Sons, Inc; 2001;Chapter 9: Unit9.11. 10.1002/0471142727.mb0911s36 18265279

[96] Naviaux RK, Costanzi E, Haas M, Verma IM. The pCL vector system: rapid production of helper-free, high-titer, recombinant retroviruses. J Virol. 1996;70: 5701–5705. 8764092 10.1128/jvi.70.8.5701-5705.1996 PMC190538

[97] Sambrook J, Russell DW. Calcium-phosphate-mediated Transfection of Eukaryotic Cells with Plasmid DNAs Sambrook J Russell D, editors. CSH Protoc 2006;2006: pdb.prot3871. 10.1101/pdb.prot3871 22485343

[98] Everett RD, Parsy M-L, Orr A. Analysis of the functions of herpes simplex virus type 1 regulatory protein ICP0 that are critical for lytic infection and derepression of quiescent viral genomes. J Virol. 2009 ed. American Society for Microbiology; 2009;83: 4963–4977. 10.1128/JVI.02593-08 19264778 PMC2682082

[99] Cantrell SR, Bresnahan WA. Human cytomegalovirus (HCMV) UL82 gene product (pp71) relieves hDaxx-mediated repression of HCMV replication. J Virol. 2006;80: 6188–6191. 10.1128/JVI.02676-05 16731959 PMC1472601

[100] Zhang R, Liu S-T, Chen W, Bonner M, Pehrson J, Yen TJ, et al HP1 proteins are essential for a dynamic nuclear response that rescues the function of perturbed heterochromatin in primary human cells. Molecular and cellular biology. 2007;27: 949–962. 10.1128/MCB.01639-06 17101789 PMC1800672

