# Supplementary figures and images for: Promyelocytic leukemia (PML) nuclear bodies (NBs) induce latent/quiescent HSV-1 genomes chromatinization through a PML NB/Histone H3.3/H3.3 Chaperone Axis
Source: PLoS Pathog. 2018 Sep 20;14(9):e1007313. doi: 10.1371/journal.ppat.1007313 (PMC6168178; doi:10.1371/journal.ppat.1007313)

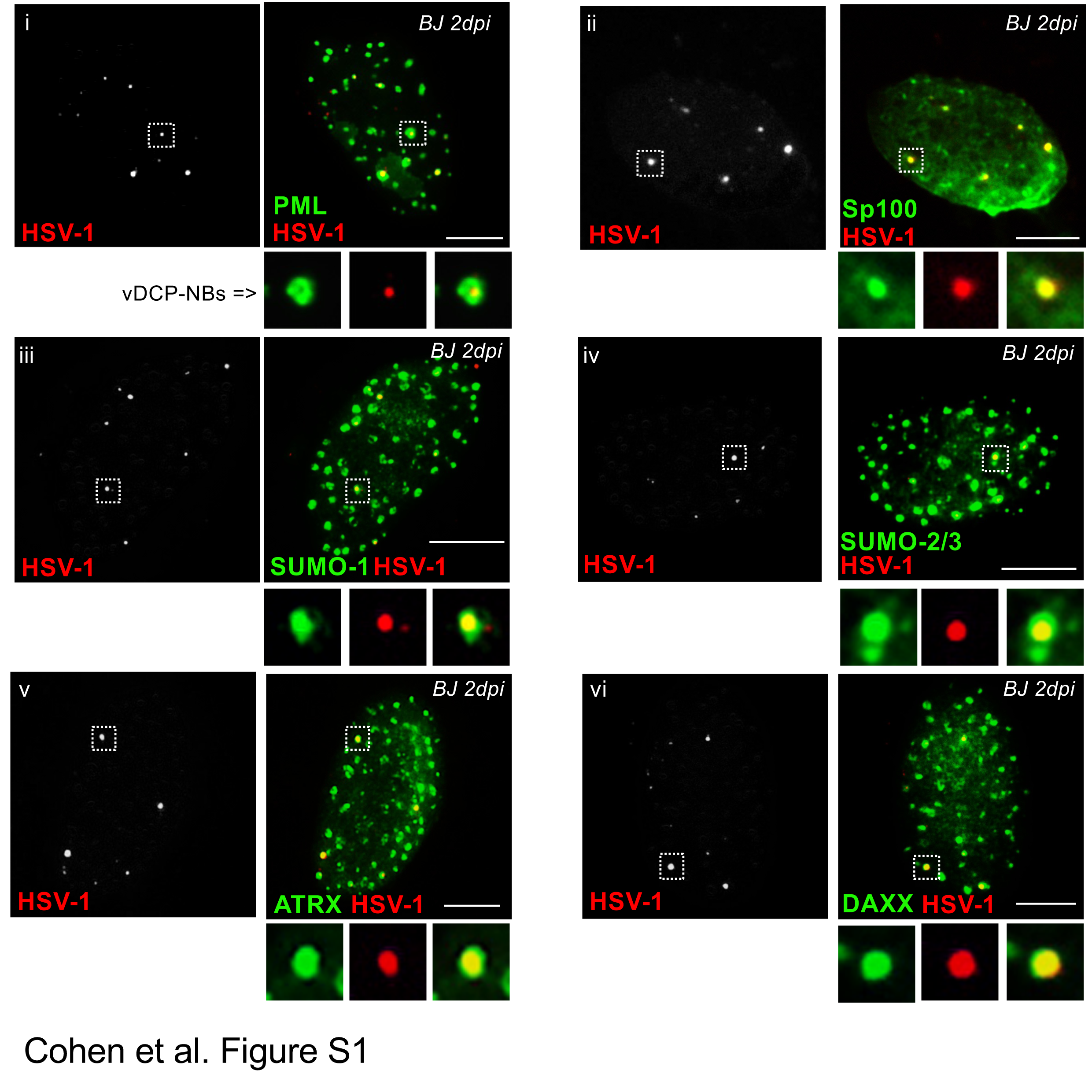

Supplement: S1 Fig — figImmuno-FISH performed in human primary fibroblasts (BJ cells) infected for 2 days with the replication-defective HSV-1 virus in1374. PML (i), Sp100 (ii), SUMO-1 (iii), SUMO 2/3 (iv), ATRX (v), DAXX (vi) (green), and HSV-1 genomes (red) were detected. Scale bars = 5 μm. (TIF) [file ppat.1007313.s001.tif]

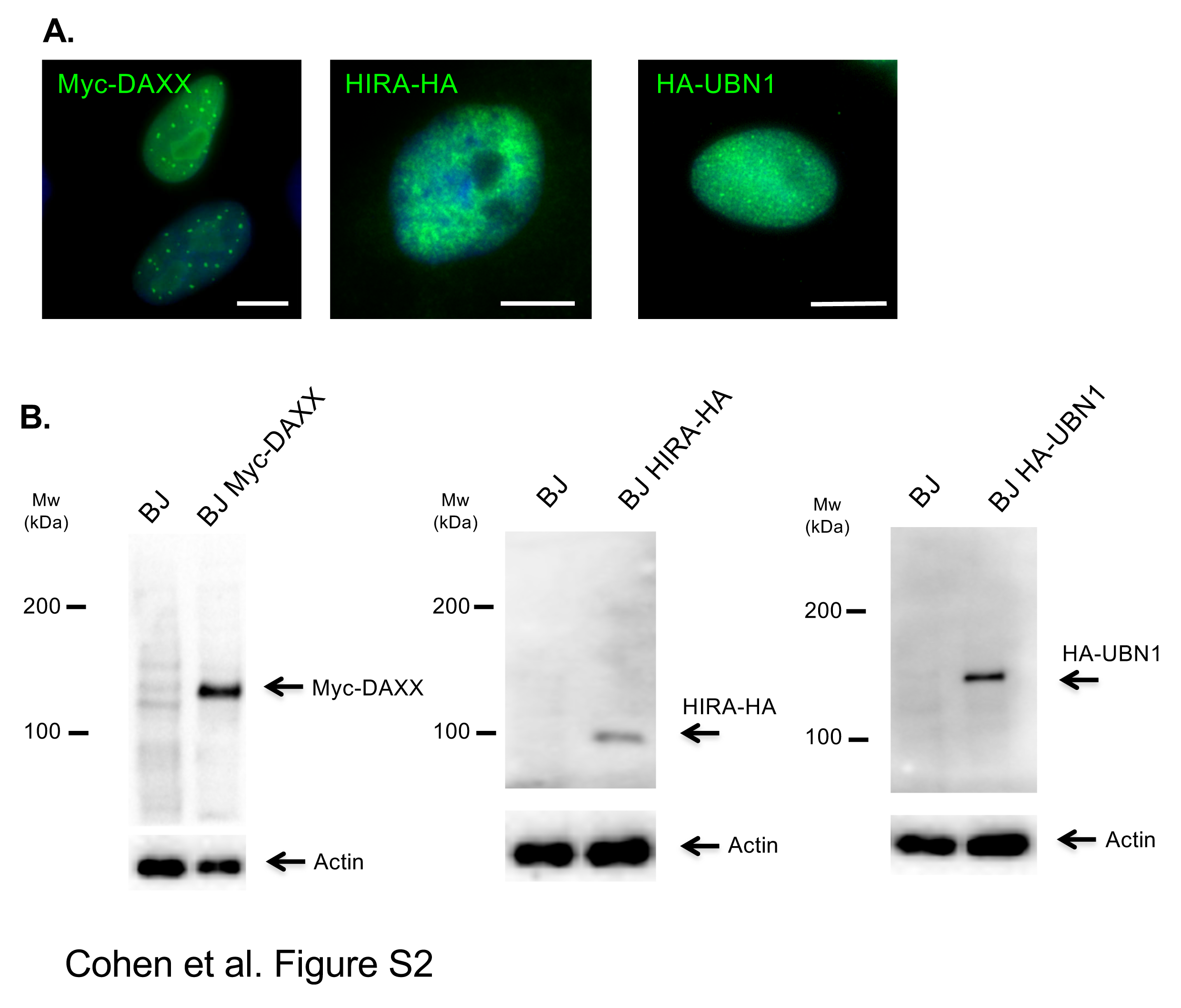

Supplement: S2 Fig — Normal BJ cells were transduced with lentiviruses expressing Myc-DAXX, HIRA-HA, or HA-UBN1, and stable cell lines expressing the tagged proteins were selected by puromycin selection. Expression of the tagged proteins was detected by immunofluorescence (A) and Western blotting (B). For WB, actin was used as a loading control. Scale bars = 5 μm. (TIF) [file ppat.1007313.s002.tif]

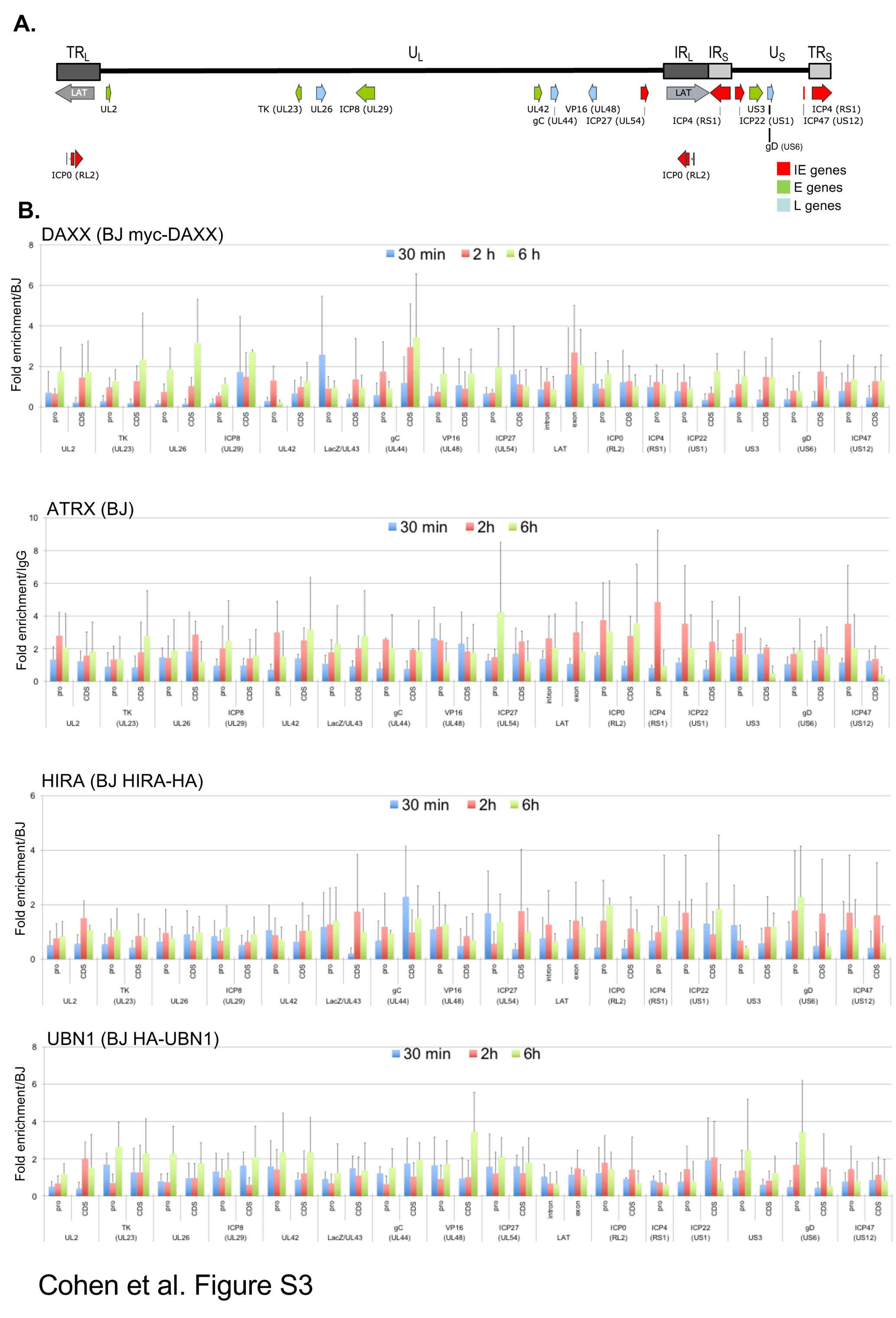

Supplement: S3 Fig — (A) Schematic localization of the HSV-1 genome and of the loci analyzed by quantitative PCR (qPCR). UL: Unit Long, US: Unit Short, TRL: Terminal Repeat Long, TRS: Terminal Repeat Short, IRL: Inverted Repeat Long, IRS: Inverted Repeat Short. Immediate early (IE/α) genes (red), early (E/β) genes (green), late (L/γ) genes (blue). (B) ChIP performed in in1374-infected normal BJ cells or in1374-infected BJ cells expressing tagged versions of DAXX, HIRA, or UBN1. Anti-myc (DAXX) or anti-HA (HIRA and UBN1) antibodies were used. Infections were performed for 30 min (blue), 2 h (red), 6 h (green). For ATRX, a native antibody was used, and the results were compared to ChIP with IgG as control. Analyzed viral loci were described previously. Means from three independent experiments ± SD. (TIF) [file ppat.1007313.s003.tif]

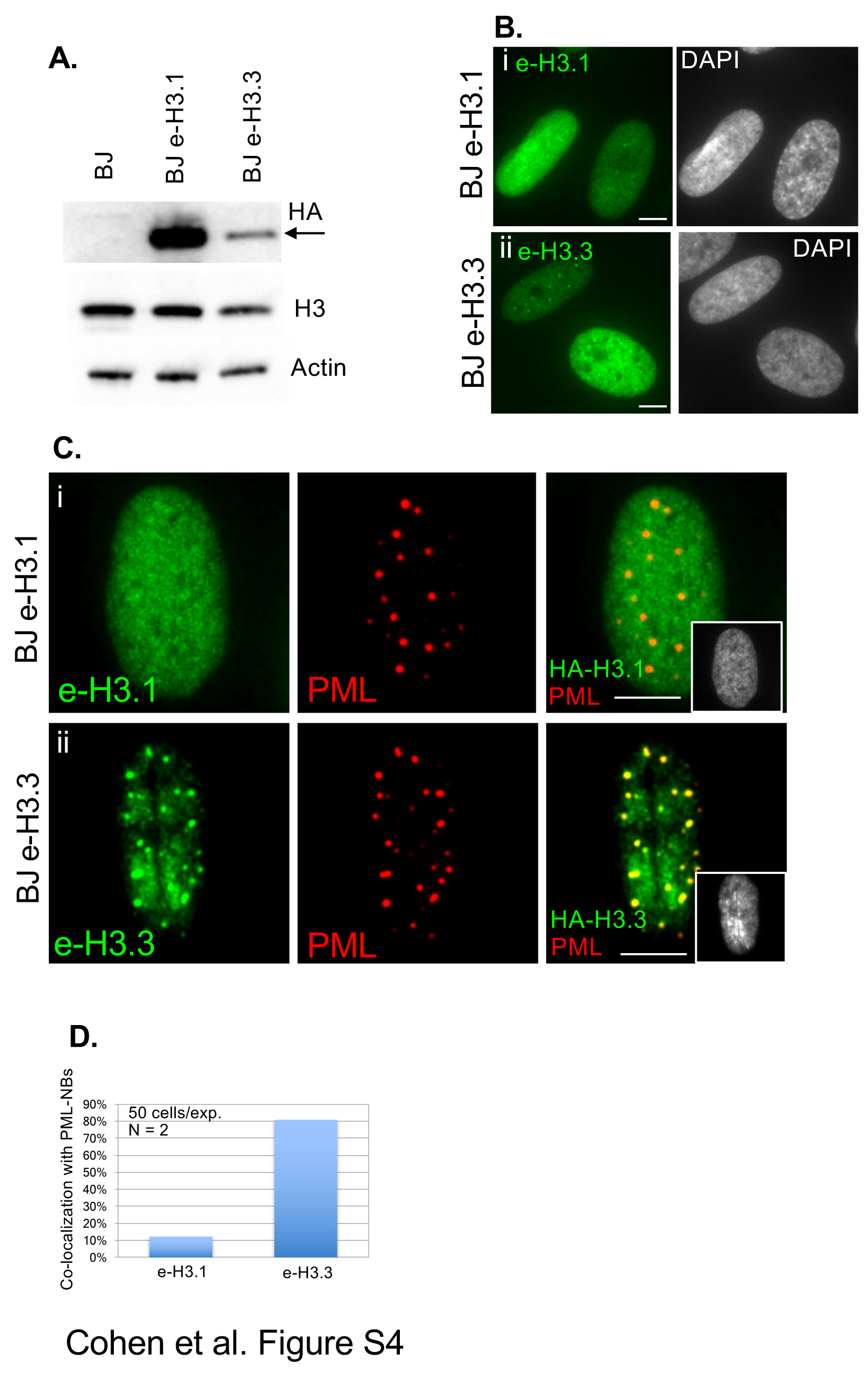

Supplement: S4 Fig — (A) Detection of the protein expression by WB using the anti-HA antibody. Endogenous histone H3 was detected as a control. Actin was detected as a loading control. (B) Detection of e-H3.1 (i) and e-H3.3 (ii) by immunofluorescence. (C) Co-detection of e-H3.1 (i) and e-H3.3 (ii) (green) with PML (red). Nuclei are detected with DAPI (insets, gray) E-H3.3, unlike e-H3.1, co-localizes with PML NBs. Scale bars = 5 μm. (D) Quantification of the immunofluorescence experiments performed in (C). Means from two independent experiments. (TIF) [file ppat.1007313.s004.tif]

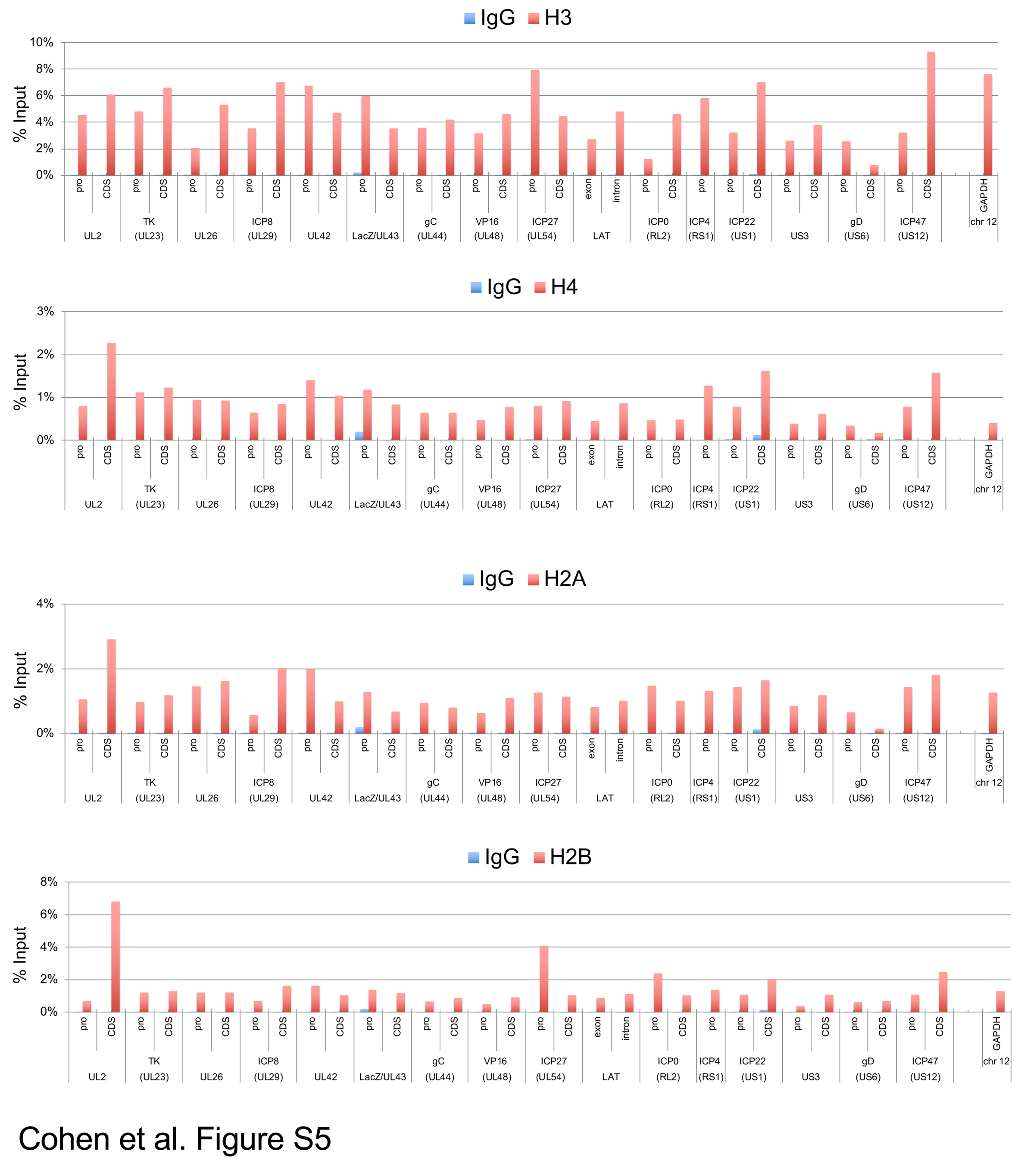

Supplement: S5 Fig — ChIP performed in in1374-infected BJ cells at 24 hpi. Anti-H3, H4, H2A and H2B antibodies were used for ChIP experiments. Analyzed viral loci were described previously. Cellular locus glyceraldehyde 3-phosphate dehydrogenase (GAPDH) was analyzed as control. (TIF) [file ppat.1007313.s005.tif]

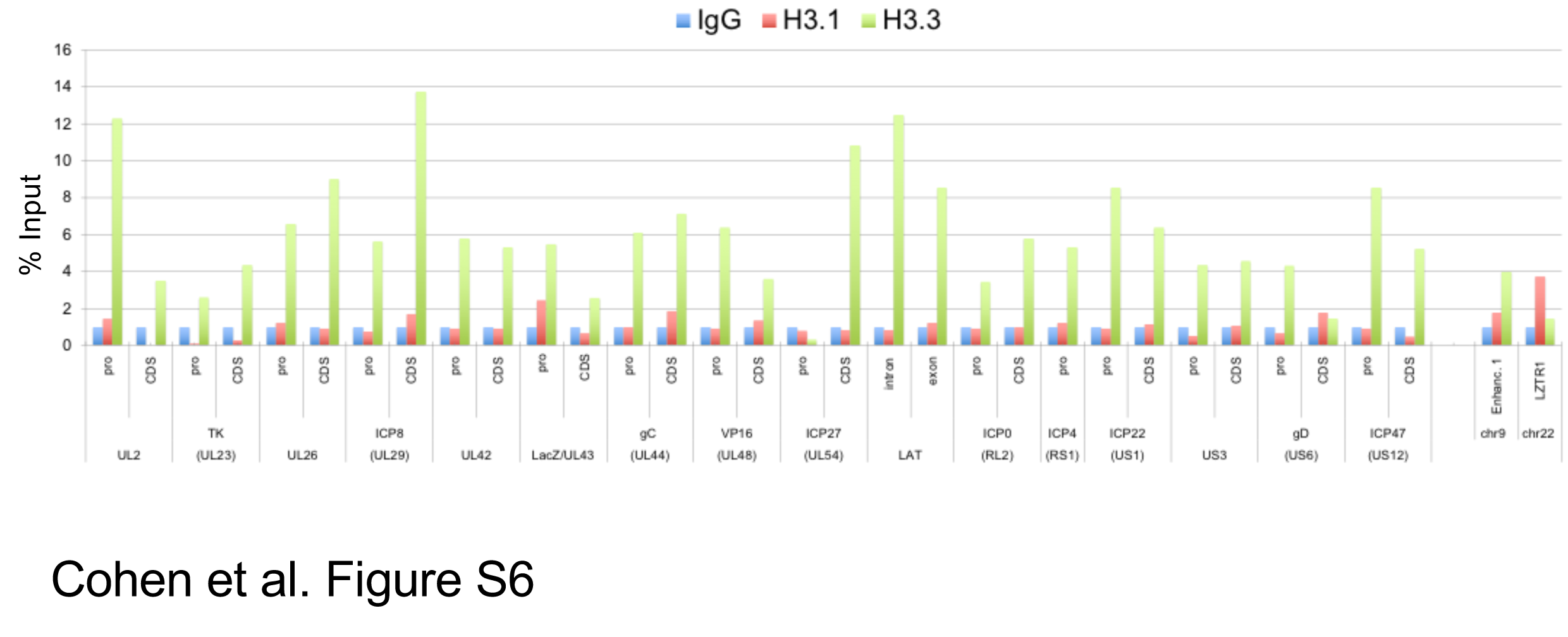

Supplement: S6 Fig — ChIP performed in in1374-infected BJ cells using control IgG (blue), anti-H3.1/2 (red), or anti-H3.3 (green) antibodies. Infections were performed for 24 h. Analyzed viral loci were described previously. Cellular loci Enhancer 1 (Enh.1), and leucine-zipper-like transcriptional regulator 1 (LZTR1) are positive controls for deposition of H3.3 and H3.1, respectively. (TIF) [file ppat.1007313.s006.tif]

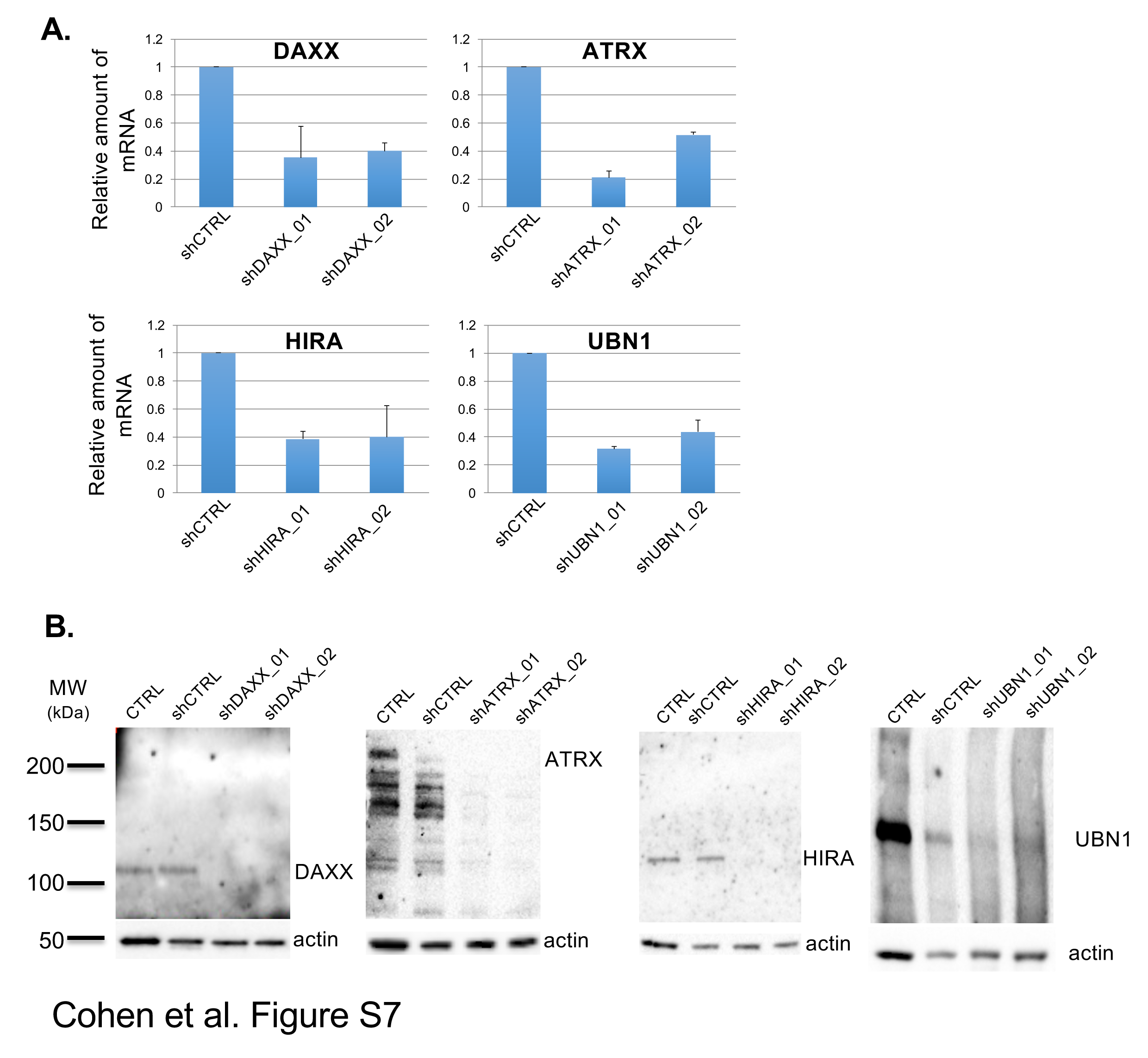

Supplement: S7 Fig — (A) BJ cells were transduced with shRNA-expressing lentiviruses before analysis 48 h post-transduction. RT-qPCR to detect DAXX, ATRX, HIRA, and UBN1 mRNA was performed, and the results were compared to a control shRNA (shCTRL). Means from three independent experiments ± SD. The Student’s t-test was applied to assess the significance of the results. * = p< 0.05, ** = p< 0.01. (B) WB for detection of decreases in DAXX, ATRX, HIRA, and UBN1 proteins in normal BJ cells or BJ cells transduced with shRNA-expressing lentiviruses (48 h post-transduction). Actin was detected as a loading control. Two shRNAs were tested for each protein. (TIF) [file ppat.1007313.s007.tif]

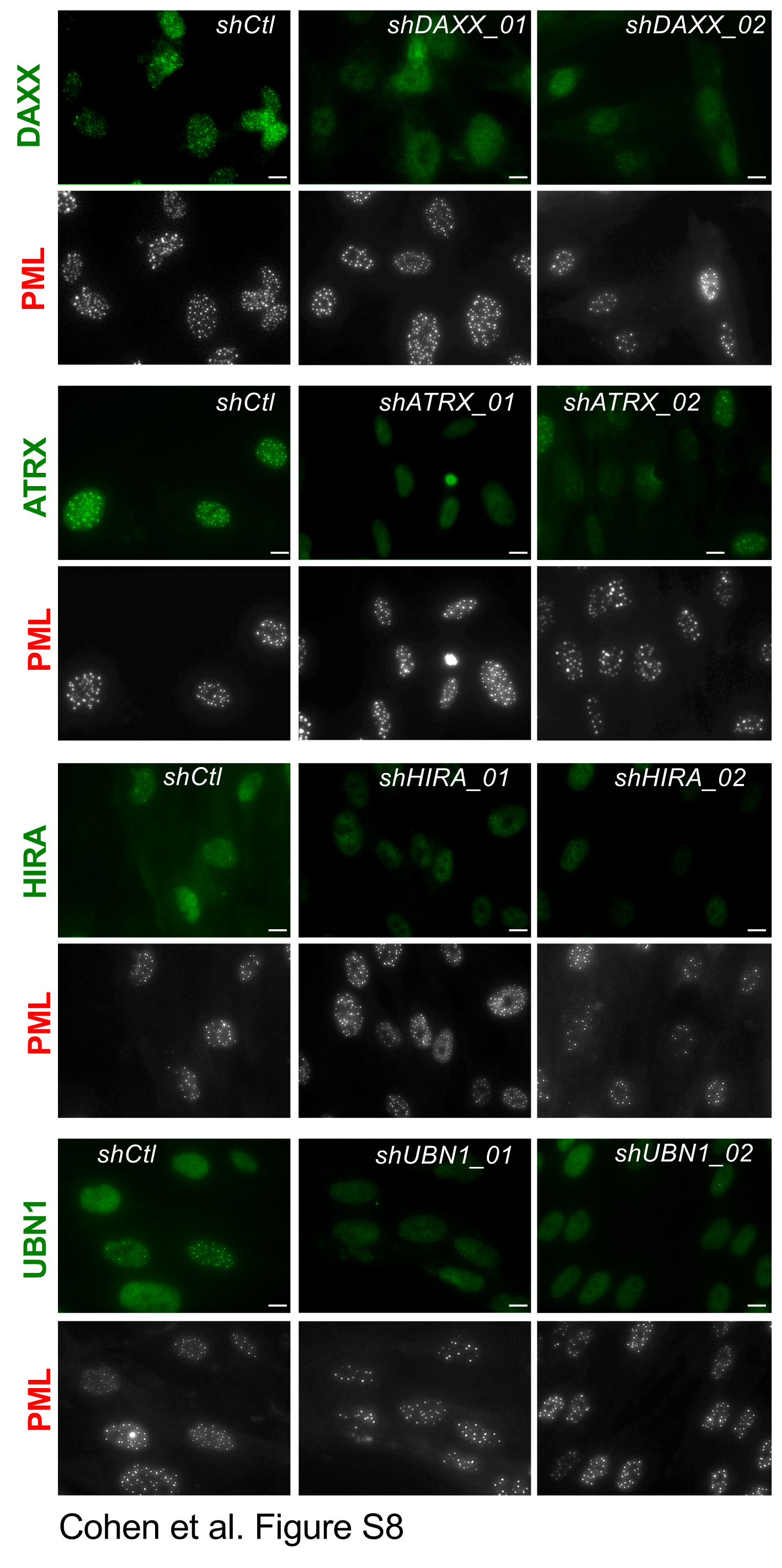

Supplement: S8 Fig — BJ cells were transduced with shRNA-expressing lentiviruses before analysis 48 h post-transduction. Immunofluorescences were performed to detect DAXX, ATRX, HIRA, and UBN1 (green) and PML (gray, red). Two shRNAs were tested for each protein. Scale bars = 5 μm. (TIF) [file ppat.1007313.s008.tif]

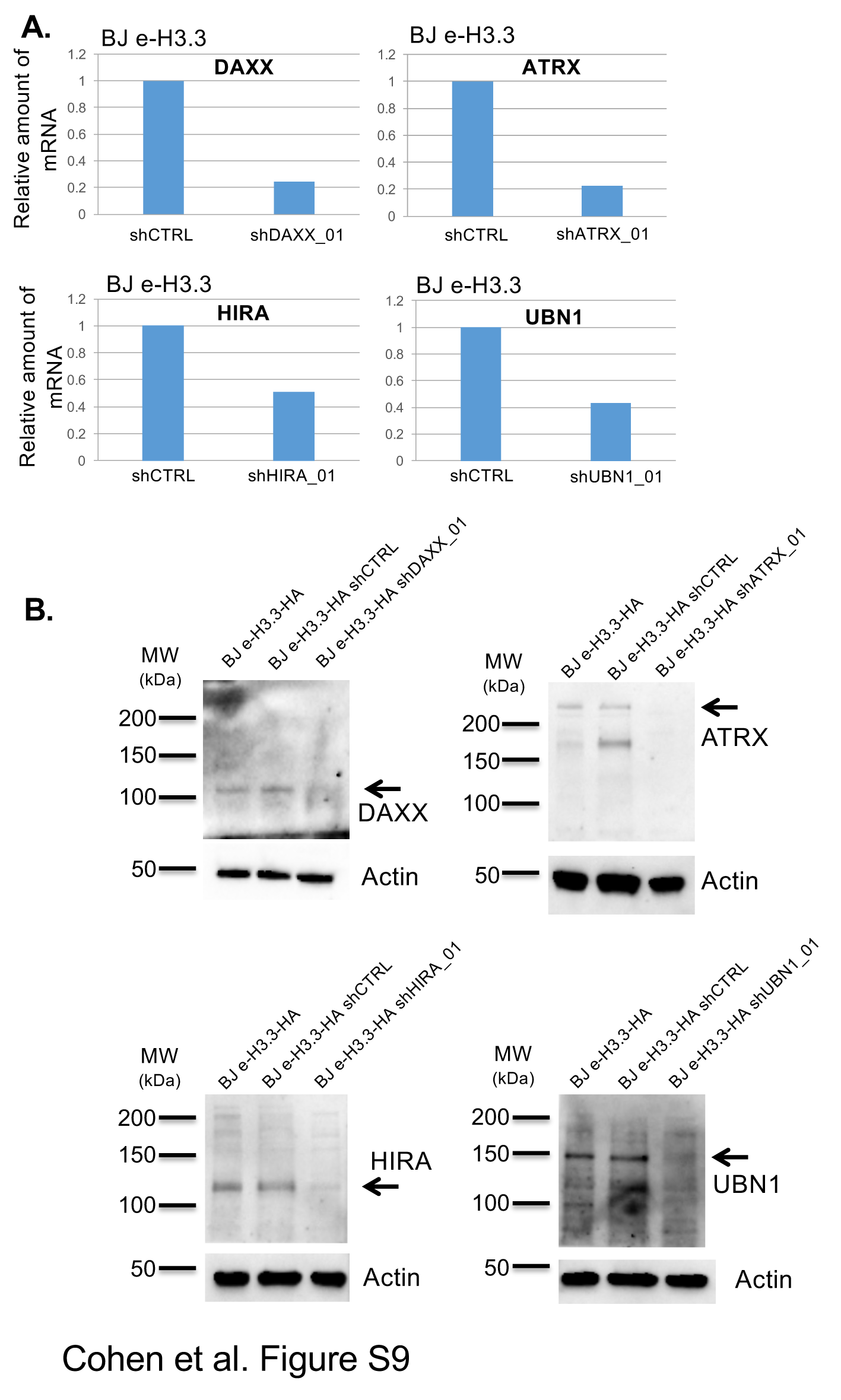

Supplement: S9 Fig — H3.3-expressing BJ cells were transduced with shRNA-expressing lentiviruses before analysis 48 h post-transduction. (A) RT-qPCR to quantify DAXX, ATRX, HIRA, and UBN1 mRNA was performed, and the results were compared to a control shRNA (shCTRL). Data represent means from two independent experiments. (B) WB for detection of decreases in ATRX, HIRA, and UBN1 proteins (48 h post-transduction) in normal e-H3.3-expressing BJ cells or e-H3.3-expressing BJ cells transduced with shRNA-expressing lentiviruses. Actin was detected as a loading control. (TIF) [file ppat.1007313.s009.tif]

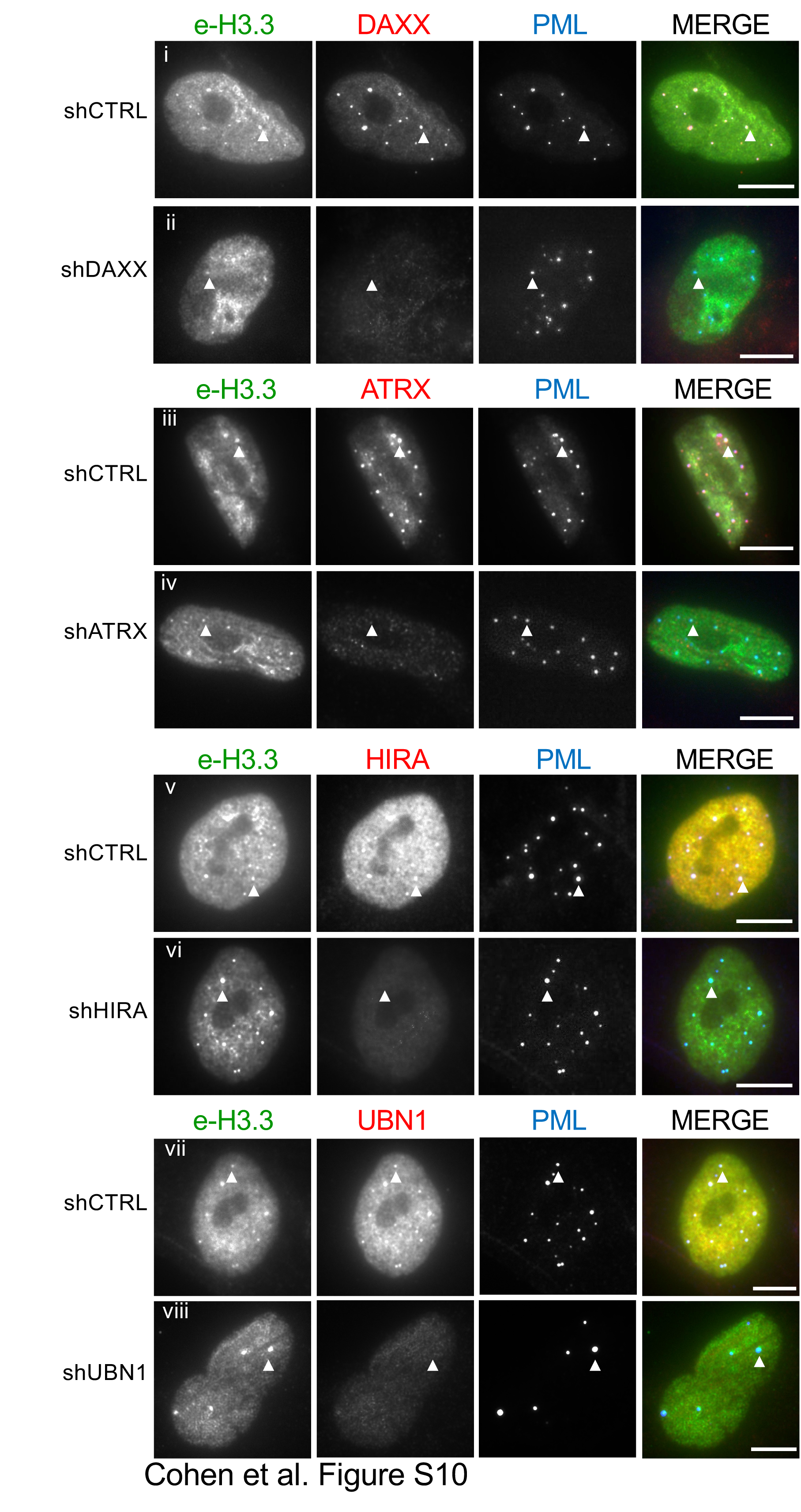

Supplement: S10 Fig — Immunofluorescence experiments performed in e-H3.3-expressing BJ cells transduced with a lentivirus expressing a control shRNA (shCTRL, i, iii, v, vii) or a shRNA targeting DAXX (ii), ATRX (iv), HIRA (vi), or UBN1 (viii). E-H3.3 (gray, green); DAXX, ATRX, HIRA, UBN1 (gray, red); and PML (gray, blue) were detected. For the staining, a rat anti-HA mAb was used to detect e-H3.3, a rabbit polyclonal for the detection of DAXX, ATRX (i-iv), or PML (v-viii), and a mouse mAb for the detection of HIRA, UBN1 (v-viii) or PML (i-iv). Arrowheads point out examples of e-H3.3 co-localization with PML NBs in each sample. Scale bars = 5 μm. (TIF) [file ppat.1007313.s010.tif]

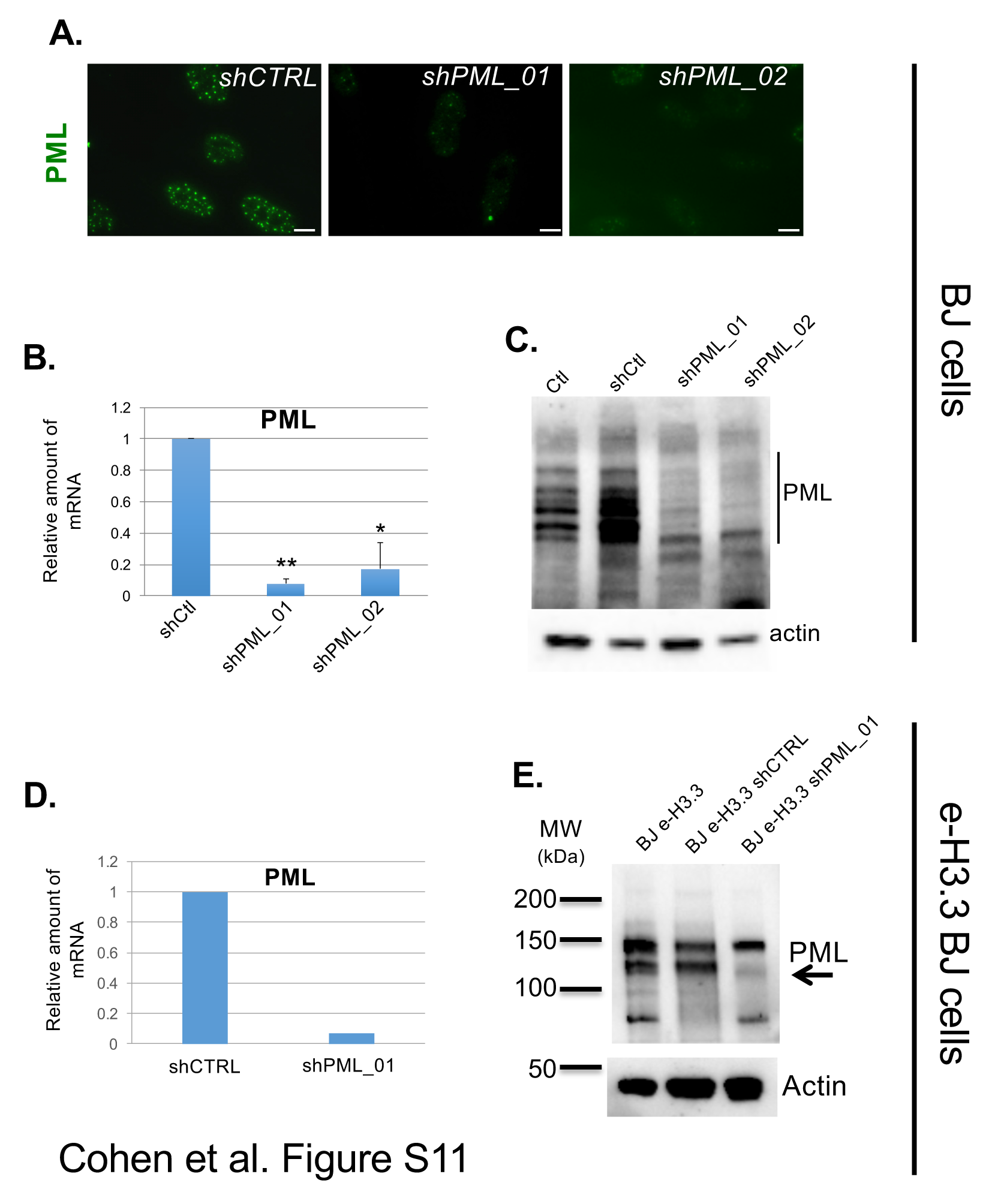

Supplement: S11 Fig — Normal (A-C) or e-H3.3-expressing (D and E) BJ cells were transduced with a lentivirus expressing a control shRNA (shCTRL) or PML shRNAs (shPML) before analysis. Two different shRNAs were validated in normal BJ cells. (A) Immunofluorescence to detect the PML NB signal. Scale bars = 5 μm. (B) RT-qPCR to quantify PML mRNA. Means from three independent experiments ± SD. The Student’s t-test was applied to assess the significance of the results. * = p< 0.05, ** = p< 0.01. (C) WB to detect PML protein. (D) RT-qPCR to quantify PML mRNA. Means from two independent experiments. (E) WB to detect PML protein. (TIF) [file ppat.1007313.s011.tif]

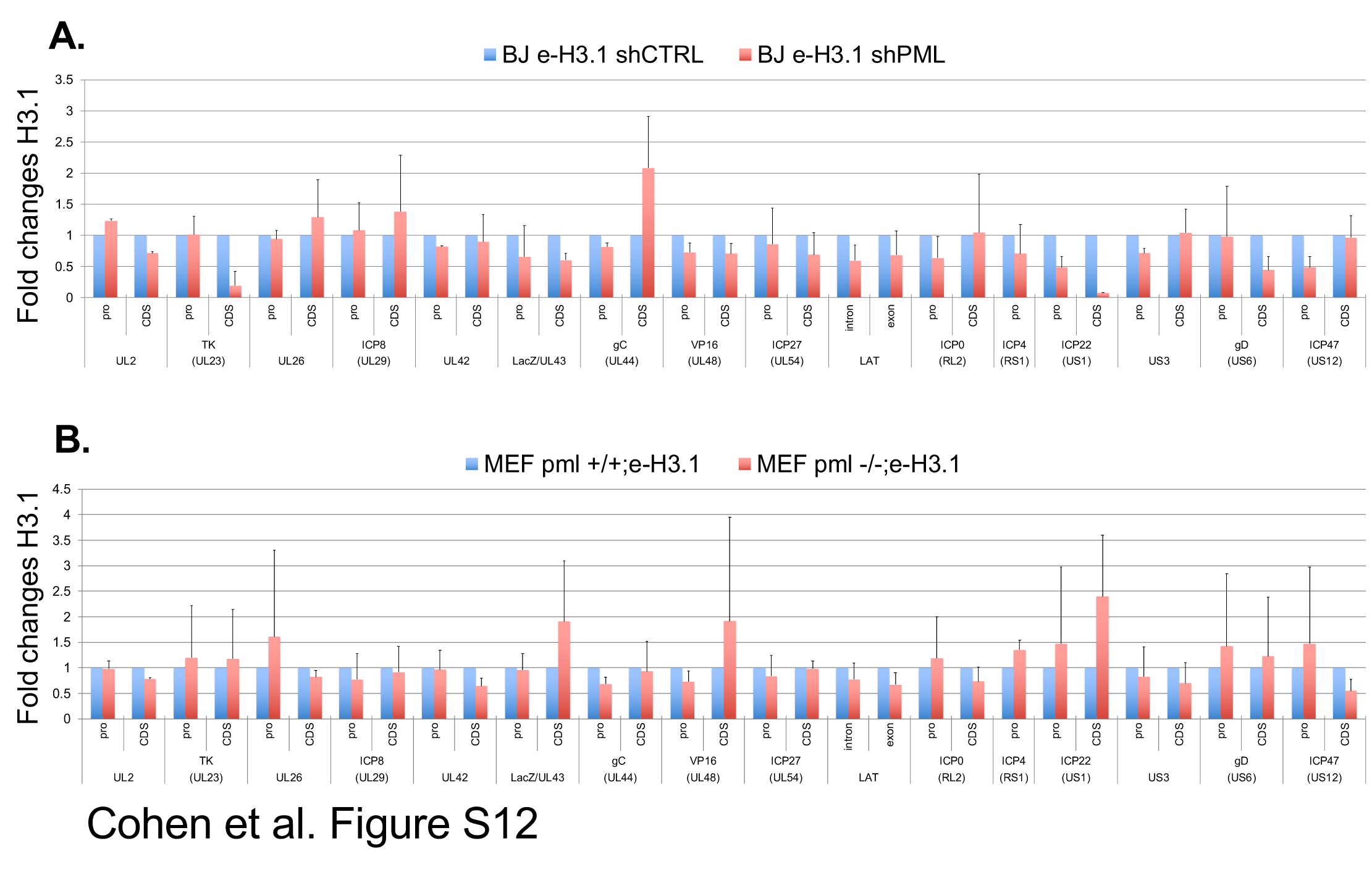

Supplement: S12 Fig — ChIP for the detection of e-H3.1 associated with HSV-1 in e-H3.1-expressing BJ cells previously transduced with a lentivirus expressing a shRNA control (shCTRL, blue) or a PML shRNA (shPML, red) (A) or e-H3.1-expressing MEF pml-/- cells (B). Cells were infected with in1374 for 24 h. Anti-HA antibody was used for the ChIP experiments. The analyzed viral loci were described previously. Data represent means from two independent experiments ± SD. (TIF) [file ppat.1007313.s012.tif]

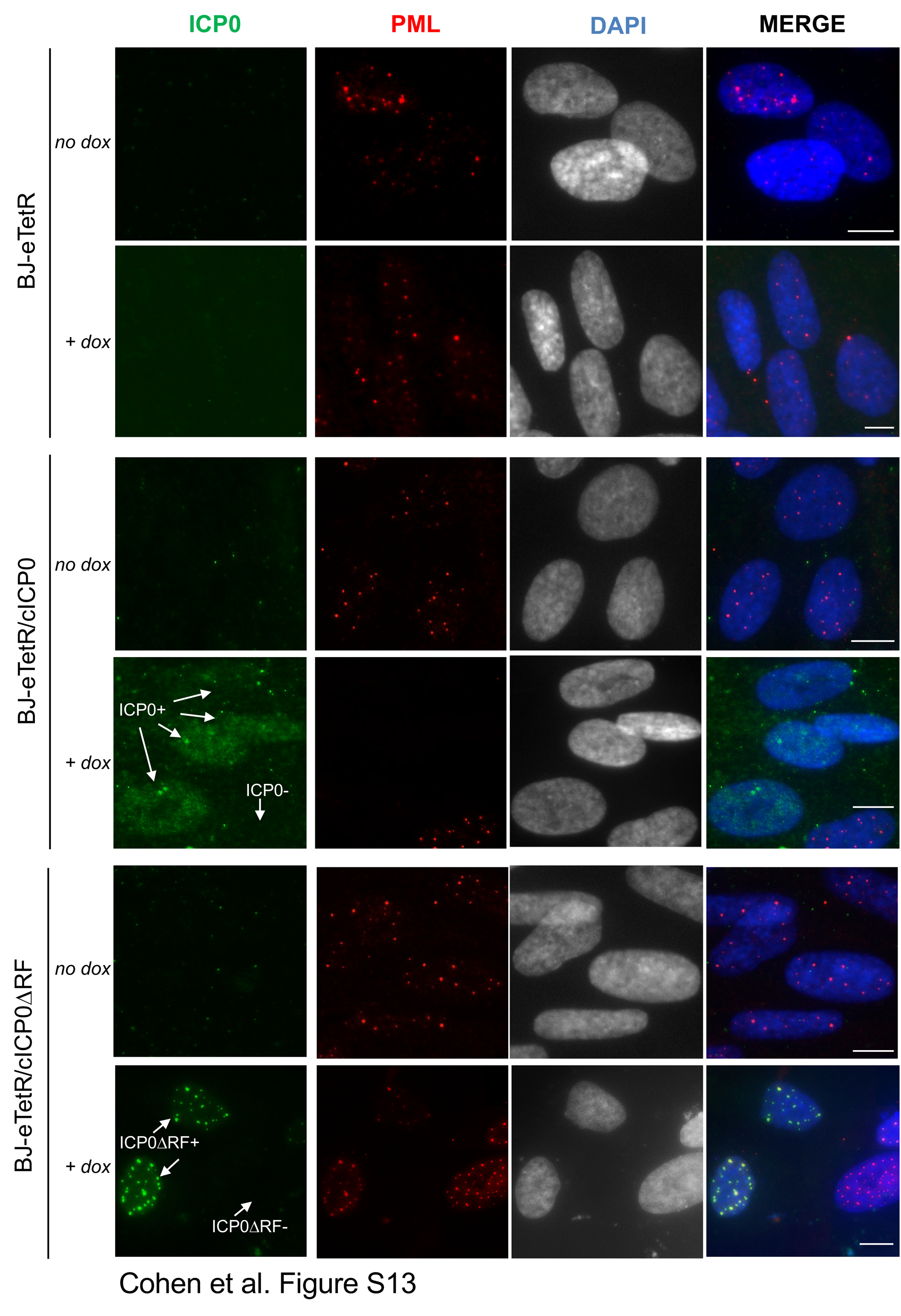

Supplement: S13 Fig — ICP0 or ICP0μRF (green), PML (red), and nuclei (DAPI, gray/blue) are detected. Expression of ICP0 but not ICP0μRF induces the disappearance of PML-NBs. Scale bars = 5 μm. (TIF) [file ppat.1007313.s013.tif]

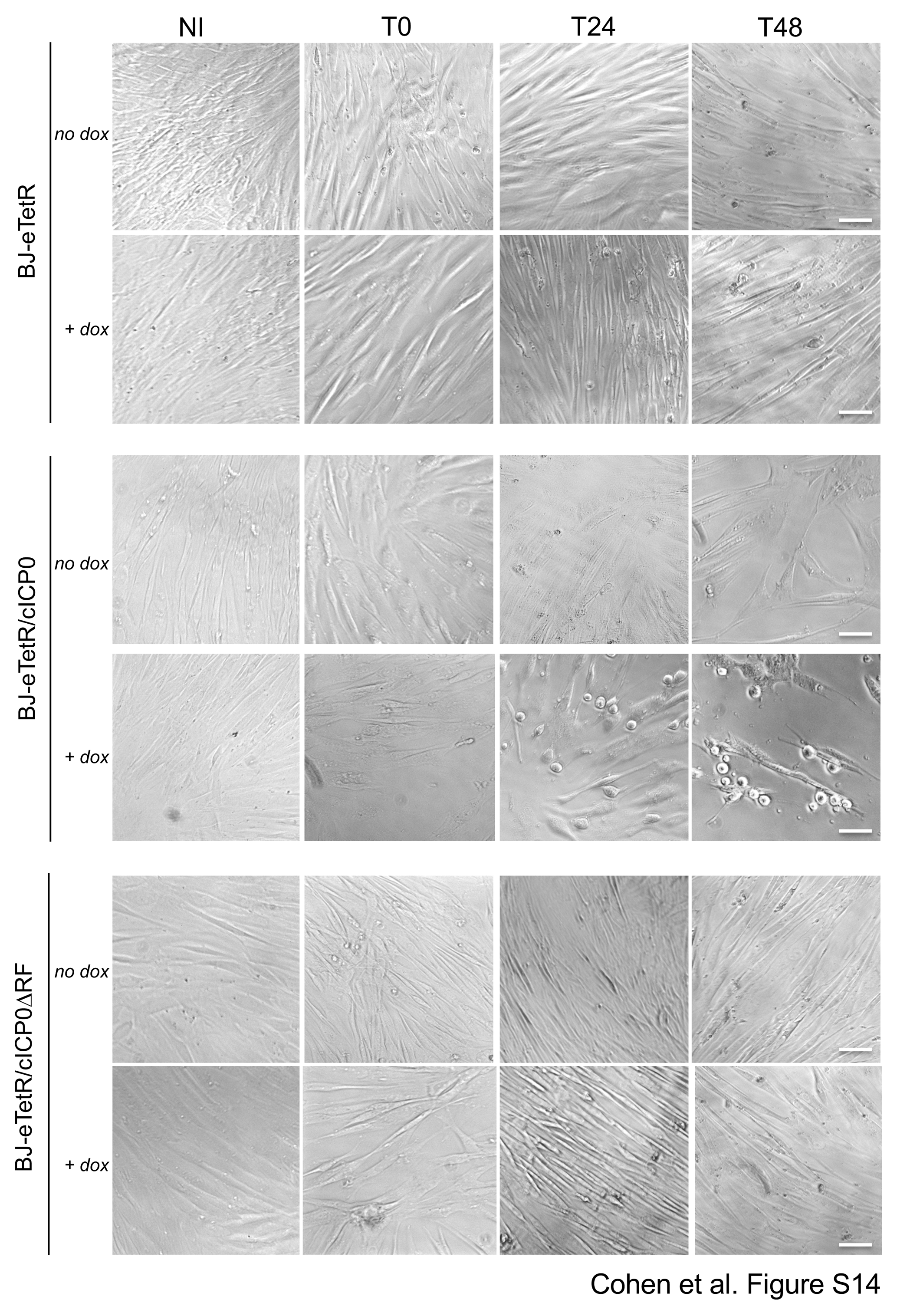

Supplement: S14 Fig — Expression of ICP0, but not ICP0μRF or doxycycline alone, induces a cytopathic effect from T24. Scale bars = 50 μm. (TIF) [file ppat.1007313.s014.tif]

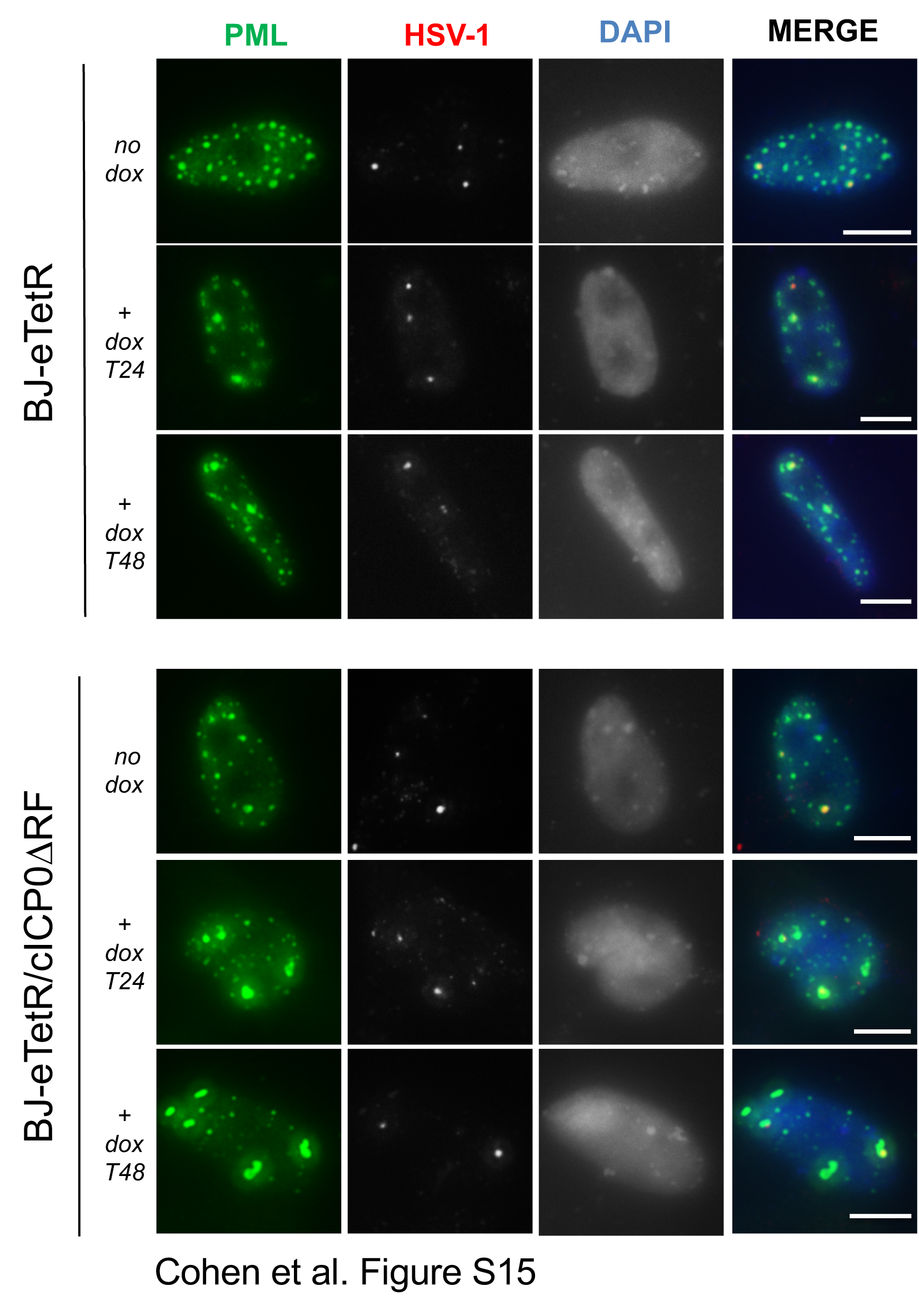

Supplement: S15 Fig — PML (green), HSV-1 genomes (gray/red), and nuclei (DAPI, gray/blue) are detected. Scale bars = 5 μm. (TIF) [file ppat.1007313.s015.tif]

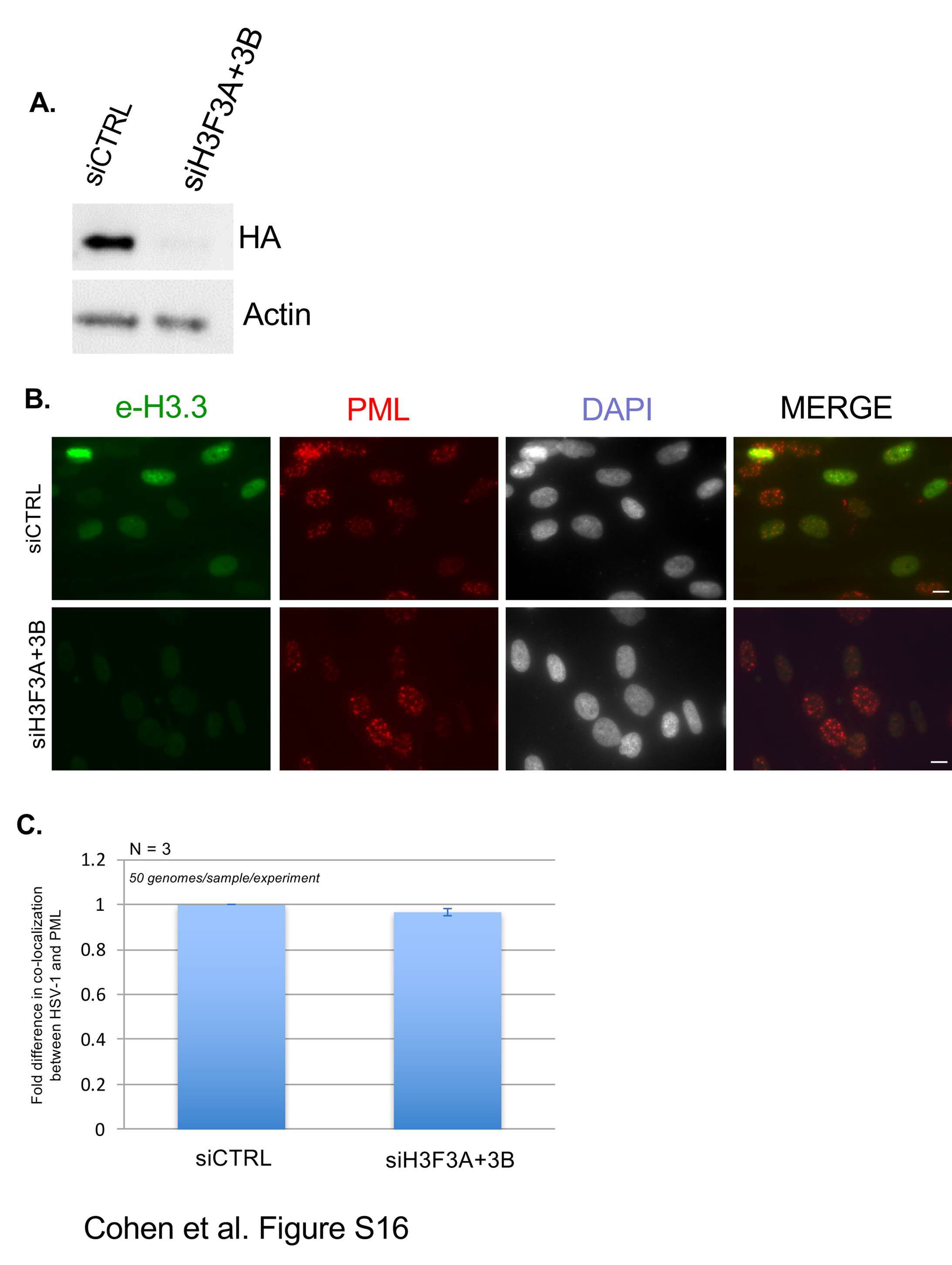

Supplement: S16 Fig — (A) WB to visualize the depletion of H3.3 in e-H3.3-expressing BJ cells. A combination of two siRNAs targeting H3.3 transcripts from both H3.3-encoding genes (H3F3A and H3F3B) were used for the depletion of H3.3. Actin was detected as a loading control. (B) Immunofluorescence performed in e-H3.3-expressing BJ cells transfected with control (siCTRL) or H3.3 (siH3F3A+3B) siRNAs. E-H3.3 (green), and PML (red) were detected. Nuclei were detected with DAPI (gray). Scale bars = 5 μm. (C) Quantifications of co-localizations of HSV-1 genomes with PML issued from immuno-FISH experiments performed in in1374-infected BJ cells (2 dpi) previously transfected with control (siCTRL) or H3.3 (siH3F3A+3B) siRNAs. Means from three independent experiments ± SD. The data suggest that vDCP-NBs are independent of H3.3 chromatinization of the latent/quiescent HSV-1 genomes for their formation. (TIF) [file ppat.1007313.s016.tif]

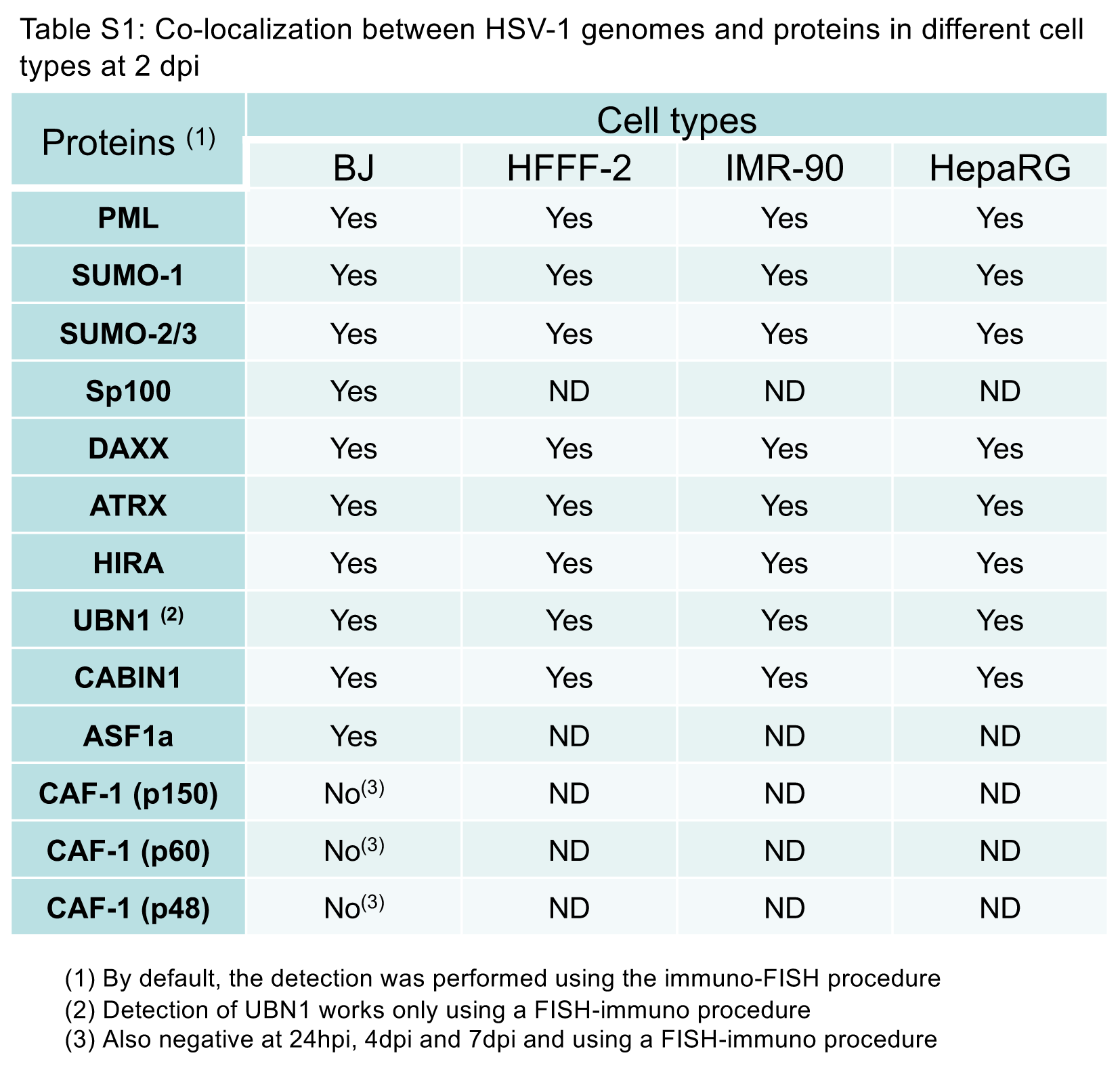

Supplement: S1 Table — (TIF) [file ppat.1007313.s017.tif]

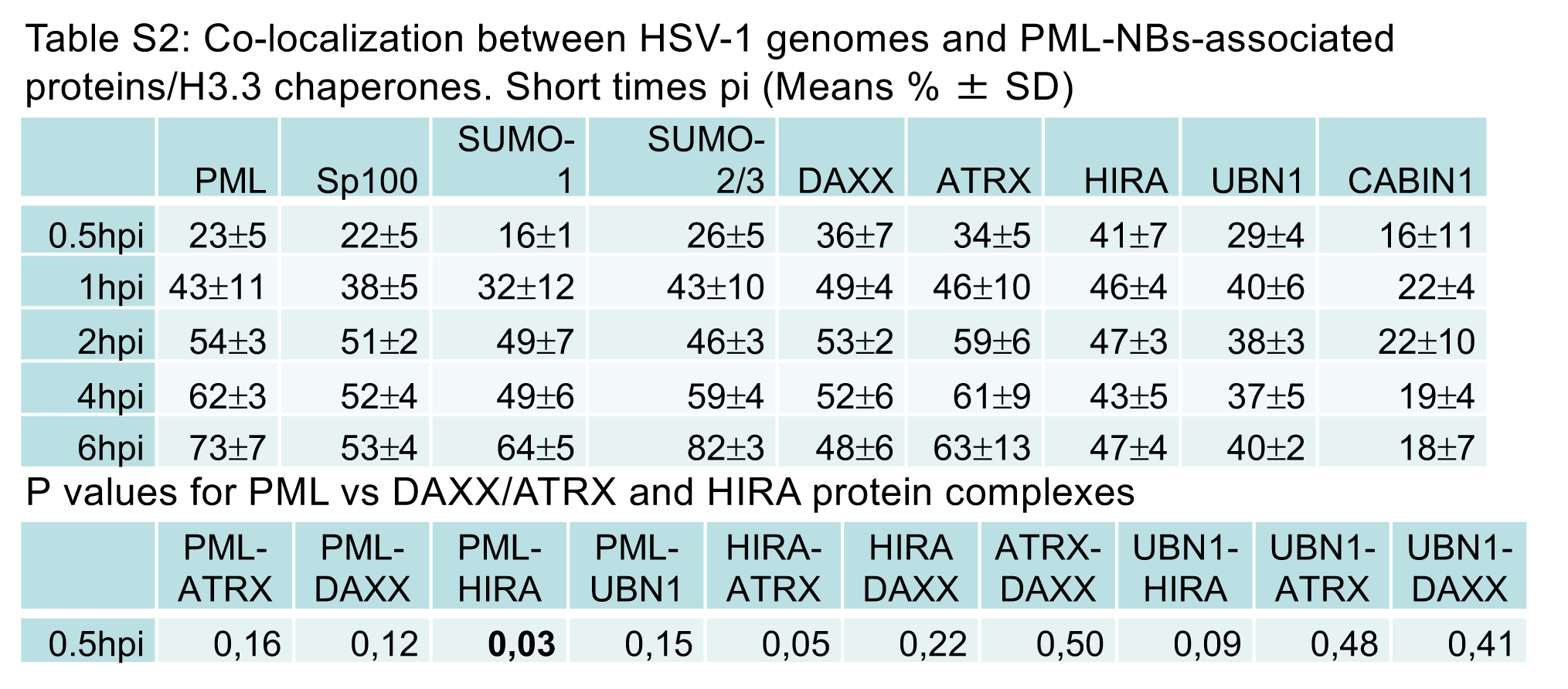

Supplement: S2 Table — Short times pi (Means % ± SD). (TIF) [file ppat.1007313.s018.tif]

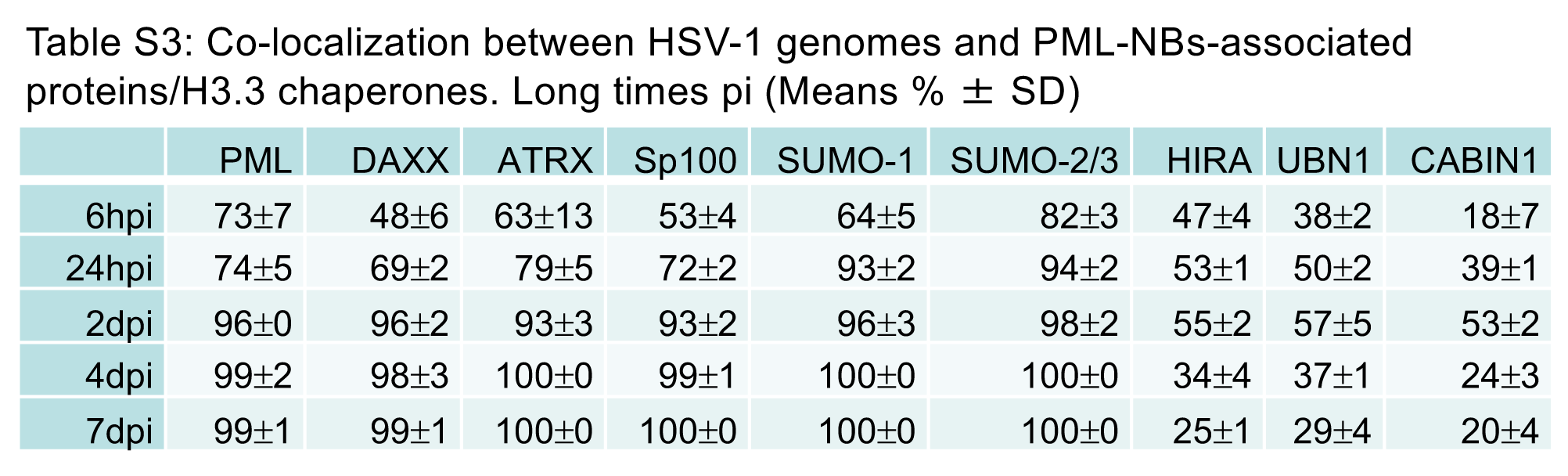

Supplement: S3 Table — Long times pi (Means % ± SD). (TIF) [file ppat.1007313.s019.tif]

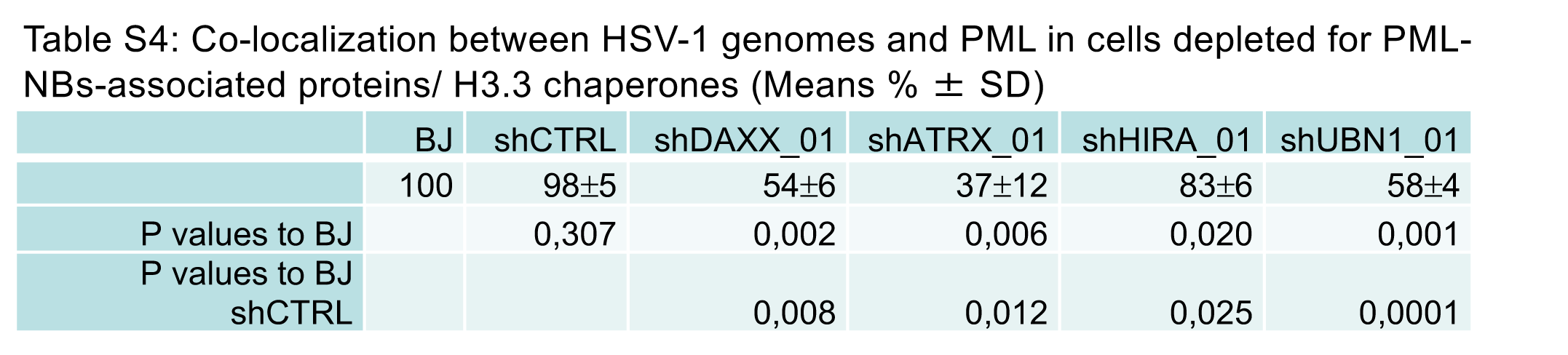

Supplement: S4 Table — (TIF) [file ppat.1007313.s020.tif]

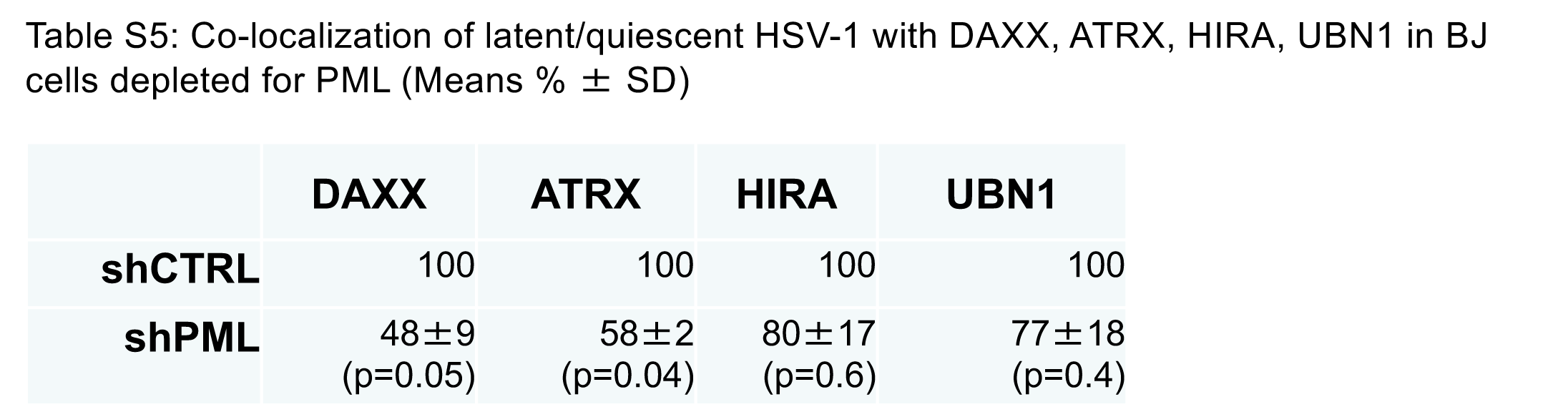

Supplement: S5 Table — (TIF) [file ppat.1007313.s021.tif]
